# Progression of Gastrointestinal Injury During Antiplatelet Therapy After Percutaneous Coronary Intervention: A Secondary Analysis of the OPT-PEACE Randomized Clinical Trial

**DOI:** 10.1001/jamanetworkopen.2023.43219

**Published:** 2023-11-17

**Authors:** Chen He, Yi Li, Xi Jiang, Meng-Ni Jiang, Xian-Xian Zhao, Shu-Ren Ma, Dan Bao, Miao-Han Qiu, Jie Deng, Jin-Hai Wang, Peng Qu, Chun-Meng Jiang, Shao-Bin Jia, Shao-Qi Yang, Lei-Sheng Ru, Jia Feng, Wei Gao, Yong-Hui Huang, Ling Tao, Ying Han, Kan Yang, Xiao-Yan Wang, Wen-Juan Zhang, Bang-Mao Wang, Yue Li, You-Lin Yang, Jun-Xia Li, Jian-Qiu Sheng, Yi-Tong Ma, Min Cui, Si-Cong Ma, Xiao-Zeng Wang, Zhao-Shen Li, Zhuan Liao, Ya-Lin Han, Gregg W. Stone

**Affiliations:** 1 Changhai Hospital, Naval Medical University, Shanghai, China; 2 General Hospital of Northern Theater Command, Shenyang, China; 3 Second Affiliated Hospital of Xi’an Jiaotong University, Xi’an, China; 4 Second Affiliated Hospital of Dalian Medical University, Dalian, China; 5 General Hospital of Ningxia Medical University, Yinchuan, China; 6 No. 980 Hospital of Joint Logistical Support Force, Shijiazhuang, China; 7 Peking University Third Hospital, Beijing, China; 8 Xijing Hospital of Air Force Medical University, Xi’an, China; 9 Third Xiangya Hospital of Central South University, Changsha, China; 10 General Hospital of Tianjin Medical University, Tianjin, China; 11 First Affiliated Hospital of Harbin Medical University, Harbin, China; 12 Seventh Medical Center of the General Hospital of the People’s Liberation Army, Beijing, China; 13 The First Affiliated Hospital of Xinjiang Medical University, Urumqi, China; 14 Mount Sinai Heart and the Cardiovascular Research Foundation, Icahn School of Medicine at Mount Sinai, New York, New York

## Abstract

**Importance:**

Gastrointestinal injury progression induced by antiplatelet therapy in patients after percutaneous coronary intervention (PCI) has not been well studied.

**Objective:**

To assess the association of aspirin, clopidogrel, and their combination with gastrointestinal injury progression among patients without high bleeding risk after PCI.

**Design, Setting, and Participants:**

This secondary analysis assessed data from the Optimal Antiplatelet Therapy for Prevention of Gastrointestinal Injury Evaluated by ANKON Magnetically Controlled Capsule Endoscopy (OPT-PEACE) double-masked, placebo-controlled, multicenter randomized clinical trial. The OPT-PEACE trial was conducted at 28 centers in China, and recruitment took place from July 13, 2017, to July 13, 2019. The trial included patients with stable coronary artery disease or acute coronary syndromes without ST-segment elevation after PCI. Statistical analysis was conducted from September 13, 2022, to January 23, 2023.

**Interventions:**

Patients underwent magnetically controlled capsule endoscopy (MCE) at baseline and after 6 months of dual antiplatelet therapy (DAPT) with aspirin (100 mg/d) plus clopidogrel (75 mg/d). Those with no evidence of gastrointestinal ulcers or bleeding (ie, the intention-to-treat [ITT] cohort) were randomized (1:1:1) to aspirin (100 mg/d) plus matching placebo (aspirin alone), clopidogrel (75 mg/d) plus matching placebo (clopidogrel alone), or DAPT for an additional 6 months. A third MCE was performed 12 months after PCI.

**Main Outcomes and Measures:**

The primary outcome was the rate of gastric injury progression as assessed with the results of the 3 MCEs (at baseline, 6 months, and 12 months) in the modified intention-to-treat (mITT) population. The key secondary outcome was the rate of small-intestinal injury progression. Gastric or small-intestinal injury progression was defined as a quantitative increase in erosions or ulcers between the second and third MCEs (at 6 and 12 months, respectively).

**Results:**

This study included the 394 patients in the mITT cohort. Their mean (SD) age was 56.9 (8.7) years, and most were men (296 [75.1%]). A total of 132 patients were randomized to aspirin alone, 132 to clopidogrel alone, and 130 to DAPT. Gastric injury progression occurred in 49 aspirin users (37.1%), 64 clopidogrel users (48.5%), and 69 DAPT users (53.1%) (*P* = .02), reflecting a lower rate of gastric injury progression among aspirin users vs DAPT users (risk ratio [RR], 0.70 [95% CI, 0.49-0.99]; *P* = .009). No significant difference was observed between clopidogrel alone and DAPT (48.5% vs 53.1%; *P* = .46) or between aspirin alone and clopidogrel alone (37.1% vs 48.5%; *P* = .06). A total of 51 aspirin users (38.6%), 65 clopidogrel users (49.2%), and 71 DAPT users (54.6%) (*P* = .03) developed progressive small-intestinal injury, reflecting a lower rate of small-intestinal injury among aspirin users vs DAPT users (RR, 0.71 [95% CI, 0.50-0.99]; *P* = .01). No difference was observed between patients treated with clopidogrel vs DAPT (49.2% vs 54.6%; *P* = .38) or with aspirin vs clopidogrel (38.6% vs 49.2%; *P* = .08).

**Conclusions and Relevance:**

In this secondary analysis of a randomized clinical trial, ongoing use of aspirin, clopidogrel, or their combination between 6 and 12 months after PCI was associated with progressive gastric and small-intestinal injury in a substantial proportion of patients, more so with DAPT than with monotherapy. Clopidogrel was at least as likely as aspirin to induce gastrointestinal injury progression. Future research is warranted to determine what impact the findings from MCEs would have on decision-making of antiplatelet therapy.

**Trial Registration:**

ClinicalTrials.gov Identifier: NCT03198741

## Key Points

**Question:**

Are different antiplatelet regimens associated with gastric and small-bowel injury progression among long-term antiplatelet agent users without high gastrointestinal bleeding (GIB) risk?

**Findings:**

In this secondary analysis of a randomized clinical trial with 394 participants, aspirin, clopidogrel, or their combination were associated with progressive gastric and small-intestinal injury in a substantial proportion of patients after percutaneous coronary intervention, particularly during dual antiplatelet therapy compared with monotherapy.

**Meaning:**

The findings of this study suggest that continuous antiplatelet use, especially with dual therapy, may increase the risk of GIB, even among patients without high risk.

## Teaser

This secondary analysis of a randomized clinical trial assesses the association of aspirin, clopidogrel, and their combination with gastrointestinal injury progression among patients in China without high bleeding risk after percutaneous coronary intervention.

## Introduction

Antiplatelet regimens are widely used for secondary prevention of cardiovascular disease to reduce the risk of recurrent ischemic events after percutaneous coronary intervention (PCI).^1^ For patients without high bleeding risk, practice guidelines recommend use of clopidogrel plus aspirin for at least 12 months after PCI for those with acute coronary syndromes and for at least 6 months for those with stable coronary artery disease to avoid subsequent cardiac events.^2^ However, antiplatelet agents increase the risk of gastrointestinal bleeding (GIB),^3^ with approximately 1% of patients experiencing overt GIB within the first 30 days of therapy.^4^ Long-term antiplatelet therapy results in both upper and lower GIB^5^ and is independently associated with longer hospitalizations and mortality.^6^ Data from randomized and observational studies have shown that treatments that reduce gastric acidity decrease GIB and other gastrointestinal complications of antiplatelet therapy.^7,8^ For patients with a history of peptic ulcer, GIB, and other bleeding risk factors, routine use of proton-pump inhibitors is recommended to mitigate the risk of upper gastrointestinal complications.^9^ Routine prophylaxis for antiplatelet therapy–related gastrointestinal complications is not recommended for individuals without high GIB risk; however, these patients may still progress to high GIB risk.^10^ Recognizing gastrointestinal injury progression during continuous antiplatelet therapy is an important first step prior to developing risk stratification tools to adjust treatment during long-term use.

Despite these considerations, the incidence of gastric and small-intestinal injury progression among patients without high GIB risk has not been well defined. In addition, the effects of different antiplatelet regimens, including aspirin, clopidogrel, or dual antiplatelet therapy (DAPT), on gastric and small-bowel injury progression remain unknown.

To explore gastric and small-intestinal injury progression induced by aspirin, clopidogrel, and DAPT in patients without high GIB risk, we conducted a secondary analysis of the Optimal Antiplatelet Therapy for Prevention of Gastrointestinal Injury Evaluated by ANKON Magnetically Controlled Capsule Endoscopy (OPT-PEACE) trial.^10^ The results of the main trial indicated that patients who received DAPT for 6 months, followed by aspirin or clopidogrel for another 6 months, had a lower incidence of gastrointestinal injury than those who received DAPT for 12 months. We further examined the rate of gastrointestinal injury progression according to serial magnetically controlled capsule endoscopy (MCE) results.

## Methods

### Study Design and Participants

This study is a secondary analysis of the OPT-PEACE double-masked, placebo-controlled, multicenter randomized clinical trial conducted at 28 centers in China.^10,11^ All participants (or their legal representatives) provided written informed consent before enrollment according to local guidelines. The study was approved by local ethics committees in each center and was conducted according to the principles of the Declaration of Helsinki.^12^ The trial protocol and statistical analysis plan are presented in Supplement 1. The study followed the Consolidated Standards of Reporting Trials (CONSORT) reporting guideline.

The OPT-PEACE trial enrolled patients with stable coronary artery disease or acute coronary syndromes without ST-segment elevation after PCI. Recruitment occurred between July 13, 2017, and July 13, 2019. Patients aged 18 to 80 years who planned to use DAPT for at least 6 months were included. Patients with high GIB risk, including those with a history of GIB or peptic ulcer within 24 months, prior gastrointestinal tract or abdominal surgery, kidney dysfunction, or requirement for chronic oral anticoagulation were excluded. A baseline MCE was performed, and patients were excluded if ulcers or bleeding were found. After 6 months of DAPT, patients without overt bleeding or ischemic events underwent a second MCE. Eligible patients without gastrointestinal ulcers or GIB (erosions were permitted) on the first 2 MCEs (ie, the intention-to-treat [ITT] population) were then randomly assigned (1:1:1) to receive enteric-coated aspirin (100 mg/d) plus matching clopidogrel placebo (aspirin alone), clopidogrel (75 mg/d) plus matching aspirin placebo (clopidogrel alone), or aspirin plus clopidogrel (DAPT) for the next 6 months. A third MCE was performed 6 months after randomization (12 months after PCI). A *Helicobacter pylori* breath test was recommended after MCE; the decision of whether to eradicate *H. pylori* was at the physician’s discretion. Serum hemoglobin levels and fecal occult blood were assessed every 2 months during follow-up. Routine use of proton-pump inhibitors or other gastric mucosal protectant agents was not permitted after enrollment except for individuals with clinically overt bleeding.

### Procedure

Magnetically controlled capsule endoscopy was performed with the NaviEC-1000 (ANKON Medical Technologies), a noninvasive, active-controlled screening system for gastrointestinal diseases.^13,14^ The NaviEC-1000 system consists of an endoscopic capsule, a capsule locator, a magnetic guidance system, a computer workstation for real-time viewing and control, and a portable data recorder (eFigure 1 in Supplement 2). The capsule is 26.8 × 11.6 mm in size and weighs 4.8 g. During the gastric examination, images are captured at 2 frames per second (fps), with a resolution of 480 × 480 pixels. The view angle of the capsule is 140°, and the view depth is 0 to 60 mm. The capsule battery life is longer than 8 hours.

All participants were required to fast overnight (>8 hours) and consume 2 L of polyethylene glycol the night before the examination. Patients were asked to ingest 2.5 g of dimethicone 40 minutes before the examination. They were then encouraged to drink 500 to 1000 mL of water before swallowing the capsule.^15^ Patients were then instructed to assume the left lateral decubitus position and to swallow the capsule with a small amount of water. When the capsule entered the stomach, it was rotated and advanced to the fundus and cardiac regions, followed by the gastric body, angulus, antrum, and pylorus under magnetic control.^13,15,16^ After completion of the stomach examination, the capsule switched to small-bowel mode with a capture rate of 2 fps. All operations followed a standardized protocol.^15^ All examinations were conducted by experienced endoscopists who have performed more than 100 MCEs and were blinded to participant randomization. The coded videos of MCEs were reviewed at an independent core laboratory by experienced gastroenterologists who were unaware of group assignment and MCE timing.

### Outcomes

The primary outcome was the rate of gastric injury progression from 6 to 12 months after PCI in the modified intention-to-treat (mITT) population, including those obtaining 3 comprehensive MCE results. Differences reflected the 6-month randomization period to aspirin alone, clopidogrel alone, or continued DAPT. Injuries in the stomach were counted separately based on anatomic region (gastric fundus, body, antrum, or angulus). Gastric injury progression was defined as the quantitative increase in erosions or ulcers^17^ at any region between the second and third MCEs (at 6 and 12 months, respectively). Erosions were defined as flat lesion mucosal breaks with no discernible depth and a diameter of less than 5 mm. Ulcers were defined as mucosal breaks 5 mm or larger in diameter with discernible depth, typically covered with fibrin.^17^

The key secondary outcome was the rate of small-intestinal injury progression among the mITT population during the 6 months after randomization. Injuries in the small intestine were counted separately according to the proximal, middle, and distal small intestine divided by transit time.^18^ Injury in the small intestine included red spots, erosions, and ulcers.^19^ Red spots were defined as red areas of mucosa with preservation of villous architecture. The definitions of erosions and ulcers were the same as for those in the stomach. Small-intestinal injury progression was defined as newly developed red spots and a quantitative increase in mucosal erosions or ulcers at the proximal, middle, or distal small intestine during the period between the second and third MCEs according to the Graham scoring system.^20^

We examined subgroups according to whether a previous gastric or small-intestinal injury was present on the 6-month MCE. In patients with gastric injury after 6 months of DAPT, we examined the incidence of gastric injury progression from randomization to 12 months. Newly developed gastric injury was defined as the de novo development of gastric injury between 6 and 12 months in the group with no gastric injury after 6 months of DAPT. Subgroup analysis was also performed in patients with and without small-intestinal injury at the second (6-month) MCE.

### Statistical Analysis

Analyses were based on the mITT sample, for which comprehensive results of 3 MCEs of the stomach and whole small intestine were obtained. Categorical variables are reported as proportions, which were compared with the χ^2^ test. Continuous variables are reported as means (SDs), which were compared with unpaired *t* tests or the Wilcoxon rank sum test. All *P* values are 2 sided. The principal analyses were performed across all 3 randomized groups, with *P* < .05 considered statistically significant. When the 3 intervention groups (aspirin vs clopidogrel, aspirin vs DAPT, and clopidogrel vs DAPT) were compared, a Bonferroni correction with the critical *P* value set at <.01 was used. The logistic regression for interaction assessed statistically significant differences in subgroups and intervention groups. Risk ratios (RRs) with 98.75% CIs were calculated with logistic regression and asymptotic Wald confidence limits. All statistical analyses were performed using SPSS software, version 23.0 (IBM Corp). Statistical analysis was conducted from September 13, 2022, to January 23, 2023.

## Results

### Patient Characteristics

This secondary analysis of the OPT-PEACE trial included the 394 patients in the mITT cohort randomized to aspirin alone (n = 132), clopidogrel alone (n = 132), or DAPT (n = 130) (Figure and Table 1). Their mean (SD) age was 56.9 (8.7) years; 296 were men (75.1%) and 98 were women (24.9%). Proportions of alcohol consumption (52 of 132 [39.4%] vs 49 of 132 [37.1%] vs 45 of 130 [34.6%]) and *H. pylori* infection (29 of 132 [22.0%] vs 19 of 132 [14.4%] vs 28 of 130 [21.5%]) were comparable among the aspirin, clopidogrel, and DAPT groups, respectively. Other baseline characteristics and medication adherence of the intention-to-treat (ITT) population in the main study were reported previously.^10^ Additional details on the mITT population are presented in eTables 1 and 2 in Supplement 2.

**Figure.**
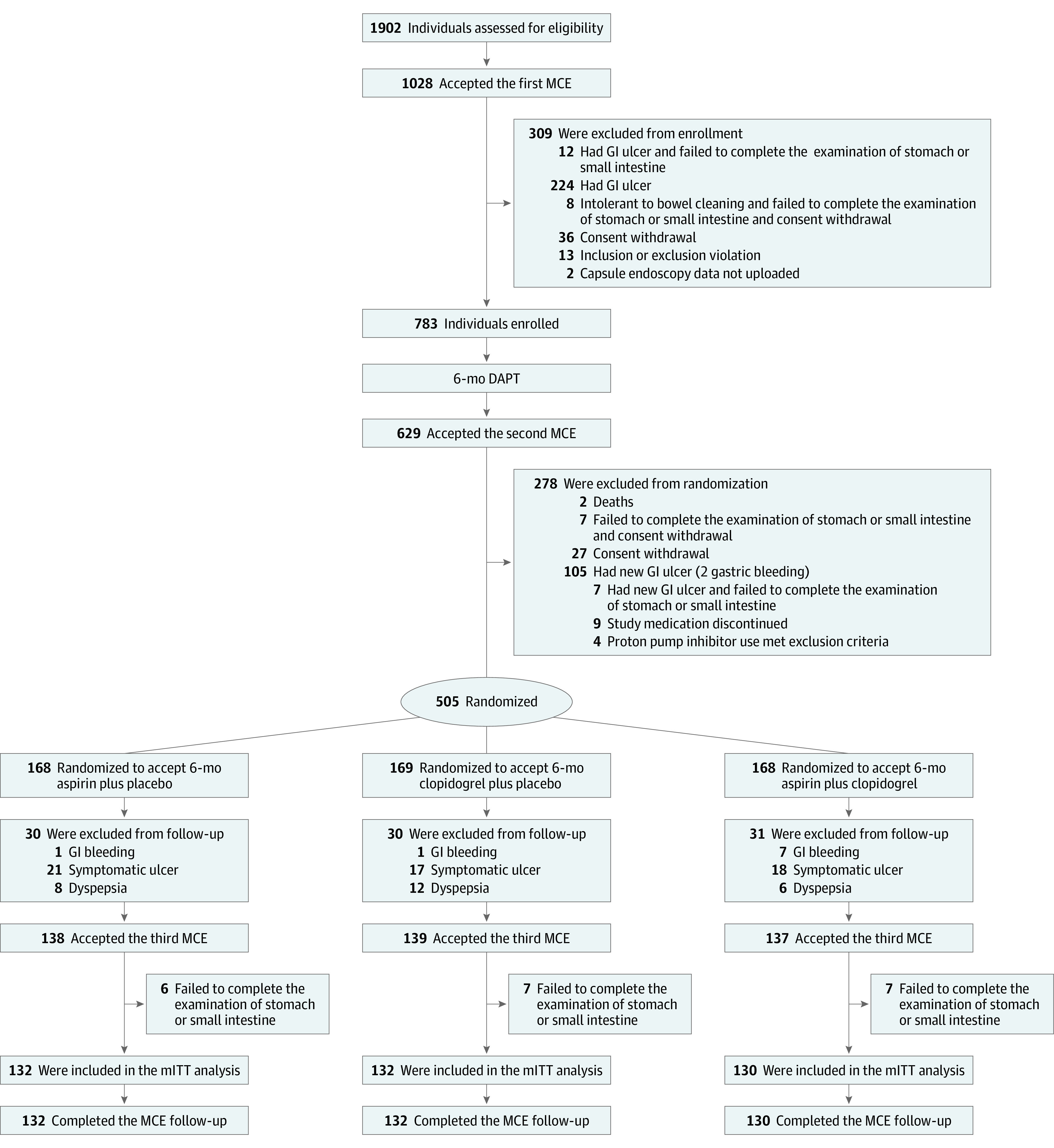
Study Flowchart DAPT indicates dual antiplatelet therapy; GI, gastrointestinal; MCE, magnetically controlled capsule endoscopy; mITT, modified intention-to-treat.

**Table 1.** Baseline Characteristics of the Modified Intention-to-Treat Population^a^

Characteristic	Treatment group		
	Aspirin alone (n = 132)	Clopidogrel alone (n = 132)	DAPT (n = 130)
Age, mean (SD), y	56.8 (8.6)	56.3 (8.1)	57.5 (8.3)
Sex			
Male	100 (75.8)	98 (74.2)	98 (75.4)
Female	32 (24.2)	34 (25.8)	32 (24.6)
BMI, mean (SD)	25.9 (3.0)	26.0 (3.6)	25.5 (2.8)
Hypertension	76 (57.6)	79 (59.8)	85 (65.4)
Diabetes	40 (30.3)	28 (21.2)	26 (20.0)
Hyperlipidemia	13 (9.8)	7 (5.3)	14 (10.8)
Smoking status			
Never	59 (44.7)	65 (49.2)	71 (54.6)
Former	20 (15.2)	19 (14.4)	13 (10.0)
Active	53 (40.2)	48 (36.4)	46 (35.4)
Alcohol use			
Never	80 (60.6)	83 (62.9)	85 (65.4)
Former	9 (6.8)	7 (5.3)	8 (6.2)
Active	43 (32.6)	42 (31.8)	37 (28.5)
Previous myocardial infarction	12 (9.1)	8 (6.1)	9 (6.9)
Previous PCI	17 (12.9)	14 (10.6)	12 (9.2)
Previous stroke	14 (10.6)	16 (12.1)	14 (10.8)
Presentation			
Chronic coronary syndrome	5 (3.8)	5 (3.8)	6 (4.6)
Unstable angina	112 (84.8)	107 (81.1)	109 (83.8)
NSTEMI	15 (11.4)	20 (15.2)	15 (11.5)
Left ventricular ejection fraction, mean (SD)	63.2 (5.0)	63.3 (4.3)	62.5 (5.4)
Laboratory test results			
Hemoglobin, mean (SD), g/dL	14.3 (1.3)	14.2 (1.4)	14.1 (1.3)
Hematocrit, mean (SD), %	42.8 (4.0)	42.5 (4.1)	42.0 (4.0)
Platelet count, mean (SD), 10^3^/μL	228.6 (60.0)	220.5 (59.4)	218.1 (51.3)
Serum creatinine, mean (SD), mg/dL	0.7 (0.2)	0.8 (0.2)	0.7 (0.2)
Positive for *Helicobacter pylori*	29 (22.0)	19 (14.4)	28 (21.5)
Positive for fecal occult blood	7 (5.3)	2 (1.5)	4 (3.1)

Abbreviations: BMI, body mass index (calculated as weight in kilograms divided by height in meters squared); DAPT, dual antiplatelet therapy; NSTEMI, non–ST-segment elevation myocardial infarction; PCI, percutaneous coronary intervention.

SI conversion factors: To convert hemoglobin to g/L, multiply by 10.0. To convert hematocrit to proportion of 1.0, multiply by 0.01. To convert platelet count to 10^9^/L, multiply by 1.0. To convert creatinine to μmol/L, multiply by 88.4.

^a^ Unless indicated otherwise, values are presented as the No. (%) of patients. The modified intention-to-treat population included all patients with results from 3 completed magnetically controlled capsule endoscopies of the stomach and the whole small intestine.

### Gastric Injury Progression

Between MCEs at 6 and 12 months, gastric injury occurred in 49 aspirin users (37.1%), 64 clopidogrel users (48.5%), and 69 DAPT users (53.1%) (*P* = .02) (Table 2 and eFigure 2 and eTable 3 in Supplement 2), reflecting a lower rate of gastric injury progression among aspirin users vs DAPT users (RR, 0.70 [95% CI, 0.49-0.99]; *P* = .009). There were no significant differences in rates of gastric injury progression between clopidogrel alone and DAPT (RR, 0.91 [95% CI, 0.67-1.24]; *P* = .46) or between aspirin alone and clopidogrel alone (RR, 0.77 [95% CI, 0.53-1.10]; *P* = .06).

**Table 2.** Gastric and Small-Intestinal Injury Progression From Randomization to 6 and 12 Months

	No./total No. (%) of patients^a^			*P* value			
	Aspirin alone (n = 132)	Clopidogrel alone (n = 132)	DAPT (n = 130)	Aspirin vs clopidogrel vs DAPT	Aspirin vs clopidogrel	Aspirin vs DAPT	Clopidogrel vs DAPT
**Gastric injury**							
Progression	49 (37.1)	64 (48.5)	69 (53.1)	.02	.06	.009	.46
Newly developed	19/36 (52.8)	24/39 (61.5)	22/34 (64.7)	.57	.44	.31	.78
Previous	30/96 (31.2)	40/93 (43.0)	47/96 (49.0)	.04	.09	.01	.41
**Small-intestinal injury**							
Progression	51 (38.6)	65 (49.2)	71 (54.6)	.03	.08	.01	.38
Newly developed	23/46 (50.0)	17/41 (41.5)	24/51 (47.0)	.72	.42	.77	.59
Previous	28/86 (32.6)	48/91 (52.7)	47/79 (59.5)	.001	.007	.001	.38

Abbreviation: DAPT, dual antiplatelet therapy.

^a^ The modified intention-to-treat population included all patients with results from 3 completed magnetically controlled capsule endoscopies of the stomach and the whole small intestine.

### Small-Intestinal Injury Progression

Small-intestinal injury progression between 6 and 12 months occurred in 51 aspirin users (38.6%), 65 clopidogrel users (49.2%), and 71 DAPT users (54.6%) (*P* = .03) (Table 2 and eFigure 3 and eTable 3 in Supplement 2), reflecting a lower rate of small-intestinal injury progression among aspirin users vs DAPT users (RR, 0.71 [95% CI, 0.50-0.99]; *P* = .01). The percentage of patients experiencing small-intestinal injury progression did not significantly differ between clopidogrel vs DAPT users (RR, 0.90 [95% CI, 0.67-1.21]; *P* = .38) or between aspirin vs clopidogrel users (RR, 0.78 [95% CI, 0.55-1.12]; *P* = .08).

### Subgroup Analysis According to Gastric Injury at 6 Months

There were 285 patients (72.3%) with gastric injury after 6 months of DAPT, as detected on the second MCE. Additional gastric injury progression between 6 and 12 months was greatest among patients who continued DAPT (47 of 96 [49.0%]), followed by clopidogrel users (40 of 93 [43.0%]) and aspirin users (30 of 96 [31.2%]; *P* = .04) (Table 2 and eFigure 2 and eTable 3 in Supplement 2), reflecting a lower rate of increased gastric injury among patients who used aspirin alone vs those who continued DAPT (RR, 0.64 [95% CI, 0.40-1.00]; *P* = .01). No significant differences were noted between clopidogrel and DAPT or aspirin and clopidogrel. In patients without gastric injury at 6 months, the incidence of newly developed gastric injury between 6 and 12 months was similar with aspirin alone, clopidogrel alone, and DAPT (19 of 36 [52.8%] vs 24 of 39 [61.5%] vs 22 of 34 [64.7%]; *P* = .57). No significant interaction effect was found (eTable 4 in Supplement 2).

### Subgroup Analysis According to Small-Intestinal Injury at 6 Months

There were 256 patients (65.0%) with small-intestinal injury detected by the second MCE after 6 months of DAPT. Among these patients, 28 of 86 (32.6%) randomized to aspirin alone after 6 months, 48 of 91 (52.7%) randomized to clopidogrel alone, and 47 of 79 (59.5%) randomized to continued DAPT had a further increase in small-intestinal injury between 6 and 12 months (*P* = .001) (Table 2 and eFigure 3 and eTable 3 in Supplement 2), reflecting a lower rate of intestinal injury progression among patients who used aspirin alone vs those who used clopidogrel alone (RR, 0.62 [95% CI, 0.39-0.98]; *P* = .007) or DAPT (RR, 0.55 [95% CI, 0.35-0.86]; *P* = .001). There was no difference in the incidence of increased small-intestinal injury between 6 and 12 months with clopidogrel vs DAPT use (RR, 0.89 [95% CI, 0.63-1.24]; *P* = .38). Among patients without small-intestinal injury after 6 months of DAPT, no significant difference in the incidence of newly developed small-intestinal injury between 6 and 12 months was observed among the 3 groups (23 of 46 [50.0%] vs 17 of 41 [41.5%] vs 24 of 51 [47.0%]; *P* = .72). No significant interaction effect was found (eTable 4 in Supplement 2). To verify the reliability of the results, we performed a sensitivity analysis based on the ITT population, and the results were similar to the mITT analysis (eTable 5 in Supplement 2).

## Discussion

To our knowledge, this study is the first to investigate the rate of gastrointestinal injury progression with different antiplatelet therapies after PCI in patients without high risk of GIB. Following a 6-month DAPT course, 53.1% of patients in this study experienced further gastrointestinal injury progression between 6 and 12 months and 54.6% experienced further small-intestinal injury progression. Aspirin monotherapy had the lowest rate of progressive gastrointestinal injury among the 3 regimens. Clopidogrel alone and DAPT induced more progressive gastrointestinal injury, especially in the subgroup with gastrointestinal injury after the initial 6-month DAPT course. In contrast, the incidence of newly developed gastric or small-intestinal injury between 6 and 12 months did not differ significantly between the 3 antiplatelet regimens in the subgroup of patients without gastrointestinal injury at 6 months.

Patients with clinically silent gastric or small-intestinal injury are at increased risk of developing overt GIB.^10^ The findings of our study suggest that patients without a high risk of GIB treated with continuous antiplatelet therapy may progress to a high-risk phenotype for GIB. Nearly 40% of aspirin users and 50% of clopidogrel or DAPT users developed progressive gastrointestinal injury from 6 to 12 months. Current guidelines do not recommend routine gastrointestinal prophylaxis for patients without high GIB risk, because tiny gastrointestinal mucosal breaks (3-5 mm in diameter) do not necessarily respond to gastrointestinal protective measures.^21,22^ Moreover, esophagogastroduodenoscopy to assess GIB risk is not performed routinely in patients without overt bleeding.^23,24^ Previous studies have not examined serial changes in gastrointestinal tract injury prior to major GIB.^25,26^ The results of our study suggest that routine MCE in patients without a high risk of GIB may identify those with dynamic changes in gastrointestinal tract injury scores during antiplatelet therapy, portending an increased risk of overt GIB. Judicious approaches in such patients may include gastrointestinal prophylaxis or a shortened DAPT course if ischemic risk is low. Conversely, patients tolerating antiplatelet treatment may be good candidates for long-term antiplatelet therapy. Ideally, these strategies must be investigated based on the information provided by MCEs in randomized trials.

In this study, the rate of progressive gastric injury was substantially lower with aspirin alone than with clopidogrel alone or DAPT, as expected.^27,28^ Patients receiving clopidogrel and DAPT had a similar incidence of gastric injury progression. After 6 months of DAPT, patients with gastric injury had consistent outcomes, whereas those without gastric injury had a more uniform response to all 3 antiplatelet regimens. These data demonstrate that clopidogrel may increase the substrate for GIB risk compared with aspirin in the presence of previous mucosal damage. Enteric-coated aspirin may not damage the gastric mucosa. Meanwhile, clopidogrel, an adenosine diphosphate receptor antagonist, may impair gastric ulcer healing by suppressing the release of platelet-derived growth factors.^29,30^ Our study provides confirmatory evidence in humans of the risks of clopidogrel to the gastrointestinal tract, especially in patients with previous mucosal damage perfusing with acidified aspirin. We found similar associations in the response of the small-intestinal mucosa to aspirin, clopidogrel, and DAPT. Additionally, in patients with small-intestinal injury after 6 months of DAPT, clopidogrel monotherapy and DAPT was associated with a higher rate of further small-intestinal injury progression than aspirin. These findings extend the results from prior studies that reported a higher incidence of small-intestinal injury with DAPT than aspirin monotherapy^10,31^ and that clopidogrel exacerbates aspirin-related small-intestinal injury or prevents healing.^32^ However, the mechanism underlying how clopidogrel drives gastrointestinal injury from intact mucosa remains uncertain.

### Limitations

This study has some limitations. First, as a post hoc analysis from the OPT-PEACE trial, it should be considered exploratory and was not specifically powered, especially when comparing individual groups. Second, there was an unavoidable selection bias because our study was based on an Asian population and most enrolled patients were men. Our results require validation in further studies with a broader range of patients. Third, each group contained a comparable proportion of patients who consumed alcohol or had *H. pylori* infection. Future studies are needed to explore the potential influence of these factors on gastrointestinal injury progression. Fourth, 22.0% of participants needed the third MCE. To verify the reliability of the results, we performed a sensitivity analysis based on the ITT population, and the results were similar to the mITT analysis. Fifth, we did not conduct a cost-effectiveness analysis because that requires a separate study considering the specific MCE surveillance plan, the costs of MCEs, and the association between MCE findings and GIB risk. Sixth, this study was performed in patients without high GIB risk, with a randomized treatment period between 6 and 12 months. Although this period is crucial to decision-making for DAPT vs antiplatelet agent monotherapy, especially in patients with high ischemic risk, studies with longer-term follow-up are needed to better explore the associations among antiplatelet therapies, mucosal injury progression, and GIB.

## Conclusions

In this secondary analysis of the OPT-PEACE randomized clinical trial, serial MCEs showed that among patients without high bleeding risk who received a 6-month DAPT course after PCI, aspirin, clopidogrel, and their combination all were associated with gastrointestinal injury progression. Such progression is known to predispose patients to subsequent overt GIB. Use of aspirin alone had the lowest incidence of gastrointestinal injury progression between 6 and 12 months. Conversely, patients receiving clopidogrel monotherapy or DAPT were more likely to experience progressive gastrointestinal injury. Further studies are warranted to determine whether incorporating the findings from routine MCEs into clinical decision-making for the selection and duration of antiplatelet therapy may improve outcomes for patients with ischemic heart disease.

## Notes

Supplement 1.
Trial Protocol

Supplement 2. **eTable 1.** Antiplatelet Medication Use at Baseline and During 12-Month Follow-Up of the Modified Intention-to-Treat Population **eTable 2.** Incidence of Gastric Injury and Small-Intestinal Injury at Baseline of the Modified Intention-to-Treat Population **eTable 3.** Progression of Gastric Injury and Small-Intestinal Injury From Randomization at 6 Months Through 12 Months of the Modified Intention-to-Treat Population **eTable 4.** Interaction Test Between Subgroups and Intervention Groups **eTable 5.** Progression of Gastric Injury and Small-Intestinal Injury From Randomization at 6 Through 12 Months of the Intention-to-Treat Population **eFigure 1.** Magnetically Controlled Capsule Endoscopy System **eFigure 2.** Progression of Gastric Injury From Randomization at 6 Through 12 Months **eFigure 3.** Progression of Small-Intestinal Injury From Randomization at 6 Through 12 Months

Supplement 3.
Data Sharing Statement
