# Error in Byline and Corresponding Author Contact Information

**DOI:** 10.1001/jamanetworkopen.2023.51205

**Published:** 2023-12-21

In the Original Investigation titled “Progression of Gastrointestinal Injury During Antiplatelet Therapy After Percutaneous Coronary Intervention: A Secondary Analysis of the OPT-PEACE Randomized Clinical Trial,”^1^ published November 17, 2023, Ya-Ling Han’s name was misspelled in the byline and the corresponding author contact information and her email address were incorrect. This article has been corrected.
