# Supplementary material for: Progression of Gastrointestinal Injury During Antiplatelet Therapy After Percutaneous Coronary Intervention: A Secondary Analysis of the OPT-PEACE Randomized Clinical Trial
Source: JAMA Netw Open. 2023 Nov 17;6(11):e2343219. doi: 10.1001/jamanetworkopen.2023.43219 (PMC10656648; doi:10.1001/jamanetworkopen.2023.43219)
Supplement: Supplement 1. — Trial Protocol [file jamanetwopen-e2343219-s001.pdf]

This supplement contains the following items:

1. Original protocol, final protocol, summary of changes.
2. Statistical analysis plan

# OPT-PEACE Study

Comparison of mono-versus dual antiPlatelet Therapy during 6-12 months after new generation drug eluting stent implantation for Prevention of gastrointestinal injury Evaluated by Ankon magnetically controlled Capsule Endoscopy: a multicenter, randomized, double-blind, placebo-controlled study

**Sponsor:** General Hospital of Northern Theatre Command

**Version** 1.3

**Contract Research Organization:** ExcellentCRO Co, Ltd, Shenyang, China.

# PROTOCOL SYNOPSIS

| | |
|---|---|
| **Title of Study** | OPT-PEACE Study - Comparison of mono-versus dual antiPlatelet Therapy during 6-12 months after new generation drug eluting stent implantation for Prevention of gastrointestinal injury Evaluated by Ankon magnetically controlled Capsule Endoscopy: a multicenter, randomized, double-blind, placebo-controlled study |
| **Principal Investigator:** | Yaling Han, MD, PhD, FACC<br>Professor of Medicine, Director of Department of Cardiology, General Hospital of Northern Theatre Command and Academician of Chinese Academy engineering |
| **Co-principal Investigators:** | Zhaoshen Li, MD, PhD<br>Professor of Medicine, Director of Department of Gastroenterology, Changhai Hospital, Second Military Medical University/Naval Medical University and Academician of Chinese Academy engineering<br><br>G.W Stone, MD, PhD, FACC<br>Professor of Medicine, Director of Columbia University Medical Center<br><br>Xiaozeng Wang, MD<br>Professor of Medicine, Deputy Director of Department of Cardiology, General Hospital of Northern Theatre Command |
| **Study Centers:** | 26 sites in China |
| **Study Design** | Multicenter, prospective, randomized, double-blinded, placebo-controlled study |
| **Purposes** | The primary study objective is to determine the risks of 12 months of DAPT vs 6 months of DAPT followed by 6 months of aspirin monotherapy or clopidogrel monotherapy on gastrointestinal mucosal injury after DES implantation.<br>The secondary objective is to evaluate the feasibility and safety of AMCE as a method for detecting gastrointestinal mucosal injury and bleeding in patients receiving APT.<br>The exploratory objective is to establish a gastrointestinal mucosal injury scoring system that may identify patients at future risk for clinical gastrointestinal bleeding during long-term APT. |
| **Randomization** | All enrolled patients after PCI firstly received DAPT as a composite of aspirin plus clopidogrel for 6 months, and are then randomly assigned in a 1:1:1 ratio to receive:<br>A: aspirin plus clopidogrel, for an additional 6 months;<br>B: aspirin plus clopidogrel-placebo, for an additional 6 months;<br>C: aspirin-placebo plus clopidogrel plus for an additional 6 months. |

| | |
|---|---|
| **Primary Endpoint** | The incidence of gastric or intestinal mucosal injury occurring within 12 months after enrollment, defined as erosion, ulceration or bleeding detected by either planned AMCE or clinically-driven endoscopy. |
| **Secondary Endpoints** | 1. The incidence and severity of gastric and intestinal mucosal lesions during the first 6 months after study enrollment (prior to randomization);<br>2. The incidence and severity of gastric and intestinal mucosal lesions after randomization (i.e., between 6 months and 12 months after study enrollment);<br>3. The incidence of clinically evident gastrointestinal hemorrhage attributed to the upper GI tract (or of unknown origin) during 6 months after study enrollment (prior to randomization);<br>4. The incidence of clinically evident gastrointestinal hemorrhage attributed to the upper GI tract (or of unknown origin) after randomization (i.e., between 6 months and 12 months after study enrollment);<br>5. The incidence of clinically evident gastrointestinal hemorrhage attributed to the upper GI tract (or of unknown origin) during 12 months after study enrollment;<br>6. Gastrointestinal symptoms (pain, nausea/vomiting, dysphagia, other) during the 12 months after enrollment;<br>7. All bleeding (BARC types 1–5) during the 12 months after enrollment;<br>8. The incidence of target lesion failure (TLF; cardiac death, target-vessel MI, or clinically-driven target lesion revascularization), during the 12 months after enrollment;<br>9. The incidence of net adverse clinical events (NACE, defined as TLF or BARC type 2–5 bleeding) during the 12 months after enrollment;<br>10. The incidence of stent thrombosis (ARC definite, probable, or definite/probable) during the 12 months after enrollment. |
| **Inclusion Criteria** | (All must be present)<br>1. Adult patients with age 18–80 years;<br>2. Presentation with stable angina, or non-ST-segment elevation acute coronary syndrome with GRACE score <140 at admission;<br>3. PCI with implantation of contemporary drug-eluting stent(s) during the present admission;<br>4. Complete revascularization (successful PCI treatment of all epicardial coronary lesions with diameter stenosis ≥70% or intermediate lesions with FFR <0.80);<br>5. Planned DAPT with aspirin and clopidogrel for at least 6 months;<br>6. Agreement to comply with all study procedures;<br>7. Written informed consent provided. |

| **Exclusion Criteria** | (All must be absent)<br>1. Presentation with STEMI, or NSTE-ACS with GRACE score ≥140;<br>2. Left main disease (diameter stenosis >30%);<br>3. Any prior coronary stent implantation during the last year prior to the index procedure;<br>4. Implantation of first-generation drug-eluting stents or bioabsorbable scaffolds during the index procedure;<br>5. Implantation of >4 stents during the index procedure;<br>6. Any prior stent thrombosis;<br>7. Any active gastrointestinal bleeding or ulcers, or prior gastrointestinal bleeding or ulcers within the last 24 months;<br>8. Prior gastrointestinal tract or abdominal surgery other than simple procedures which would not change the gastrointestinal tract anatomy, such as polyp removal, cholecystectomy or appendectomy;<br>9. Contraindications to the AMCE test, including suspected or known gastrointestinal obstruction, stenosis, fistula, diverticula, etc.; presence of gastrointestinal obstruction symptoms such as pain or dysphagia; inoperative conditions or refusal to undergo abdominal surgery if required (because once the capsule could not pass the tract, a surgery may be needed);<br>10. Severe hemorrhoids (phase 3–4 according to guidelines of American Society of Colon and Rectal Surgery);<br>11. LVEF <0.40 on admission by echocardiography;<br>12. Renal dysfunction (eGFR <30 ml/min/1.73m$^2$);<br>13. Active hepatitis or ALT >3 times upper limits of normal at admission;<br>14. Severe hypertension (>180/110 mmHg), or hypertension without control;<br>15. Hemoglobin <100 g/L;<br>16. Platelet count <100 × 10$^9$/L;<br>17. Unable to restrict use of PPI, gastric mucosa protectant or any other antacid agent by rule (details in "restrictions during the study")<br>18. Required use of oral anticoagulation (warfarin or other factor II or factor X inhibitors);<br>19. Inability to take 12-month DAPT for any reason;<br>20. Mandatory use of >6-month DAPT (indicating those who are not suitable to receive aspirin or clopidogrel monotherapy);<br>21. Any comorbidity with estimated survival time <12 months (e.g., progressive cancer, chronic obstructive lung disease, etc.);<br>22. Any contraindication to MRI examination, including implantation of an MRI-incompatible pacemaker, defibrillator, or other ferromagnetic material, etc.;<br>23. Pregnant or plan to be pregnant;<br>24. Any condition that may interfere with any study procedures, such as dementia, immobility, alcohol use, etc.;<br>25. Planned surgery within 1 year;<br>26. Participating in any other clinical trial of an investigational drug or device that has not met its primary endpoint. |
|---|---|

| | |
|---|---|
| **Evaluation of Randomization Eligibility** | Eligibility for randomization will be evaluated in all enrolled patients at 6 months after PCI. At this time patients with any of the following exclusion criteria will be suspended for randomization.<br>1. Withdrawal of informed consent;<br>2. Lost to follow-up at 6 months;<br>3. Any event in the prior 6 months which in the opinion of the investigator results in the patient not being suitable for randomization of antiplatelet agent regimen either because of a) necessity to continue dual antiplatelet therapy (e.g., major adverse cardiovascular or cerebrovascular event within the prior 6 months or need for repeat stenting), or b) inability to continue dual antiplatelet therapy (e.g., bleeding, neoplasm, need for urgent surgery, etc. within the prior 6 months);<br>4. Not presently taking both aspirin and clopidogrel, or any prior temporary discontinuation of aspirin or clopidogrel for ≥5 days;<br>5. Use of proton pump inhibitors or gastric mucosal protectants for more than 12 days, or for more than 4 continuous days in the 6 months prior to randomization;<br>6. Unwillingness or inability to undergo the 6-month AMCE examination or the remainder of the study procedures, including the 12-month AMCE exam. |
| **Statistical Methods** | |
| **Primary Endpoint Analysis** | The primary endpoint analysis will be conducted according to the intention-to-treat (ITT) principle. The main aim of this study is to determine whether the strategy of 6-month DAPT plus 6-month single antiplatelet therapy is superior to 12-month DAPT in terms of the primary endpoint (gastrointestinal injury) after contemporary DES implantation. The null hypothesis ($H_0$) for this analysis is that the incidence of primary endpoint in the experimental group is same as that of the control group, namely $P_0=P_1$. The alternative hypothesis ($H_1$) is that the incidences of primary endpoints in the two groups was not equal, namely $P_0 \neq P_1$, and the superiority test is conducted at the 2-sided significance level of 0.05. This analysis will be repeated in the Per-protocol (PP) population to support the main results. |

| | |
|---|---|
| **Sample Size Determination and Adjustment** | The cumulative incidence of the primary endpoint of gastric or small intestinal mucosal lesions within 12 months is estimated to be 47% in patients who received 12 months of DAPT and 30% in those treated with either aspirin or clopidogrel monotherapy beginning at 6 months after enrollment. With a 2:1 ratio in patients treated with either aspirin or clopidogrel monotherapy (the sum of aspirin monotherapy group and clopidogrel monotherapy group) after 6-month DAPT versus DAPT for 12 months, 384 evaluable patients (256 and 128 respectively) provide 90% power to detect a 17% absolute risk reduction (36% relative risk reduction) with a 2-sided type I error of 0.05. Assuming 20% loss of evaluable primary endpoint outcome assessments due to patient withdrawal, loss to follow-up between 6 and 12 months or suboptimal AMCE visualization of the GI tract at 12months, 480 patients are planned to be randomized. Assuming that an additional 10% of enrolled patients will not be randomized at 6 months because of adverse clinical events, non-compliance with antiplatelet therapy, lost to follow-up or withdrawal, 534 patients is required to be initially planned to be enrolled after baseline screening. Finally, assuming that 10% of patients who undergo a screening AMCE examination will be excluded due to unavailable valid image (or definite ulceration and active bleeding), therefore a total of 593 patients is planned to be consented and undergo the screening AMCE examination. |

**TABLE OF CONTENTS** **PAGE**

TITLE PAGE 1
PROTOCOL SYNOPSIS 2
PROTOCOL SIGNATURE PAGE 10
1. INTRODUCTION 11
1.1 Study Hypothesis 12
1.2 Rationale of Study 12
1.3 Benefit/risk and ethical assessment 13
2. STUDY OBJECTIVES 13
2.1 Primary Objective 13
2.2 Secondary Objective 13
2.3 Exploratory Objective 13
3. STUDY DESIGN AND FLOW CHART 14
4. SUBJECT SELECTION CRITERIA 14
4.1 Inclusion criteria 14
4.2 Exclusion criteria 15
5. STUDY CONDUCT 16
5.1 Restrictions during the study 16
5.2 Screening before enrollment 17
5.3 Subject randomization and initiation of investigational product 17
5.4 Procedures for handling subjects incorrectly enrolled or randomized on investigational product 18
5.5 Blinding and procedures for unblinding the study 18
5.5.1 Methods for ensuring blinding 19
5.5.2 Methods for breaking the blinding in the study 19
5.5.3 Methods for unblinding in the study 19
5.6 Treatments 20
5.6.1 Identity of investigational product 20
5.6.2 Doses and treatment regimens 20
5.6.3 Rationale and procedures of AMCE 21
5.6.4 Labeling 22
5.7 Concomitant and post-study treatment 22
5.8 Treatment compliance 22
5.9 Discontinuation of investigational product 22
5.9.1 Temporary discontinuation from Study Medication 22
5.9.2 Permanent discontinuation from Study Medication due to reasons below: 23
5.10 Withdrawal from study 23
6. COLLECTION OF STUDY VARIABLES 24
6.1 Recording of data 24
6.2 Data collection at enrollment and follow-up 24
6.2.1 Enrollment procedures 26
6.2.2 Face-to face follow-up procedure at 6 months 26
6.2.3 Face-to face follow-up procedure at 12 months (for randomized

subjects) ....27
6.3 Efficacy and safety variables .... 27
7. SAFETY .... 27
7.1 Definition of adverse events .... 27
7.2 Definitions of serious adverse events .... 28
7.3 Recording of adverse events or serious adverse event .... 28
7.3.1 Time period for collection of adverse events ....28
7.3.2 Follow-up of unresolved adverse events ....28
7.3.3 Information to be collected for each AE/SAE ....29
7.3.4 Adverse Events based on signs and symptoms ....29
7.3.5 Adverse Events based on examinations and tests ....30
7.3.6 Disease progression or pre-existing conditions ....30
7.3.7 Reporting of adverse events ....30
8. ETHICAL AND REGULATORY REQUIREMENTS .... 30
8.1 Ethical conduct of the study .... 30
8.2 Ethics and regulatory review .... 31
8.3 Informed consent .... 31
8.4 Changes to the protocol and informed consent form .... 32
8.5 Deviations from protocol .... 32
8.5.1 Compliance to protocol ....32
8.5.2 Procedures for recording, reporting, and analysing protocol deviations ....33
8.6 Audits and inspections .... 33
9. STUDY MANAGEMENT .... 33
9.1 Training .... 33
9.1.1 Training of Monitors ....33
9.1.2 Training of study site research personnel ....33
9.2 Monitoring of the study .... 34
9.3 Study timetable and end of study .... 35
10. DATA MANAGEMENT .... 35
11. EVALUATION AND CALCULATION OF VARIABLES .... 35
11.1 Primary Endpoint .... 35
11.2 Secondary Endpoint .... 36
12. STATISTICAL METHODS AND SAMPLE SIZE DETERMINATION .... 36
12.1 Description of analysis sets .... 37
12.1.1 Efficacy analysis set ....37
12.1.2 Safety analysis set ....37
12.2 Methods of statistical analyses .... 38
12.3 Determination of sample size .... 39
12.4 Clinical event committee .... 39
12.5 Data and safety monitoring board .... 40
13. IMPORTANT MEDICAL PROCEDURES TO BE FOLLOWED BY THE INVESTIGATOR .... 40
13.1 Overdose .... 40

**13.2 Pregnancy** .......... 40
**14. LIST OF REFERENCES** .......... 42
**15. APPENDIX 1- DEFINITION FOR STUDY ENDPOINTS** .......... 43
**15.1 Definition of erosion and ulcer** .......... 43
**15.2 Definition and classification of gastrointestinal (GI) bleeding[6]** .......... 43
**15.3 The classification of internal hemorrhoids according to the guidelines of diagnosis and treatment of hemorrhoids of the American Society of Colon and Rectal Surgeons** .......... 43
**15.4 Bleeding Academic Research Consortium definition of bleeding** .......... 44
**15.5 Major Adverse Cardiovascular and Cerebrovascular Events** .......... 45
**15.5.1 Death** .......... 45
**15.5.2 Myocardial infarction** .......... 45
**15.5.3 Clinically-driven target vessel revascularization** .......... 46
**15.6 Stent thrombosis[8]** .......... 47
**16. APPENDIX 2 - MAGNETICALLY CONTROLLED CAPSULE ENDOSCOPY SCORING SYSTEM** .......... 48
**16.1 The gastric mucosal injury is evaluated through the Lanza score[9]** .......... 48
**16.2 Five-point scoring system for small intestinal mucosal injury[10]** .......... 48
**17. APPENDIX 3 – GASTROINTESTINAL SYMPTOM SCORE** .......... 49
**18. APPENDIX 4 – TREATMENT PRINCIPLES OF GASTROINTESTINAL BLEEDING AND MUCOSAL INJURY** .......... 49
**18.1 Fecal Occult Blood (FOB) Tests** .......... 50
**18.2 The treatment procedure of clinically-driven or AMCE-positive gastrointestinal (GI) bleeding** .......... 51
**18.3 The treatment principle of clinically-driven or AMCE-positive gastrointestinal (GI) bleeding** .......... 51
**18.4 Judgment criteria and treatment rules of severe gastrointestinal mucosal lesions requiring PPI** .......... 53

# PROTOCOL SIGNATURE PAGE

I have read this clinical investigation plan and appendices and agree to adhere to the requirements. I will provide copies of this clinical investigation plan and all pertinent information to the trial personnel under my supervision. I will discuss this material with them and ensure they are fully informed regarding the device and the conduct of the trial.

I will conduct the trial in accordance with the clinical investigation plan, Good Clinical Practice guidelines, the Declaration of Helsinki, EN ISO 14155:2011 (Clinical Investigation of Medical Devices for Human Subjects - Good Clinical Practice), as well as local regulations. I also accept respective revisions to the clinical investigation plan approved by authorized personnel of the ARO and by regulatory authorities.

*Do not copy, distribute, or share this document with others without prior written authorization.*

Investigator name (print):

Investigator name (signature):

Date:

Institution Name (print):

# 1. INTRODUCTION

Antiplatelet therapy (APT) including aspirin and P2Y12 inhibitors has been the cornerstone for prevention of coronary artery disease (CAD), and current guidelines recommend long-term dual antiplatelet therapy (DAPT) with aspirin in combination with one kind of P2Y12 inhibitor after percutaneous coronary intervention (PCI). However, APT may have serious adverse consequences, the most common of which is gastrointestinal mucosal injury with ulceration and bleeding. The frequency of gastrointestinal complications increases with increasing duration of DAPT. Trials in patients treated with contemporary drug-eluting stents (DES) have demonstrated that shortened DAPT regimens reduce the risk of major bleeding[1] with similar ischemic risk[2]. However, whether there is no definite evidence by current studies exploring the difference of gastrointestinal mucosal injuries among different antiplatelet strategies, mainly due to the absence of sensitive, noninvasive and acceptant methods of detecting gastrointestinal injury.

Nowadays, gastroscopy is the most common detection method for gastrointestinal mucosal injury. Although with a good diagnostic accuracy, gastroscopy is an invasive inspection, and have several limitations below in aspect of screening gastrointestinal bleeding: 1) gastroscopy is invasive and is intolerable for patients, therefore only be used to identify the position and reason of bleeding in patients who have experienced major gastrointestinal bleeding, and is lack of early prediction and early warning effect on gastrointestinal injury and bleeding; 2) Because of the fear of major bleeding caused by the mechanical injury of conducting gastroscopy, a large proportion of gastroenterologists usually asked patients to stop antiplatelet therapy for several days before gastroscopy; 3) upper endoscopy can only detect lesions in the stomach and duodenum, as it does not visualize the remainder of the small intestine.

ANKON® magnetically controlled capsule endoscopy (AMCE) is a novel, noninvasive, actively and precisely controlled system which could visualize gastrointestinal injuries in the gamut of esophagus, stomach and small intestine (except colon). The advantages of AMCE includes: 1) Patient acceptance of AMCE is higher than standard endoscopy as the procedure involves only swallowing a small capsule endoscope with non-invasive, painless, convenient and repeatable image observation; 2) Discontinuation of antiplatelet drugs during AMCE is not necessary, as a result of avoiding the risk of stent thrombosis induced by antiplatelet discontinuation; 3) Not only the stomach and duodenum, but the whole small intestine digestive tract can be detected.

Because of the above advantages, this method can be used for the early evaluation of gastrointestinal mucosal lesions associated with bleeding, and can help detect early focal and concealed bleeding, thus playing as an early warning role a guidance for clinical practice. Previous studies have confirmed that the sensitivity and specificity of AMCE for the detection of focal lesions of the gastrointestinal tract are similar compared with standard endoscopy[3,4]. In this study, we plan to use AMCE as the

evaluation method for gastrointestinal mucosal injury and bleeding, and establish a scoring system for gastrointestinal mucosal injury caused by antiplatelet therapy through a randomized controlled design, in order to evaluate the risk of gastrointestinal injury and bleeding in CAD patients receiving different long-term antiplatelet therapy regimens after the implantation of contemporary DES, and provide clinical evidence for guidance of antiplatelet strategy after PCI.

## 1.1 Study Hypothesis

The principal hypothesis of this study is that following 6 months of DAPT, antiplatelet monotherapy with aspirin or clopidogrel between 6 and 12 months after DES implantation is superior to 12 months of DAPT for preventing gastrointestinal injury detected by AMCE.

## 1.2 Rationale of Study

1. Gastrointestinal mucosal injury (represented by erosion, ulcer, or bleeding) is a common complication of long-term antiplatelet therapy after PCI.
2. Trials in patients treated with contemporary DES have demonstrated that shortened DAPT regimens reduce the risk of major bleeding[1] with similar ischemic risk[2]. However, whether there is no definite evidence by current studies exploring the difference of gastrointestinal mucosal injuries among different antiplatelet strategies, mainly because gastroscopy, the most common detection method for gastrointestinal mucosal injury, have considerable limitations including invasive characteristic, requirement for discontinuation of APT and inability of detection of small intestine, so that can hardly be widely used for patients with PCI.
3. ANKON® magnetically controlled capsule endoscopy (AMCE) is a novel, noninvasive, actively and precisely controlled system which could visualize gastrointestinal injuries in the gamut of esophagus, stomach and small intestine (except colon). Patient acceptance of AMCE is higher than standard endoscopy as the procedure involves only swallowing a small capsule endoscope with non-invasive, painless, convenient and repeatable image observation. Discontinuation of antiplatelet drugs during AMCE is not necessary, which is substantially avoid the potential ischemic risk induced by discontinuation.

Therefore, our study first proposes that, 6-month DAPT plus 6-month antiplatelet monotherapy could significantly reduce the risk of gastrointestinal mucosal injury compared with 12-mongth DAPT after implantation of newer-generation DES, and first use AMCE as the method for detection of gastrointestinal mucosal injury among CAD patients with PCI, and establish a score system based on results of AMCE, to evaluate the risk of gastrointestinal injury and bleeding.

## 1.3 Benefit/risk and ethical assessment

The potential benefits that subjects might realize by participating in this randomized trial is a reduction in gastrointestinal mucosal injury for those who are randomly allocated to the experimental arm. Other benefits applicable to all subjects include the free antiplatelet medications after randomization, free inspections including AMCE examination for three times during the whole study period, fecal occult blood (FOB) and blood routine (BT) tests one time per two months, and close monitoring and surveillance for clinical events that will be performed by study personnel during the course of the trial.

The potential risks include an increased rate of thrombotic events for those who are randomized to the antiplatelet monotherapy arm and increased risk for bleeding in subjects randomized to the DAPT arm. However, based on current guidelines, increases of these potential risks are uncertain.

# 2. STUDY OBJECTIVES

## 2.1 Primary Objective

The primary objective of this study is to determine the risks of 12 months of DAPT vs 6 months of DAPT followed by 6 months of aspirin monotherapy or clopidogrel monotherapy on gastrointestinal mucosal injury after DES implantation.

## 2.2 Secondary Objective

The secondary objective is to evaluate the feasibility and safety of AMCE as a method for detecting gastrointestinal mucosal injury and bleeding in patients receiving APT.

## 2.3 Exploratory Objective

The exploratory objective is to establish a gastrointestinal mucosal injury scoring system that may identify patients at future risk for clinical gastrointestinal bleeding during long-term APT.

# 3. STUDY DESIGN AND FLOW CHART

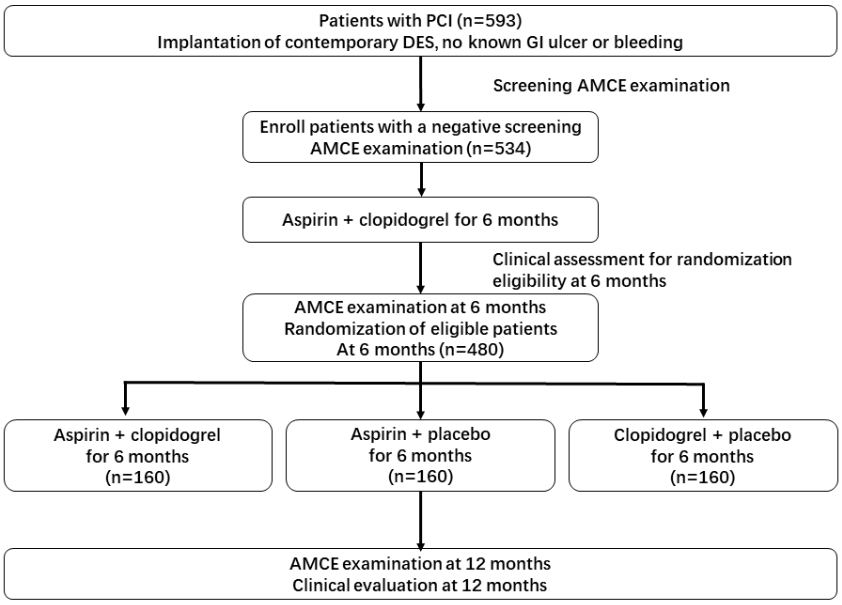


**Study flowchart.** PCI: percutaneous coronary intervention; DES, drug-eluting stent; GI: gastrointestinal; AMCE: ANKON magnetically controlled capsule endoscopy.

# 4. SUBJECT SELECTION CRITERIA

A subject is considered enrolled if all requisite inclusion and none of the exclusion criteria are met and upon provision of informed consent.

## 4.1 Inclusion criteria

1. Adult patients with age 18–80 years;
2. Presentation with stable angina, or non-ST-segment elevation acute coronary syndrome with GRACE score <140 at admission;
3. PCI with implantation of contemporary drug-eluting stent(s) during the present admission (contemporary DESs refer to drug-eluting stents with biodegradable polymer and (or) high biocompatibility and (or) thin cobalt-chromium or platinum-chromium struts. The current major DESs available in China market include: EXCEL (JW Medical System, Weihai, China), Tivoli (Essen Technology, Beijing, China), Endeavor Resolute (Medtronic Inc, Minnesota, USA), FireHawk (MicroPort Medical (Group) Co, Ltd, Shanghai, China), Xience V (Abbott, Abbott Park, Illinois, USA), Xience Prime (Abbott

Laboratories, Abbott Park, Illinois, USA), Promus Element and Synergy (Boston Scientific, Massachusetts, USA), Nano (Lepu Medical Technology CO., LTD, Beijing, China), etc.);

4. Complete revascularization (successful PCI treatment of all epicardial coronary lesions with diameter stenosis ≥70% or intermediate lesions with FFR <0.80);
5. Planned DAPT with aspirin and clopidogrel for at least 6 months;
6. Agreement to comply with all study procedures;
7. Written informed consent provided.

## 4.2 Exclusion criteria

1. Presentation with STEMI, or NSTE-ACS with GRACE score ≥140;
2. Left main disease (diameter stenosis >30%);
3. Any prior coronary stent implantation during the last year prior to the index procedure;
4. Implantation of first-generation drug-eluting stents or bioabsorbable scaffolds during the index procedure;
5. Implantation of >4 stents during the index procedure;
6. Any prior stent thrombosis;
7. Any active gastrointestinal bleeding or ulcers, or prior gastrointestinal bleeding or ulcers within the last 24 months;
8. Prior gastrointestinal tract or abdominal surgery other than simple procedures which would not change the gastrointestinal tract anatomy, such as polyp removal, cholecystectomy or appendectomy;
9. Contraindications to the AMCE test, including suspected or known gastrointestinal obstruction, stenosis, fistula, diverticula, etc.; presence of gastrointestinal obstruction symptoms such as pain or dysphagia; inoperative conditions or refusal to undergo abdominal surgery if required (because once the capsule could not pass the tract, a surgery may be needed);
10. Severe hemorrhoids (phase 3–4 according to guidelines of American Society of Colon and Rectal Surgery);
11. LVEF <0.40 on admission by echocardiography;

12. Renal dysfunction (eGFR <30 ml/min/1.73m$^2$);
13. Active hepatitis or ALT >3 times upper limits of normal at admission;
14. Severe hypertension (>180/110 mmHg), or hypertension without control;
15. Hemoglobin <100 g/L;
16. Platelet count <100 × 10$^9$/L;
17. Unable to restrict use of PPI, gastric mucosa protectant or any other antacid agent by rule (details in "restrictions during the study")
18. Required use of oral anticoagulation (warfarin or other factor II or factor X inhibitors);
19. Inability to take 12-month DAPT for any reason;
20. Mandatory use of >6-month DAPT (indicating those who are not suitable to receive aspirin or clopidogrel monotherapy);
21. Any comorbidity with estimated survival time <12 months (e.g., progressive cancer, chronic obstructive lung disease, etc.);
22. Any contraindication to MRI examination, including implantation of an MRI-incompatible pacemaker, defibrillator, or other ferromagnetic material, etc.;
23. Pregnant or plan to be pregnant;
24. Any condition that may interfere with any study procedures, such as dementia, immobility, alcohol use, etc.;
25. Planned surgery within 1 year;
26. Participating in any other clinical trial of an investigational drug or device that has not met its primary endpoint.

# 5. STUDY CONDUCT

## 5.1 Restrictions during the study

Enrolled subjects should remain on routine aspirin plus clopidogrel for the first 6 months after index PCI, and blinded study drug for the 6 months after randomization (the second 6 months after PCI).

Unless clinically indicated, PPIs or any other type of gastric mucosal protectants

should not be taken by study participants during the course of the trial. Once these drugs are taken, it should not be administered continuously for more than 4 days or a total of more than 12 days before randomization (the first 6 months PCI); it should not be taken for more than 4 days continuously or in total of more than 10 days within 6 months after randomization. Otherwise, any condition that is not in accordance with restrictions above will be considered as a violation of the study protocol, and subjects with this condition will be suspended early from this study.

## 5.2 Screening before enrolment

After provided written consent, all patients (593 patients) meeting the inclusion criteria and not meeting the exclusion criteria will receive an AMCE examination during 30-120 hours after PCI (gut purge is not allowed on the day of PCI) and a *Helicobacter Pylori* (HP) breath test, in order to identify the baseline level of gastrointestinal mucosal lesions and HP infection. Patients undergoing successful screening AMCE examination and with no ulceration or bleeding will be enrolled (at least 534 patients) and treated with open-label aspirin (100 mg/d) plus clopidogrel (75 mg/d) for 6 months. Additionally, blood routine test and FOB test will be performed every 2 months after enrollment.

## 5.3 Subject randomization and initiation of investigational product

A second AMCE examination will be performed to evaluate eligibility for randomization in all enrolled patients at 6 months after PCI. Subjects with any one of items below should not be randomized:

1. Withdrawal of informed consent;
2. Lost to follow-up at 6 months;
3. Any event in the prior 6 months which in the opinion of the investigator results in the patient not being suitable for randomization of antiplatelet agent regimen either because of a) necessity to continue dual antiplatelet therapy (e.g., major adverse cardiovascular or cerebrovascular event within the prior 6 months or need for repeat stenting), or b) inability to continue dual antiplatelet therapy (e.g., bleeding, neoplasm, need for urgent surgery, etc. within the prior 6 months);
4. Not presently taking both aspirin and clopidogrel, or any prior temporary discontinuation of aspirin or clopidogrel for ≥5 days;
5. Proton pump inhibitors or any other kind of gastric mucosal protectant agents may not be used after study enrollment unless a clear clinical indication has developed (e.g., new gastrointestinal bleeding or ulcer disease). After randomization, gastric mucosa protectants may not be taken for more than 4

days continuously or in total for ≥10 days without a clear clinical indication;

6. Unwillingness or inability to undergo the 6-month AMCE examination or the remainder of the study procedures, including the 12-month AMCE exam.

Patients successfully completing the 6-month follow-up AMCE exam and with no ulceration or bleeding (approximately 480 patients) are then randomly assigned in a 1:1:1 ratio to receive three antiplatelet strategies below: Group A, the standard DAPT (160 patients, aspirin plus clopidogrel); Group B, aspirin monotherapy (160 patients, aspirin plus clopidogrel-placebo); and Group C, clopidogrel monotherapy (160 patients, clopidogrel plus aspirin-placebo) for an additional 6 months in a double blinded manner. A third AMCE examination will be done at 12 months after enrollment.

After randomization, patients should also undergo blood routine test and FOB test every 2 months. Moreover, the first 102 enrolled patients (34 per group) from the site of PI (General Hospital of Northern Theatre Command) will undergo platelet function testing, including adenosine diphosphate-induced platelet aggregation by light transmission aggregometry and VerifyNow aspirin and P2Y12 testing assessment.

Patients who are ineligible for randomization will not receive any more study drugs, and subsequent antiplatelet therapy will be administered at their attending physicians' discretion in accordance with the local standard of care. All enrolled patients, irrespective of eligibility for randomization, should be followed up by telephone until 12 months.

## 5.4 Procedures for handling subjects incorrectly enrolled or randomized on investigational product

Subjects who are incorrectly enrolled will be immediately withdrawn from the study. The subject and the treating physician will be notified. If the subject has already been randomized, the study drug will be discontinued and further treatment will be per standard of care.

## 5.5 Blinding and procedures for unblinding the study

This study has a double-blind design with aspirin, clopidogrel and their corresponding placebos. Neither the subjects, study site research personnel, or treating physicians involved in the treatment or clinical evaluation of the subjects will be aware of treatments received. There will be an independent data safety monitoring board (DSMB) to monitor the data on a periodic basis.

## 5.5.1 Methods for ensuring blinding

This study has a double-blind design with aspirin, clopidogrel, and matching placebos. Shenyang Yu Kang Pharmaceutical Technology Co., Ltd, a statistical organization independent of the study, used SAS software to generate random codes for the corresponding groups (primary blind codes), and the study drug regimen corresponding to the group (secondary blind codes), and import the two levels of blind codes (including primary and secondary blind codes) via the Taimei Medical Randomization and Drug Management System (eBalance). Randomization is stratified according to site and will be performed in fixed block. The active tablets and the respective placebo tablets will be identical in size, color, smell, and taste. The patients, site personnel, sponsor personnel, persons performing the assessments, and data analysts will remain blinded to the identity of the treatment from the time of randomization until completing the statistical analysis. There will be an independent data safety monitoring board (DSMB) to monitor the data on a periodic basis.

### 5.5.2 Methods for breaking the blinding in the study

In the event of a medical emergency, in which knowledge of the investigational drug is critical to the subject’s medical management, the blind for that subject may be broken by the treating physician. Before breaking the blinding, the consent of the research leader of the participating research site should be obtained and the relevant researchers and monitors should be informed. At the same time, the events leading to break the blinding and the blind code information should be recorded in detail. After breaking the blinding, the subject will not receive any study drug, and the treated physician can take corresponding bail-out measures according to local standard of care. The study drugs cannot be resumed even if the condition of subject is stable, while researchers still need to conduct clinical follow-up for 12 months for the subject with broken blinding, and AMCE examination is still required. Researchers should withdraw the study drugs from the patients whose blinding was broken.

### 5.5.3 Methods for unblinding in the study

Unblinding will be conducted after the whole study procedure. Because of the randomized, double-blinded, placebo-controlled study design, a two-level unblinding (first and second unblinding) is prespecified. The whole procedure of unblinding is organized by Data and Safety Monitoring Boards (DSMB), and collectively witnessed by representatives of research sites, contract research organization (CRO) and statistical organization. The unblinding should not be

performed until completing the statistical analysis.

The first unblinding: Only the definite group (such as group A, group B, and group C) of each subject is unblinded, but the study drug corresponding to each group is not clear yet. At this time, the information of study drug will be filed by statisticians for statistical analysis.

Second unblinding: After the statistical analysis is finished, a statistical report will be generated. The primary investigator will perform the second unblinding when publishing statistical results, and simultaneously announce the study drug of each group, then combine the blind codes to generate the final statistical results.

## 5.6 Treatments

### 5.6.1 Identity of investigational product

In this protocol, the investigational product is aspirin 100 mg tablets and matching aspirin-placebo tablets, and clopidogrel 75mg tablets and matching clopidogrel-placebo tablets.

**Table 1 Identity of Investigational Product**

| Study drug | The form and dosage of drug | Manufacturer |
|---|---|---|
| Bayaspirin (aspirin) | 100mg tablet | Bayer |
| Aspirin-placebo | tablet | Guangzhou Boji Medical Biotechnological co., Ltd. |
| Plavix (clopidogrel) | 75mg tablet | Sanofi-Aventis |
| Clopidogrel-placebo | tablet | Guangzhou Boji Medical Biotechnological co., Ltd. |

### 5.6.2 Doses and treatment regimens

After enrollment, patients will routinely administer study drugs (including open-label aspirin at a dose of 100mg swallowed with 100ml warm water 30 minutes daily before breakfast, plus open-label clopidogrel at a dose of 75mg daily) until the face-to-face study visit at 6 months. At the face-to-face study visit, randomized participants will be allocated study drugs (including: Group A, blinded-label aspirin at a dose of 100mg daily, plus blinded-label clopidogrel at dose of 75mg daily;

Group B, blinded-label aspirin at a dose of 100mg daily, blinded-label clopidogrel-placebo at dose of 75mg daily; Group C, blinded-label aspirin-placebo at a dose of 100mg daily, plus blinded-label clopidogrel at dose of 75mg daily) and should be used regularly until the end of study.

### 5.6.3 Rationale and procedures of AMCE

ANKON® magnetically controlled capsule endoscopy system (AMCE), which is provided by ANKON Medical Technologies (Shanghai, China) and ANKON Photoelectric Technology Co., Ltd (Wuhan, China, ANKON Enterprise), is composed of a magnetic navigation control system, a portable data recorder and a capsule position detector. The capsule endoscope (known as an endoscopic robot) has a length of 27 mm and a diameter of 11 mm and contains a permanent magnet. It provides a 140° viewing angle, a 30 mm depth of field and operates for at least 10 hours after ingestion. The activated capsule is swallowed into the digestive tract and continually records the condition of digestive tract mucosa. The dimensions of any visualized lesions are measured by the ANKON ESNavi software. After reaching the gastric cavity, the endoscopic capsule is navigated by the external magnetic control system to visualize all aspects of the stomach (the cardia, fundus, angulus, antrum, and pylorus). The controller allows movements of 2 mm and changes in viewing angle of 3°. AMCE is the first actively and accurately capsule gastroscope robot that could actively and precisely provide the whole gastric examination. Multicenter, large-scale randomized studies have demonstrated that the accuracy and specificity of AMCE system was high consistent with standard gastroscopy[3,4].

Preparation before AMCE examination: Patients fasted on food, colored liquid or syrup from 8 pm the day before AMCE examination. Gut purge is shall be done 6-8 hours begore examination. On the following morning the subject is administered 10 ml of simethicone (Menarini Group, Florence, Italy) as a defoaming agent to clean the stomach cavity 40 minutes before the examination, and drinks water (500-1000 ml) until feeling stomach fullness. During examination, if the gastric cavity is not filled with sufficient liquid to enable navigation of the capsule, the subject will drink additional water.

After the whole stomach is examined, subjects continue to wear the portable recorder for visualization of the duodenum and small intestine. After examination of the whole stomach, subjects could leave the hospital but should continue to wear the portable recorder for examination of the small intestine. that Subjects should return to the hospital as soon as possible once the instrument suggests that the whole examination is finished, and are subsequently followed up for up to 2 weeks to determine if the capsule is ultimately excreted or adverse events (AEs) occur. Subjects should record the time when the capsule is discharged with defecation. The capsule can be abandoned after elimination without recycling. Magnetic resonance imaging is prohibited before the confirmation of capsule excretion. If the capsule is not found to be excreted within 2 weeks after examination, the subject returns to the

hospital for detection of the capsule by a position detector or abdominal x-ray to confirm whether the capsule is still in the body. If it is, endoscopy may be performed to remove the capsule.

#### 5.6.4 Labeling

Each aspirin, clopidogrel or placebo bottle will be labeled in black ink. On this label the protocol number, blinded batch number, container number, blinded drug name, tablet quantity, storage conditions, directions for use and route of administration will be indicated.

At the study site, the investigational product should be stored in a secure area according to local regulations. It is the responsibility of the investigator to ensure that the investigational product is only dispensed to study subjects. The investigational product must be dispensed only from official study sites by authorized personnel according to local regulations.

The storage conditions for the subjects to follow will be included in the labeling on the bottles.

## 5.7 Concomitant and post-study treatment

There are no protocol specific concomitant treatments. After study completion, the physician will determine the ongoing medical treatment for each subject. These medications are open-label, and are available on their local sites (no more available for free).

## 5.8 Treatment compliance

Study drug compliance will be assessed using manual pill count at the 12-month in-person follow-up visit. Reconciliation between the quantity of shipped study drugs versus the allocation of study drugs via EDC and versus distribution of study drug to subject will be monitored and reconciled by investigators and trial monitors.

## 5.9 Discontinuation of study medication

### 5.9.1 Temporary discontinuation from study medication

In the event such as surgery or other invasive procedures that a subject is temporarily discontinued from study drugs or need a replacement of the blinded-label study drugs to open-label antiplatelet drugs <7 days according to local standard of care, subjects should be considered, where possible, for eligibility to resume blinded-label study drug under the judgement of treating physicians.

Details on the date, duration and cause of each temporary discontinuation of study drugs should be recorded in full in the appropriate section of the EDC.

### 5.9.2 Permanent discontinuation from study medication due to reasons below:

- Patient decision. The patient is at any time free to discontinue treatment, without prejudice to further treatment.
- Investigator's decision:

  1) Incorrectly enrolled patient in whom the inclusion/exclusion criteria violation would put the patient at undue risk.

  2) Investigators judge that due to adverse events related to any kind of study drug, continuous administration of this study drug would put the patient at undue risk.

Details on the date, duration and cause of each permanent discontinuation of study drugs should be fully recorded into the appropriate section of the EDC. When any of the study drugs are permanently discontinued, further antiplatelet therapy should be given to the subject according to attending physician's discretion. Any remaining study drugs should be returned to the local research site at the next scheduled follow-up visit. Although these subjects are no longer receiving any study drug, they should also be followed up until 12 months. Patients who permanently discontinue study drugs after randomization are still required to undergo AMCE examination, and investigators are required to withdraw study drugs from patients after discontinuation. Patients who permanently discontinue study drugs before randomization only needed to be followed up by telephone at12 months.

## 5.10 Withdrawal from study

Each enrolled subject shall remain in the trial until completion of the required follow-up period. However, a subject's participation in any clinical trial is voluntary and the subject has the right to withdraw at any time without penalty or loss of benefit. Conceivable reasons for discontinuation may include, but not be limited to, the following:

- Subject voluntary withdrawal
- Subject withdrawal by physician as clinically indicated
- Subject lost-to follow-up

The reason for subject discontinuation must be documented on the CRF and source documents. The principal investigators must also report all subject discontinuations to their Ethical Committee as defined by their sites' procedure. All data from

evaluations and treatments performed prior to the withdrawal should be documented on the CRFs. Source documents that pre-date the withdrawal should be submitted as required by the protocol. No data that post-dates the withdrawal will be collected.

Once a subject has withdrawn from the trial, no further follow-up contact will be performed. However, vital status may be obtained from public records. Medical therapy after stopping the study will be as prescribed by the subject's physician.

# 6. COLLECTION OF STUDY VARIABLES

## 6.1 Recording of data

The Investigator is responsible for maintaining complete and accurate documentation of the trial including but not limited to medical records, trial progress records, laboratory results, case report forms, signed informed consent forms, investigational product accountability records, correspondence with the EC or trial monitors, adverse event reports, and information regarding subject discontinuations.

The Investigator is required to maintain information in the subject's medical records which documents and corroborates data entered in the case report forms. As a minimum the subject record should contain:

- Medical history/physical exam documenting that subject meets inclusion/exclusion criteria
- Documentation of subject's consent and subject ID number in the trial
- Dated and signed notes from each subject visit
- Adverse events reported and their resolution or lack thereof including supporting documents such as hospital records, discharge summaries, catheterization reports, ECGs, etc.
- Record of protocol required medications during the trial
- Record of the subject's condition upon completion of or withdrawal from the trial

## 6.2 Data collection at enrollment and follow-up

Data collection commences after the subject has provided informed consent. Data collection including subject demographic information, laboratory tests, and procedural data, AMCE examination data as well as follow-up visits or telephone contacts will be conducted by an Investigator or site coordinator who has been trained on the protocol

and CRFs.

Data required for analysis will be obtained as outlined in Table 2.

**Table 2 schedule of data collection**

| **Study procedures** | **Screening for Enrollment**<br>**After PCI** | **Standard DAPT and randomization**<br>**Until 6 months after PCI** | **After randomization**<br>**Until 12 months after PCI** |
|---|---|---|---|
| Follow-Up | Visit 1 | Visit 2 | Visit 3 |
| Window Period | 0-7 days | ±7 days | ±14 days |
| Eligibility Criteria | X | | |
| Patient Informed Consent | X | | |
| Withdrawal | X | X | X |
| Medical History/ Demographics | X | | |
| Previous Medical History | X | | |
| Vital Signs | X | X | X |
| Height and Weight | X | | |
| Physical Examination | X | | |
| Cardiac Markers (including TnT or TNI/CK/CK-MB) | X | | |
| Blood Routine Test[1] (RBC/WBC/HGB/PLT) | X | X | X |
| Coagulation Function Test (PT/APTT/FIB) | X | | |
| Fecal Occult Blood Test[1] | X | X | X |
| Platelet Aggregation Test[2] (light transmission aggregometry and VerifyNow) | X | X | X |
| HP Breath Test | X | | |
| 12-Leads ECG | X | | |
| AMCE Examination | X | X | X |
| Drug Distribution/Recycling | X | X | X |
| Concomitant Medications | X | X | X |
| Adverse Events | X | X | X |

1: one time per two months; 2: 34 patients per group in the principal research site

6-month visit: Each enrolled subject will be followed with an in-clinic face-to-face visit at 6 months after enrollment. Screening should be conducted before the 6-month face-to-face visit, subjects who are not eligible for randomization will not be required to complete the 6-month face-to-face visit.

12-month visit: Randomized subjects will return for the final in-clinic additional in-clinic face-to-face visits at 12 months after enrollment. Vital status of subjects who

did not meet eligibility for randomization can be obtained from medical records or public records at 12 months after enrollment.

### 6.2.1 Enrollment procedures

For patients who meet all inclusion criteria and do not meet any exclusion criteria, the investigator or designee will:

- Obtain written informed consent
- Obtain a complete demographic data, medical history, physical inspection information (including blood pressure, pulse, height, weight and ECG), and lab values including hemoglobin and creatinine
- Conduct the initial AMCE examination to determine baseline characteristics of gastrointestinal mucosa
- Review concomitant medications taken within the last 30 days
- Instruct the subject to notify the investigator of any occurrence of adverse events
- Schedule next clinic visit at 6 months, and instruct patients to administer DAPT (aspirin plus clopidogrel) during the first 6 months after PCI

### 6.2.2 Face-to face follow-up procedure at 6 months

- Assess for adverse events may include, but not be limited to MI, stroke, revascularization, bleeding and ST
- Review concomitant medications with subject
- Counsel subject about importance of study drug compliance
- Assess for randomization eligibility – If applicable:
    - Randomize subjects via the Taimei Medical Randomization and Drug Management System (eBalance), and obtain container/kit number
    - Conduct the second AMCE examination to observe gastrointestinal mucosal lesions
    - Dispense study medications for next 6 months
    - Schedule next clinic visit at 12 months (for randomized subjects)
- Instruct the subject and/or caregiver to notify the investigator of any occurrence of adverse events

- Instruct the subject to return study drugs at next time of in-clinic face-to-face visit

### 6.2.3 Face-to face follow-up procedure at 12 months (for randomized subjects)

- Assess for adverse events may include, but not be limited to MI, stroke, revascularization, bleeding and ST
- Conduct the third AMCE examination to observe gastrointestinal mucosal lesions
- Review concomitant medications with subject
- Recycle and count all of remaining study drugs

## 6.3 Efficacy and safety variables

The study site research personnel will collect data from the subjects during the follow-up contact for identification of the efficacy and safety variables, defined as the first occurrence of gastrointestinal mucosal injury (including erosion, ulceration and bleeding) detected by AMCE examination (efficacy variable), MACCE (including cardiac death, target lesion MI, ischemic stroke, and clinically-driven target lesion revascularization (safety variables), and GI related symptoms or signs such as abdominal distension, nausea, vomiting, or capsule retention (safety variables).

# 7. SAFETY

The Principal Investigator at each participating study site is responsible for ensuring that all staff involved in the study is familiar with the content of this section.

## 7.1 Definition of adverse events

An adverse event is the development of an undesirable medical condition or the deterioration of a pre-existing medical condition following or during exposure to a pharmaceutical product, whether or not considered causally related to the product. An undesirable medical condition can be symptoms (e.g., nausea, chest pain), signs (e.g., tachycardia, enlarged liver) or the abnormal results of an investigation (e.g., laboratory findings, ECG). In clinical studies, an adverse event (AE) can include an undesirable medical condition occurring at any time, including run-in or washout periods, even if no study treatment has been administered. The term AE is used to include both serious and non-serious AEs.

## 7.2 Definitions of serious adverse events

A serious adverse event is an AE occurring during any study phase (i.e., run-in, treatment, wash-out, follow-up), that fulfills one or more of the following criteria:

- Results in death
- Is immediately life-threatening
- Requires in-patient hospitalization or prolongation of existing hospitalization
- Results in persistent or significant disability/incapacity or substantial disruption of the ability to conduct normal life functions
- Results in a congenital abnormality or birth defect
- Is an important medical event that may jeopardize the subject or may require medical intervention to prevent one of the outcomes listed above.

The severity and causality of SAEs (their relationship to all study treatments and/or procedures) will be assessed by the investigator(s) and reported to the EC.

**Definition of suspected unexpected serious adverse reactions (SUSARs)**

A SUSAR is a Suspected Unexpected Serious Adverse Reaction. To qualify as a SUSAR, the event shall be:

- Serious adverse event
- Adverse reactions to study drugs
- Unexpected reaction – defined as the adverse reaction not regarded as a potential risk or an adverse drug reaction by study protocol, informed written consent or literature.

## 7.3 Recording of adverse events or serious adverse event

### 7.3.1 Time period for collection of adverse events

All AEs related to cardiovascular, cerebrovascular and gastrointestinal aspects and all SAEs should be collected from the time the subject signs the informed consent through study exit.

### 7.3.2 Follow-up of unresolved adverse events

For ALL SAEs the subject’s course must be monitored until the event has subsided or,

in a case of permanent impairment, until the event stabilizes and the overall clinical outcome has been ascertained.

### 7.3.3 Information to be collected for each AE/SAE

- Description of AE/SAE
- The dates when the AE/SAE started and stopped
- Whether the AE/SAE is serious or not
- Time of reporting SAE
- Investigator causality rating against the investigational product
- Action taken with regard to investigational product
- Whether the AE/SAE caused subject's withdrawal from study
- Outcome

**In addition, the following variables will be collected for SAEs:**

- Date the study site research personnel became aware of SAE
- Date when decision was made for meeting SAE criteria
- Criteria met leading to classification as SAE
- Date of hospitalization (if applicable)
- Date of discharge (if applicable)
- Date of death (if applicable)
- Probable cause of death (if applicable)
- Autopsy performed (if applicable)

It is important to distinguish between serious and severe AEs. Severity is a measure of intensity whereas seriousness is defined by the criteria in Section 7.2. An AE of severe intensity need not necessarily be considered serious. For example, nausea that persists for several hours may be considered severe nausea, but not an SAE. On the other hand, a stroke that results in only a limited degree of disability may be considered a mild stroke but would be an SAE.

### 7.3.4 Adverse Events based on signs and symptoms

When collecting AEs, the recording of diagnoses (when possible) is preferred to

recording a list of signs and symptoms. However, if a diagnosis is known and there are other signs or symptoms that are not generally part of the diagnosis, the diagnosis and each sign or symptom will be recorded separately.

#### 7.3.5 Adverse Events based on examinations and tests

Deterioration as compared to baseline in laboratory values or vital signs should therefore only be reported as AEs if they fulfill any of the SAE criteria or are the reason for discontinuation of treatment with the investigational product. If deterioration in a laboratory value/vital sign is associated with clinical signs and symptoms, the sign or symptom will be reported as an AE and the associated laboratory result/vital sign will be considered as additional information. Wherever possible the reporting investigator uses the clinical, rather than the laboratory term (e.g., anemia versus low hemoglobin value). In the absence of clinical signs or symptoms, clinically relevant deteriorations in nonmandated parameters should be reported as AE(s). Any new or aggravated clinically relevant abnormal medical finding at a physical examination as compared with the baseline assessment will be reported as an AE.

#### 7.3.6 Disease progression or pre-existing conditions

Disease progression can be considered as a worsening of a subject's condition attributable to the disease for which the investigational product is being studied. It may be an increase in the severity of the disease under study and/or increases in the symptoms of the disease. For example, persistent angina should be considered as disease progression and not an AE. Planned hospitalization for a pre-existing condition without serious deterioration in health, is not considered a serious adverse event.

#### 7.3.7 Reporting of adverse events

AE should be reported to the EC of the participating research site for record within 2 working days after detection by the relevant personnel of the research site. SAE should be immediately reported to EC of this site, the sponsor, and the national and provincial Food and Drug Administration within 24 hours after detection by the relevant personnel of the research site.

# 8. ETHICAL AND REGULATORY REQUIREMENTS

## 8.1 Ethical conduct of the study

The trial will be conducted in compliance with the protocol, Good Clinical Practice

guidelines, and World Medical Association Declaration of Helsinki: Ethical Principles for Medical Research Involving Human Subjects as well as local regulations, and applicable regional regulatory requirements.

The clinical investigation shall not begin until the required approvals/favorable opinions from the respective regulatory authority and ethics committee have been obtained. Any additional requirements imposed by the respective regulatory authority and/or ethics committee will also be followed, where specified.

## 8.2 Ethics and regulatory review

The principal investigator in each study site shall obtain the approval of the protocol, informed consent and other trial related documents from the EC before participating in the study.

In accordance with the investigational site EC requirements, the Investigator will:

1. obtain written EC approval at predetermined time points to continue the trial;
2. submit any amendments to the protocol as well as associated informed consent form changes and obtain written EC approval obtained prior to implementation.

## 8.3 Informed consent

All subjects must provide written informed consent in accordance with the site's EC, using an EC-approved informed consent form. All subjects are to be fully informed and trial conduct must be in accordance to the World Medical Association Declaration of Helsinki: Ethical Principles for Medical Research Involving Human Subjects.

Protocol-specific procedures or alterations of patient care must not be performed until the prospective subject has provided a signed informed consent. The informed consent will be in the prospective subject's native language and will contain non-technical language to describe the investigational procedures. The informed consent form should also include a clause that ensures important new information will be provided to the subject throughout the clinical investigation.

After a review of the prospective subject's medical records to determine general eligibility, the investigator or authorized designee who has been trained on the protocol, will approach the prospective subject to explain the purpose and scope of the clinical trial, prospective risks, and benefits of participation. The prospective subject must be given the opportunity to ask questions about the trial and must be given sufficient time to decide to participate in the trial or not. Additional information requested by the prospective subject should be provided. Any coercion or undue improper influence on the prospective subject is to be avoided.

If the prospective subject agrees to participate, the informed consent form must be signed and personally dated by the prospective subject. The investigator or an authorized member of the research team who has witnessed the prospective subject's signature must also sign and date the informed consent, prior to enrollment of the prospective subject. A copy of the completed informed consent form must be provided to the subject. Local EC regulations regarding obtaining informed consent must be followed.

The subject's medical record should have a notation regarding the signing of the informed consent. The subject is to be made aware that their participation in the trial is voluntary, their legal rights will not be waived, and that they may withdraw from the trial at any time, without giving specific reason for doing so. The subject must also be informed that withdrawal from the trial will not affect their future treatment. The investigator is responsible for the achievement of written consent from the prospective subject before they are included in the trial. All subjects must provide informed consent in accordance with the local EC requirements, using an EC-approved informed consent form.

## 8.4 Changes to the protocol and informed consent form

If the protocol or informed consent form (ICF) needs an amendment, the principal research site is required to submit such amendment to the Regulatory Agencies and/or other regulating body in each participating country for approval. Approved protocol or ICF amendments will be provided to the investigators by the principal research site prior to implementing the amendment.

For administrative changes, the principal investigator is responsible for notifying the EC.

## 8.5 Deviations from protocol

### 8.5.1 Compliance to protocol

No investigative procedures other than those defined in this clinical investigational plan will be undertaken on the enrolled subjects without the written agreement of the EC and the principal research site. It is the Investigator's responsibility to ensure that there are no deviations from the clinical investigational plan and full compliance with all established procedures of the EC is maintained. The Investigator will not deviate from the clinical investigational plan for any reason except in cases of medical emergencies, when the deviation is necessary to protect the life or physical well-being of the subject.

### 8.5.2 Procedures for recording, reporting, and analysing protocol deviations

A deviation is an instance(s) of failure to follow, intentionally or unintentionally, the requirements of the protocol. All deviations must be reported to the principal research site. The occurrence of clinical investigational plan deviations will be monitored by the designee of participating research sites and trial monitors. It is the Investigators' responsibility to inform their EC of clinical investigational plan deviations in accordance with their specific EC reporting policies and procedures. In the event that an investigative site does not comply with the Investigator Agreement or clinical investigational plan, the principal research site will notify the investigator of the site's non-compliance.

## 8.6 Audits and inspections

In the event that an investigator is contacted by a Regulatory Agency in relation to this trial, the investigator will notify the site immediately. The investigator and study site research personnel must be available to respond to reasonable requests and inspection queries made during the inspection process. The investigator must provide the principal research site with copies of all correspondence that may affect the review of this trial. The principal research site will provide any needed assistance in response to regulatory inspections. For revision about management and safety of subject, it is the principal investigator's responsibility to obtain the approved protocol or ICF amendments from EC.

The EC's approval of the study protocol or ICF amendment above shall be written documented before implementation.

# 9. STUDY MANAGEMENT

## 9.1 Training

### 9.1.1 Training of Monitors

The monitors or designee will be trained to the protocol, randomization instructions, electronic case report forms, and study drug usage. The principal research site is responsible for the training.

### 9.1.2 Training of study site research personnel

Participating investigators and study site research personnel will be trained during site initiation visits collectively conducted by the representatives designed by The General

Hospital of Northern Theatre Command, the Department of Gastroenterology of Changhai Hospital and the ANKON Medical Technologies (Shanghai, China). All training must be documented and must include or reference the revision of materials used for training, who was trained, the trainer and date of training. Original training records should be maintained at the site in the Regulatory Binder and copies should be dispensed to the principal research site.

Site initiation/training involves a didactic session whereby the protocol, including screening procedures, clinical follow-up procedures, and study drug procedures are reviewed in detail along with investigator responsibilities.

## 9.2 Monitoring of the study

The trial monitors will monitor the trial over its duration according to the prespecified monitoring plan. The trial monitor will contact each site at appropriate intervals to review investigational data for accuracy and completeness and ensure compliance with the clinical investigation plan. The trial monitor may request all documents and required records that are maintained by the Investigator/Site, including medical records (office, clinic, or hospital) for the subjects in this trial. Source documentation must be available to substantiate proper informed consent procedures, adherence to protocol procedures, adequate reporting and follow-up of adverse events, accuracy of data collected on case report forms, and study drug information. The Investigator and/or study site research personnel will be available for monitoring contact. If a site visit is required, it is expected that the Investigator/Site will provide the trial monitor with a suitable working environment for review of study-related documents.

### Source data

The investigator is responsible for maintaining complete and accurate documentation of the trial including but not limited to medical records, trial progress records, laboratory results, case report forms, signed informed consent forms, study drug accountability records, correspondence with the EC, the trial monitors, and the principal research site, as well as adverse event reports and information regarding subject discontinuations.

The investigator is required to maintain information in the subject’s medical records which documents and corroborates data entered in the case report forms. The investigator and the associated institution will permit direct access to source data and documents for study-related monitoring, audits, EC review, and regulatory inspections.

Subjects providing informed consent agree to allow the monitor or designee access and copying rights to pertinent information in their medical records concerning their participation in this trial. The investigator will obtain, as part of the informed consent, permission for trial monitors or regulatory authorities to review, in confidence, any

records identifying the subjects in this trial. This information may be shared with regulatory agencies; however, the monitor undertakes not to otherwise release the patient's personal and private information.

### 9.3 Study timetable and end of study

The trial is estimated to commence May 2017. The last subject follow-up at 12 months post-procedure is expected to occur in May 2019. The total expected duration of the trial is 24 months.

# 10. DATA MANAGEMENT

A computerized data entry and management system will be developed by Taimei Medical Randomization and Drug Management System. A closed and password protected data entry system has been designed to ensure that only the responsible data entry person and the Data Management site supervisor can enter and/or edit data and this can be done only by using the programs and/or utilities available on the menu system. An audit trail will be created by date/time and user stamping. Range checks, review screens, and various error trapping routines are built into the system as quality control procedures. All possible relevant information on the forms is pre-coded. The statistical analysis of data on this study will be completed by an independent statistical organization, Shenyang Yu Kang Pharmaceutical Technology Co., Ltd.

# 11. EVALUATION AND CALCULATION OF VARIABLES

## 11.1 Primary Endpoint

The primary endpoint of this study is the gastric and small intestinal mucosal injury (a composite of erosion, ulceration or bleeding) occurring within 12 months after enrollment.

Situations below can be regarded as valid primary endpoint results, including:

- Successful completion of the AMCE examination at 6 months after randomization (i.e., 12 months after enrollment), regardless of the results of examination;
- AMCE or gastroscopy examination driven by the occurrence of gastrointestinal bleeding at any time during the whole study period observes

new-onset gastric or small intestinal mucosal lesions.

## 11.2 Secondary Endpoint

1. The incidence and severity of gastric and intestinal mucosal lesions during the first 6 months after study enrollment (prior to randomization);
2. The incidence and severity of gastric and intestinal mucosal lesions after randomization (i.e., between 6 months and 12 months after study enrollment);
3. The incidence of clinically evident gastrointestinal hemorrhage attributed to the upper GI tract (or of unknown origin) during 6 months after study enrollment (prior to randomization);
4. The incidence of clinically evident gastrointestinal hemorrhage attributed to the upper GI tract (or of unknown origin) after randomization (i.e., between 6 months and 12 months after study enrollment);
5. The incidence of clinically evident gastrointestinal hemorrhage attributed to the upper GI tract (or of unknown origin) during 12 months after study enrollment;
6. Gastrointestinal symptoms (pain, nausea/vomiting, dysphagia, other) during the 12 months after enrollment;
7. All bleeding (BARC types 1–5) during the 12 months after enrollment;
8. The incidence of target lesion failure (TLF; cardiac death, target-vessel MI, or clinically-driven target lesion revascularization), during the 12 months after enrollment;
9. The incidence of net adverse clinical events (NACE, defined as TLF or BARC type 2–5 bleeding) during the 12 months after enrollment;
10. The incidence of stent thrombosis (ARC definite, probable, or definite/probable) during the 12 months after enrollment.

# 12. STATISTICAL METHODS AND SAMPLE SIZE DETERMINATION

The statistical analyses will be performed using SAS version 9.3.

## 12.1 Description of analysis sets

The Enrolled Population consists of all subjects who signed informed consent.

The full-analysis-set (FAS) population will consist of all subjects who have been randomized (i.e., when the subject number and allocated treatment are recorded in the EDC database) according to the intention-to-treat (ITT) principle. Subjects will be analyzed in the treatment group assigned by the EDC. The primary endpoint of gastric or intestinal mucosal injury will be analyzed in a modified intention to treat (mITT) population, (i.e., patients with valid primary endpoint results collected).

The per-protocol (PP) population will consist of all randomized subjects without any major deviations from the protocol. The following deviations will lead to exclusion from the PP population:

- Subjects not receiving the assigned treatment as allocated by the EDC or no treatment at all.
- Non-compliance to study drug. Non-compliance is defined as taking less than 80% of dispensed tablets based on manual pill bottle count at each study visit.
- Patients with overdue AMCE or gastroscopy examination at visit 3 (180 days ± 2 weeks after randomization).

### 12.1.1 Efficacy analysis set

All efficacy analyses will be performed on the FAS and PP population. The primary efficacy analysis will be performed on the FAS population. This analysis will be repeated in the PP population to support the primary results.

The primary efficacy endpoint gastric or small intestinal mucosal injury occurring within 12 months after enrollment, defined as a composite of erosion, ulceration or bleeding.

### 12.1.2 Safety analysis set

Safety analyses of study medications will be performed on the FAS population. The safety endpoint is MACCE defined as a composite of cardiac death, target lesion MI, ischemic stroke, clinical-driven TLR or stroke within 12 months after enrollment.

Safety analyses of AMCE examination will be performed in patients undergoing AMCE examination (including GI cleansing). The safety endpoints are digestive symptoms (abdominal distension, nausea, or vomiting, etc.) and retention of capsule endoscopy within 30 days.

## 12.2 Methods of statistical analyses

Continuous variables will be summarised as the number of observations, number of missing values, mean, standard deviation, median, quartiles, and range.

Categorical variables will be summarised as the number of observations, number of missing values, frequencies, and percentages.

Baseline clinical, demographic, laboratory and procedural characteristics will be summarized by randomized treatment group.

Demographic and baseline characteristics will be summarized by randomized treatment group and for all randomized subjects combined (i.e., FAS population). Baseline characteristics that will define subgroups of interest for the efficacy and safety analyses are:

- Age group (< 65, ≥ 65 years)
- Gender (Male, Female)
- Diabetes mellitus (yes, no)
- Chronic kidney disease (yes, no)
- CAD presentation (Stable, Unstable)
- HP infection status at enrollment (positive, negative)
- Antiplatelet treatment status at enrollment (>6 months, ≤6 month)

**Censoring:** Subjects not experiencing any endpoint will be censored at time of death, last contact date (for subjects who withdraw consent or are lost to follow-up) or 180-day ± 14 days after randomization, whichever comes first.

**Primary Efficacy Analysis:** The primary endpoint analysis will be conducted according to the intention-to-treat (ITT) principle. The main aim of this study is to determine whether the strategy of 6-month DAPT plus 6-month single antiplatelet therapy is superior to 12-month DAPT in terms of the primary endpoint (gastrointestinal injury) after contemporary DES implantation. The null hypothesis ($H_0$) for this analysis is that the incidence of primary endpoint in the experimental group is same as that of the control group, namely $P_0=P_1$. The alternative hypothesis ($H_1$) is that the incidences of primary endpoints in the two groups was not equal, namely $P_0 \neq P_1$, and the superiority test is conducted at the 2-sided significance level of 0.05. This analysis will be repeated in the Per-protocol (PP) population to support the main results.

The primary and secondary endpoints will be also analyzed in the following clinically

relevant pre-specified subgroups (Part 12.2). Formal interaction testing will be performed using the subgroup × treatment allocation as an additional term in the logistic models.

## 12.3 Determination of sample size

The cumulative incidence of the primary endpoint of gastric or small intestinal mucosal lesions within 12 months is estimated to be 47% in patients who received 12 months of DAPT and 30% in those treated with either aspirin or clopidogrel monotherapy beginning at 6 months after enrollment. With a 2:1 ratio in patients treated with either aspirin or clopidogrel monotherapy (the sum of aspirin monotherapy group and clopidogrel monotherapy group) after 6-month DAPT versus DAPT for 12 months, 384 evaluable patients (256 and 128 respectively) provide 90% power to detect a 17% absolute risk reduction (36% relative risk reduction) with a 2-sided type I error of 0.05. Assuming 20% loss of evaluable primary endpoint outcome assessments due to patient withdrawal, loss to follow-up between 6 and 12 months or suboptimal AMCE visualization of the GI tract at 12months, 480 patients are planned to be randomized. Assuming that an additional 10% of enrolled patients will not be randomized at 6 months because of adverse clinical events, non-compliance with antiplatelet therapy, lost to follow-up or withdrawal, 534 patients is required to be initially planned to be enrolled after baseline screening. Finally, assuming that 10% of patients who undergo a screening AMCE examination will be excluded due to unavailable valid image (or definite ulceration and active bleeding), therefore a total of 593 patients is planned to be consented and undergo the screening AMCE examination.

## 12.4 Clinical event committee

Clinical Events Committee (CEC) will be established, which is composed of a group of independent cardiologists and gastroenterologists not engaged in this study. It is CEC’s responsibility to adjudicate all reported clinical events and categorize these events by definitions. If necessary, CEC will request source data from research sites. CEC members are unaware of which study drugs subjects are assigned. For event definitions see in Appendix 1, include:

- Gastrointestinal mucosal lesions (including erosion, ulceration and gastrointestinal bleeding)
- Gastrointestinal symptoms
- Death
- Recurrent MI
- Ischemia-driven target vessel revascularization

- Stent thrombosis
- Bleeding
- Other.

### 12.5 Data and safety monitoring board

The study will be conducted under the auspices of an independent Data and Safety Monitoring Board (DSMB). DSMB members will not have primary affiliation with the study sponsor, the EDC supplier or the principal investigator of the trial. Members of the Board will be determined prior to study enrollment.

All adverse events will be reported to the DSMB, and if necessary DSMB will ask for more information. DSMB will review data and determine reporting and stopping rules as specified in the DSMB charter. The DSMB members will review undisclosed temporary data, including adjudicated and non-adjudicated TLF, Bleeding, and other Serious Adverse Events and their incidence, in order to identify potential safety issues three months after random. On account of the result of the review, DSMB will provide a report about the safety of the study with the premise of not breaking the blind. Based on the safety data, the DSMB may recommend modifications to the protocol, suspension or termination of the trial, and advise the Executive Committee. Executive Committee should distribute the copy of safety report to all the participating centers. All final decisions, regarding trial modifications, however, rest with the Executive Committee.

## 13. IMPORTANT MEDICAL PROCEDURES TO BE FOLLOWED BY THE INVESTIGATOR

### 13.1 Overdose

If an overdose on the study drug occurs in the course of the study, then investigators or other site personnel inform the study director, the principal investigator and the study monitor in this research site within one day, i.e., immediately but no later than the end of the next business day of when he or she becomes aware of it.

### 13.2 Pregnancy

Due to the study enrollment criteria and proposed population, women of child bearing potential (defined in Part 4.2) are excluded from participation in this study. In the unlikely event that pregnancy should occur during the course of the study, all

outcomes of pregnancy should be reported to the study director, the principal investigator and the study monitor in this research site.

# 14. LIST OF REFERENCES

1. Zhang L, Li Y, Jing QM, et al. Dual antiplatelet therapy over 6 months increases the risk of bleeding after biodegradable polymer-coated sirolimus eluting stents implantation: insights from the CREATE study. J Interv Cardiol 2014;27:119-26.
2. Han Y, Xu B, Xu K, et al. Six Versus 12 Months of Dual Antiplatelet Therapy After Implantation of Biodegradable Polymer Sirolimus-Eluting Stent: Randomized Substudy of the I-LOVE-IT 2 Trial. Circ Cardiovasc Interv 2016;9:e003145.
3. Liao Z, Hou X, Lin-Hu EQ, et al. Accuracy of Magnetically Controlled Capsule Endoscopy, Compared With Conventional Gastroscopy, in Detection of Gastric Diseases. Clin Gastroenterol Hepatol 2016;14:1266-73 e1.
4. Zou WB, Hou XH, Xin L, et al. Magnetic-controlled capsule endoscopy vs. gastroscopy for gastric diseases: a two-center self-controlled comparative trial. Endoscopy 2015;47:525-8.
5. Malfertheiner P, Chan FK, McColl KE. Peptic ulcer disease. Lancet 2009;374:1449-61.
6. Kim BS, Li BT, Engel A, et al. Diagnosis of gastrointestinal bleeding: A practical guide for clinicians. World J Gastrointest Pathophysiol 2014;5:467-78.
7. Thygesen K, Alpert JS, Jaffe AS, et al. Third universal definition of myocardial infarction. J Am Coll Cardiol 2012;60:1581-98.
8. Cutlip DE, Windecker S, Mehran R, et al. Clinical end points in coronary stent trials: a case for standardized definitions. Circulation 2007;115:2344-51.
9. Lanza Fl Fau - Royer GL, Jr., Royer Gl Jr Fau - Nelson RS, Nelson Rs Fau - Chen TT, Chen Tt Fau - Seckman CE, Seckman Ce Fau - Rack MF, Rack MF. A comparative endoscopic evaluation of the damaging effects of nonsteroidal anti-inflammatory agents on the gastric and duodenal mucosa.
10. Scarpignato C, Dolak W, Lanas A, et al. Rifaximin Reduces the Number and Severity of Intestinal Lesions Associated With Use of Nonsteroidal Anti-Inflammatory Drugs in Humans. Gastroenterology 2017;152:980-2 e3.

# 15. APPENDIX 1- DEFINITION FOR STUDY ENDPOINTS

## 15.1 Definition of erosion and ulcer

Gastrointestinal erosion is defined as superficial mucosal breaks with a diameter of b5 mm. Gastrointestinal ulcer is defined as a mucosal break with a diameter ≥5 mm, typically covered with fibrin[5].

## 15.2 Definition and classification of gastrointestinal (GI) bleeding[6]

**Category 1:**

1) Hematemesis: refers to the patient's vomiting of blood due to acute bleeding in the upper gastrointestinal tract (esophagus, stomach, duodenum and jejunum after gastrojejunostomy, pancreas and biliary tract).

2) Hematochezia or melena: blood is discharged from the anus, and the color of the stool is bright red, dark red or tarry black, which is called hematochezia.

3) Positive fecal occult blood (FOB): a small amount of bleeding in the digestive tract, which cannot be confirmed by eyes or under a microscope. The erythrocytes are destroyed by digestion and there is no abnormal change in stool appearance.

**Category 2:**

1) Upper gastrointestinal bleeding: hemorrhage originating from the esophagus to the ligament of Treitz (located at the duodenojejunal flexure).

2) Lower gastrointestinal bleeding: bleeding that originates from a site distal to the ligament of Treitz.

## 15.3 The classification of internal hemorrhoids according to the guidelines of diagnosis and treatment of hemorrhoids of the American Society of Colon and Rectal Surgeons

I: Prominent hemorrhoidal vessels, no prolapse

II: Prolapse with Valsalva and spontaneous reduction

III: Prolapse with Valsalva requires manual reduction

IV: Chronically prolapsed manual reduction ineffective

## 15.4 Bleeding Academic Research Consortium definition of bleeding

### Table 3 Definition of BARC bleeding

| Type | Definition |
|---|---|
| 0 | No evidence of bleeding. |
| 1 | Bleeding that is not actionable and patient does not have unscheduled studies, hospitalization or treatment by a health care professional. |
| 2 | Any clinically overt sign of hemorrhage that is actionable but does not meet criteria for type 3, 4 or 5 bleeding. It must meet at least one of the following criteria:<br>1) requiring medical or percutaneous intervention guided by a health care profession, includes (but are not limited to) temporary/permanent cessation of a medication, coiling, compression, local injection;<br>2) leading to hospitalization or an increased level of care;<br>3) prompting evaluation defined as an unscheduled visit to a healthcare professional resulting in diagnostic testing (laboratory or imaging). |
| 3 | Clinical, laboratory and/or imaging evidence of bleeding with specific healthcare provider responses, as listed below: |
| 3a | 1) Any transfusion with overt bleeding;<br>2) Overt bleeding plus hemoglobin (Hb) drop ≥3 to <5g/dL * (provided Hb drop is related to bleeding). |
| 3b | 1) Overt bleeding plus Hb drop ≥5g/dL* (Hb drop is related to bleed);<br>2) Cardiac tamponade;<br>3) Bleeding requiring surgical intervention for control (excluding dental/nasal/skin/hemorrhoid);<br>4) Bleeding requiring intravenous vasoactive drugs. |
| 3c | 1) Intracranial hemorrhage (does not include microbleeds or hemorrhagic transformation; does include intraspinal). Subcategories: confirmed by autopsy, imaging or lumbar puncture;<br>2) Intraocular bleed compromising vision. |
| 4 | CABG – Related Bleeding<br>1) Perioperative intracranial bleeding within 48 hours;<br>2) Reoperation following closure of sternotomy for the purpose of controlling bleeding;<br>3) Transfusion of ≥5 units of whole blood or packed red blood cells within a 48-hour period;<br>4) Chest tube output ≥2L within a 24 hour period. |
| 5 | Fatal Bleeding. Bleeding directly causes death with no other explainable cause. Categorized further as either definite or probable. |
| 5a | Probable fatal bleeding is bleeding that is clinically suspicious as the cause of death, but the bleeding is not directly observed and there is no autopsy or confirmatory imaging. |
| 5b | Definite fatal bleeding is bleeding that is directly observed (either by clinical specimen – blood, emesis, stool, etc. – or by imaging) or confirmed on autopsy. |

## 15.5 Major Adverse Cardiovascular and Cerebrovascular Events

Major cardiovascular and cerebrovascular adverse events (MACCE) is death, recurrent MI, stroke and target vessel revascularization. The definitions of the individual components of MACE are given below.

### 15.5.1 Death

All-cause death comprises several subclassifications (Table 4). In general, all deaths are considered cardiac unless an alternate cause is unequivocally established, even among subjects with serious noncardiac comorbidities.

**Table 4 Classification of death**

| | |
|---|---|
| Cardiac death | Any death due to proximate cardiac cause (e.g., MI, low-output failure, fatal arrhythmia), unwitnessed death and death of unknown cause, and all procedure-related deaths, including those related to concomitant treatment, will be classified as cardiac death. |
| Vascular death | Death caused by noncoronary vascular causes, such as cerebrovascular disease, pulmonary embolism, ruptured aortic aneurysm, dissecting aneurysm, or other vascular diseases. |
| Nonvascular death | Any death not covered by the above definitions, such as death caused by infection, malignancy, sepsis, pulmonary causes, accident, suicide, or trauma. |

### 15.5.2 Myocardial infarction

According to the third universal definition of myocardial infarction[7], myocardial infarction is classified as follows:

- Type 1: Spontaneous myocardial infarction
- Type 2: Myocardial infarction secondary to an ischemic imbalance
- Type 3: Cardiac death infarction without available biomarker values due to myocardial infarction
- Type 4a: MI associated with PCI
- Type 4b: MI associated with stent thrombosis confirmed by angiography or at autopsy
- Type 5: MI associated with CABG.

Any of the following criterion meets the diagnosis of MI:

- Increased and/or decreased levels of cardiac biomarkers (preferred cardiac troponin) with at least one value above the 99th percentile upper reference limit (URL) plus at least one of the following:
    - Symptoms of ischemia;
    - New ST-T changes or new left bundle branch block (LBBB)
    - pathological Q waves
    - Imaging evidence of new loss of viable myocardium or new regional wall motion abnormality in a pattern consistent with an ischemic etiology;
    - Identification of a coronary thrombus by angiography or autopsy.
- Cardiac death with symptoms suggestive of myocardial ischemia and presumed new ischemic ECG changes or new LBBB, but death occurring before blood samples could be obtained, before cardiac biomarker could rise
- PCI-related MI is determined by the elevation of myocardial biomarkers:
    - The elevation of cTn values >5 x 99th percentile URL in patients with normal baseline values (99th percentile URL) or
    - The rise of cTn values >20% if the baseline values are elevated and are stable or falling.

In addition, meeting at least one of the following

- Symptoms suggestive of myocardial ischemia,
- New ischemic ECG changes or new LBBB,
- Angiographic loss of patency of a major coronary artery or a side branch or persistent slow or no-flow or embolization.
- Imaging demonstration of new loss of viable myocardium or new regional wall motion abnormality are required

### 15.5.3 Clinically-driven target vessel revascularization

Clinically-driven revascularization of target vessels includes the revascularization of the previously implanted vessels due to recurring or persistent ischemic symptoms. The revascularization is defined in accordance with the relationship with the target vessels[8].

## 15.6 Stent thrombosis[8]

Stent thrombosis is classified according to the level of certainty and timing following PCI (Table 5).

- Definite stent thrombosis: Angiographic or pathological confirmation of the presence of a thrombus that originates in the stent or in the segment 5 mm proximal or distal to the stent and presence of at least 1 of the following criteria within a 48-hour time window:
    - Acute onset of ischemic symptoms at rest
    - New ischemic ECG changes that suggest acute ischemia
    - Typical rise and fall in cardiac biomarkers (refer to definition of spontaneous MI)
- Probable stent thrombosis
    - Any unexplained death within the first 30 days
    - Irrespective of the time after the index procedure, any MI that is related to documented acute ischemia in the territory of the implanted stent without angiographic confirmation of stent thrombosis and in the absence of any other obvious cause
- Possible stent thrombosis
    - Clinical definition of possible stent thrombosis is considered to have occurred with any unexplained death from 30 days after intracoronary stenting until end of trial follow-up

**Table 5 The classification according to the timing of stent thrombosis**

| **Classification** | **Time of occurrence** |
|---|---|
| Acute stent thrombosis | 0 to 24 hours after stent implantation |
| Subacute stent thrombosis | >24 hours to 30 days after stent implantation |
| Late stent thrombosis | >30 days to 1 year after stent implantation |
| Very late stent thrombosis | >1 year after stent implantation |

# 16. APPENDIX 2 - MAGNETICALLY CONTROLLED CAPSULE ENDOSCOPY SCORING SYSTEM

## 16.1 The gastric mucosal injury is evaluated through the Lanza score[9]

**Table 6 Lanza score**

| Endoscopic manifestation | score |
|---|---|
| No erosion | **0** |
| 1–2 erosions localized in the gastric antrum, body or bottom | **1** |
| 3–5 erosions localized in one area of the stomach | **2** |
| Erosions localized in 2 different areas of the stomach (total 6–9 lesions) | **3** |
| Gastric ulcer or ≥ 10 erosions | **4** |

Efficacy evaluation standard:
the proportion of mild gastric mucosal injury = cases with 1 point and 2 points / total cases × 100%
The proportion of severe gastric mucosal injury = cases with 3 points + 4 points / total cases × 100%

## 16.2 Five-point scoring system for small intestinal mucosal injury[10]

**Table 7 Five-point scoring system to assess intestinal mucosal injury**

| Category | Score |
|---|---|
| Normal | **0** |
| Petechiae/red spot (demarcated, usually circular, area of crimson mucosa with preservation of villi) | **1** |
| Small number of erosions (1–4 erosions) | **2** |
| High number of erosion (>4 erosions) | **3** |
| Mucosal breaks (large erosion and/or ulcer) | **4** |

# 17. APPENDIX 3 – GASTROINTESTINAL SYMPTOM SCORE

Symptoms are categorized as four types: abdominal pain, bloating, acid reflux, and belching.

Each symptom includes a five-level scoring system (degree and frequency are scored separately; the maximum score = 4 + 4 = 8 points).

**Table 8 Five-point scoring system to assess gastrointestinal symptoms**

| Score | Symptom | Frequency |
|---|---|---|
| **0** | No symptom | |
| **1** | Mild: mild symptoms, need to pay attention to feel | once per week |
| **2** | Moderate: self-conscious obvious symptoms, but does not affect work and life | 2-3 times per week |
| **3** | Severe: self-conscious obvious symptoms, affect work and life | 4-5 times per week |
| **4** | Extremely severe: self-conscious very obvious symptoms, seriously affecting work and life | Almost daily or consistent |

# 18. APPENDIX 4 – TREATMENT PRINCIPLES OF GASTROINTESTINAL BLEEDING AND MUCOSAL INJURY

Regulation rules in Cardiology
DAPT should be discontinued immediately. After three days after stopping the bleeding by electronic gastroscopy:

1) If gastrointestinal injury is entirely controlled without reoccurrence of active bleeding, and hemoglobin is maintained at the level of ≥9 g/dL (90 g/L), clopidogrel monotherapy (75 mg, daily) should be firstly administered as soon as possible and aspirin (100 mg, daily) should be resumed at the 5$^{th}$ day after hemostasis. Times, types and doses of medications should be recorded in detail.
2) If active minor or occult bleeding still exists, or the level of hemoglobin fluctuates above 10 g/L in different days (monitor the blood routine

test if necessary), only low molecular weight heparin (LMWH, hypo, daily) is used. Then at the 7th day after hemostasis, stop the LMWH and resume DAPT (doses and frequencies as above).

## 18.1 Fecal Occult Blood (FOB) Tests

Precautions before testing:

1) Do not test during the period of menstrual bleeding, hemorrhoid bleeding or anal fissure;

2) To avoid affecting the judgment of FOB results, it is forbidden to take iron supplement, meat, liver, blood, and green vegetables within three days before the examination to avoid affecting the judgment of FOB results.

**Treatment of patients with positive results:**

**Before randomization:** For patients with positive FOB result, the test should be repeated for 2 consecutive days, and the complete blood count should be performed at the same time. If the FOB test is continuously positive with a decrease in hemoglobin (a drop of more than 10g/L compared to baseline screening), AMCE should be performed to confirm the lesion (for free), and even if no clear lesions are found, it should be considered that occult small intestinal bleeding exists. The patient will be treated according to the principle of gastrointestinal bleeding treatment, and is ineligible for study enrollment. If the continuously positive FOB result continues without hemoglobin reduction, the blood routine test should be monitored at the same time as each fecal occult blood test. If the hemoglobin drops more than 10g/L (compared with the first blood routine test after positive FOB result), AMCE should be performed to confirm the lesion (for free). Then the patient will be treated according to the principle of gastrointestinal bleeding treatment and is ineligible for study enrollment. If FOB test turns negative in a repeated test, no treatment will be given.

**After randomization:** For patients with positive FOB result, the test should be repeated for 2 consecutive days, and the complete blood count should be performed at the same time. If the FOB test is continuously positive with a decrease in hemoglobin (a drop of more than 10g/L compared to baseline screening), AMCE should be performed to confirm the lesion (for free), and even if no clear lesions are found, it should be considered that occult small intestinal bleeding exists. Breaking the blinding shall be performed immediately, and the patient will be treated according to the principle of gastrointestinal bleeding treatment. If the continuously positive FOB result continues without hemoglobin reduction, the blood routine test should be monitored at the same time as each fecal occult blood test. If the hemoglobin drops more than 10g/L (compared with the first blood routine test after positive FOB result),

AMCE should be performed to confirm the lesion (for free), and even if no clear lesions are found, it should be considered that occult small intestinal bleeding exists. Breaking the blinding shall be performed immediately, and the patient will be treated according to the principle of gastrointestinal bleeding treatment. The identification of time of the gastrointestinal bleeding event is the date of the first positive FOB result. If FOB test turns negative in a repeated test, no treatment will be given.

## 18.2 The treatment procedure of clinically-driven or AMCE-positive gastrointestinal (GI) bleeding

1) The patient with GI bleeding before randomization will be treated in accordance with the following (Appendix 1-3) rules. The patient will be no longer randomized and the study is terminated.

2) The patient with GI bleeding after randomization should be confirmed by gastroscopy, and endoscopic treatment is taken if necessary. If the lesion is not clear, the AMCE examination is performed to observe the small intestine lesions. If no clear bleeding is found, colonic bleeding is suspected, and further colonoscopy should be performed.

3) After obtaining the consent of principal of the participating research site, the treating physician could unblind for this patient (Part 5.4), conduct hemostatic treatment and adjust antiplatelet therapy according to the following rules (Appendix 1-3). Antiplatelet therapy could be at physician's discretion according to local standard care after curing the bleeding.

4) This patient will bot terminate study, but only clinical 12-month follow-up (without AMCE examination) is needed.

## 18.3 The treatment principle of clinically-driven or AMCE-positive gastrointestinal (GI) bleeding

**Gastroenterology treatment rules:**

1) Patients with a definite diagnosis of GI bleeding should undergo endoscopic hemostasis as soon as possible with stable hemodynamics. The specific hemostasis methods can be implemented according to the experience of the endoscopy center of each hospital, such as hemostatic clips, APC, rinse with ice adrenal solution, heat probe, etc., plus PPI treatment. Medications: for gastric ulcer, PPI + gastric mucosal protective agent for 6 weeks, for duodenal ulcer, PPIs for 4 weeks. If patients with Helicobacter pylori (HP) infection requires eradication therapy, Quadruple therapy for 2 weeks could be performed.

2) Patients with GI bleeding and shock should be treated in accordance with routine treatment such as hemorrhagic shock monitoring, blood transfusion, fluid infusion, and hemostasis.

**Table 2 The specific procedures**

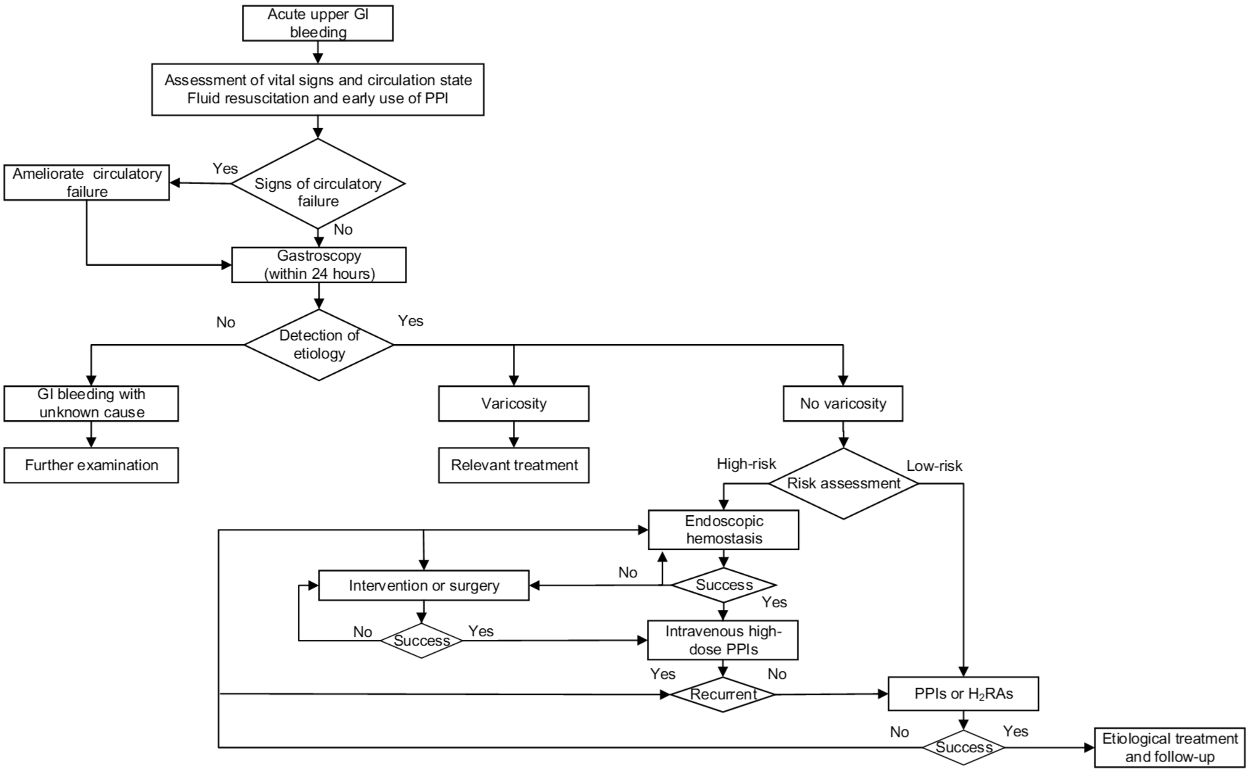


**Cardiology treatment rules:**

DAPT should be discontinued immediately. After three days after stopping the bleeding by electronic gastroscopy:

1) If gastrointestinal injury is entirely controlled without reoccurrence of active bleeding, and hemoglobin is maintained at the level of ≥9 g/dL (90 g/L), clopidogrel monotherapy (75 mg, daily) should be firstly administered as soon as possible and aspirin (100 mg, daily) should be resumed at the 5th day after hemostasis. Times, types and doses of medications should be recorded in detail.

2) If active minor or occult bleeding still exists, or the level of hemoglobin fluctuates above 10 g/L in different days (monitor the blood routine test if necessary), only low molecular weight heparin (LMWH, hypo, daily) is used. Then at the 7th day after hemostasis, stop the LMWH and resume DAPT (doses and frequencies as above).

## 18.4 Judgment criteria and treatment rules of severe gastrointestinal mucosal lesions requiring PPI

**Principles of PPI:** PPI and/or gastric mucosal protective agent should only be added to diagnosed ulcer lesions. No special treatment will be given if only an erosion exists.

**Treatment of peptic ulcer:**

Patients with diagnosed peptic ulcers should be treated with PPI. Gastric ulcer: PPI + gastric mucosal protective agent for 6 weeks, duodenal ulcer: PPI for 4 weeks.

For HP-positive patients, if requested by the patient, a quadruple therapy eradication treatment for 2 weeks could be given (PPI + bismuth + two kinds of antibiotics, PPI: omeprazole 20 mg bid; lansoprazole 30 mg, bid; Pantoprazole 40mg, bid; Rabeprazole 20mg, bid; Esomeprazole 20mg, bid; Bismuth: colloidal bismuth subcitrate 110mg 4 times per day; antibiotic: amoxicillin 1000mg, bid + clarithromycin 500mg, bid; amoxicillin 1000mg, bid + levofloxacin 500mg, qd; amoxicillin 1000mg, bid + furazolidone 100mg, bid; tetracycline 750mg, bid + furazolidone 100mg, bid; tetracycline 750mg, bid + metronidazole 400mg, bid or tid).

# OPT-PEACE Study

Optimal antiplatelet therapy for prevention of gastrointestinal injury evaluated by ANKON magnetically controlled capsule endoscopy

**Sponsor:** General Hospital of Northern Theatre Command

**Version** 1.4

**Contract Research Organization:** ExcellentCRO Co, Ltd, Shenyang, China.

# PROTOCOL SYNOPSIS

| | |
|---|---|
| **Title of Study** | OPT-PEACE Study - Optimal antiplatelet therapy for prevention of gastrointestinal injury evaluated by ANKON magnetically controlled capsule endoscopy |
| **Principal Investigator:** | Yaling Han, MD, PhD, FACC<br>Professor of Medicine, Director of Department of Cardiology, General Hospital of Northern Theatre Command and Academician of Chinese Academy engineering |
| **Co-principal Investigators:** | Zhaoshen Li, MD, PhD<br>Professor of Medicine, Director of Department of Gastroenterology, Changhai Hospital, Second Military Medical University/Naval Medical University and Academician of Chinese Academy engineering<br><br>G.W Stone, MD, PhD, FACC<br>Professor of Medicine, Director of Columbia University Medical Center<br><br>Xiaozeng Wang, MD<br>Professor of Medicine, Deputy Director of Department of Cardiology, General Hospital of Northern Theatre Command |
| **Study Centers:** | 27 sites in China |
| **Study Design** | Multicenter, prospective, randomized, double-blinded, placebo-controlled study |
| **Purposes** | The primary study objective is to determine the risks of 12 months of DAPT vs 6 months of DAPT followed by 6 months of aspirin monotherapy or clopidogrel monotherapy on gastrointestinal mucosal injury after DES implantation.<br>The secondary objective is to evaluate the feasibility and safety of AMCE as a method for detecting gastrointestinal mucosal injury and bleeding in patients receiving APT.<br>The exploratory objective is to establish a gastrointestinal mucosal injury scoring system that may identify patients at future risk for clinical gastrointestinal bleeding during long-term APT. |
| **Randomization** | All enrolled patients after PCI firstly received DAPT as a composite of aspirin plus clopidogrel for 6 months, and are then randomly assigned in a 1:1:1 ratio to receive:<br>A: aspirin plus clopidogrel, for an additional 6 months;<br>B: aspirin plus clopidogrel-placebo, for an additional 6 months;<br>C: aspirin-placebo plus clopidogrel plus for an additional 6 months. |

| | |
|---|---|
| **Primary Endpoint** | The incidence of gastric or intestinal mucosal injury occurring within 12 months after enrollment, defined as erosion, ulceration or bleeding detected by either planned AMCE or clinically-driven endoscopy. |
| **Secondary Endpoints** | 1. The incidence and severity of gastric and intestinal mucosal lesions during the first 6 months after study enrollment (prior to randomization);<br>2. The incidence and severity of gastric and intestinal mucosal lesions after randomization (i.e., between 6 months and 12 months after study enrollment);<br>3. The incidence of clinically evident gastrointestinal hemorrhage attributed to the upper GI tract (or of unknown origin) during 6 months after study enrollment (prior to randomization);<br>4. The incidence of clinically evident gastrointestinal hemorrhage attributed to the upper GI tract (or of unknown origin) after randomization (i.e., between 6 months and 12 months after study enrollment);<br>5. The incidence of clinically evident gastrointestinal hemorrhage attributed to the upper GI tract (or of unknown origin) during 12 months after study enrollment;<br>6. Gastrointestinal symptoms (pain, nausea/vomiting, dysphagia, other) during the 12 months after enrollment;<br>7. All bleeding (BARC types 1–5) during the 12 months after enrollment;<br>8. The incidence of target lesion failure (TLF; cardiac death, target-vessel MI, or clinically-driven target lesion revascularization), during the 12 months after enrollment;<br>9. The incidence of net adverse clinical events (NACE, defined as TLF or BARC type 2–5 bleeding) during the 12 months after enrollment;<br>10. The incidence of stent thrombosis (ARC definite, probable, or definite/probable) during the 12 months after enrollment. |
| **Inclusion Criteria** | (All must be present)<br>1. Adult patients with age 18–80 years;<br>2. Presentation with stable angina, or non-ST-segment elevation acute coronary syndrome with GRACE score <140 at admission;<br>3. PCI with implantation of contemporary drug-eluting stent(s) during the present admission;<br>4. Complete revascularization (successful PCI treatment of all epicardial coronary lesions with diameter stenosis ≥70% or intermediate lesions with FFR <0.80);<br>5. Planned DAPT with aspirin and clopidogrel for at least 6 months;<br>6. Agreement to comply with all study procedures;<br>7. Written informed consent provided. |

| | |
|---|---|
| **Exclusion Criteria** | (All must be absent)<br>1. Presentation with STEMI, or NSTE-ACS with GRACE score ≥140;<br>2. Left main disease (diameter stenosis >30%);<br>3. Any prior coronary stent implantation during the last year prior to the index procedure;<br>4. Implantation of first-generation drug-eluting stents or bioabsorbable scaffolds during the index procedure;<br>5. Implantation of >4 stents during the index procedure;<br>6. Any prior stent thrombosis;<br>7. Any active gastrointestinal bleeding or ulcers, or prior gastrointestinal bleeding or ulcers within the last 24 months;<br>8. Prior gastrointestinal tract or abdominal surgery other than simple procedures which would not change the gastrointestinal tract anatomy, such as polyp removal, cholecystectomy or appendectomy;<br>9. Contraindications to the AMCE test, including suspected or known gastrointestinal obstruction, stenosis, fistula, diverticula, etc.; presence of gastrointestinal obstruction symptoms such as pain or dysphagia; inoperative conditions or refusal to undergo abdominal surgery if required (because once the capsule could not pass the tract, a surgery may be needed);<br>10. Severe hemorrhoids (phase 3–4 according to guidelines of American Society of Colon and Rectal Surgery);<br>11. LVEF <0.40 on admission by echocardiography;<br>12. Renal dysfunction (eGFR <30 ml/min/1.73m$^2$);<br>13. Active hepatitis or ALT >3 times upper limits of normal at admission;<br>14. Severe hypertension (>180/110 mmHg), or hypertension without control;<br>15. Hemoglobin <100 g/L;<br>16. Platelet count <100 × 10$^9$/L;<br>17. Unable to restrict use of PPI, gastric mucosa protectant or any other antacid agent by rule (details in "restrictions during the study")<br>18. Required use of oral anticoagulation (warfarin or other factor II or factor X inhibitors);<br>19. Inability to take 12-month DAPT for any reason;<br>20. Mandatory use of >6-month DAPT (indicating those who are not suitable to receive aspirin or clopidogrel monotherapy);<br>21. Any comorbidity with estimated survival time <12 months (e.g., progressive cancer, chronic obstructive lung disease, etc.);<br>22. Any contraindication to MRI examination, including implantation of an MRI-incompatible pacemaker, defibrillator, or other ferromagnetic material, etc.;<br>23. Pregnant or plan to be pregnant;<br>24. Any condition that may interfere with any study procedures, such as dementia, immobility, alcohol use, etc.;<br>25. Planned surgery within 1 year;<br>26. Taking iron supplement;<br>27. Participating in any other clinical trial of an investigational drug or device that has not met its primary endpoint. |

| | |
|---|---|
| **Evaluation of Randomization Eligibility** | Eligibility for randomization will be evaluated in all enrolled patients at 6 months after PCI. At this time patients with any of the following exclusion criteria will be suspended for randomization.<br>1. Withdrawal of informed consent;<br>2. Lost to follow-up at 6 months;<br>3. Any event in the prior 6 months which in the opinion of the investigator results in the patient not being suitable for randomization of antiplatelet agent regimen either because of a) necessity to continue dual antiplatelet therapy (e.g., major adverse cardiovascular or cerebrovascular event within the prior 6 months or need for repeat stenting), or b) inability to continue dual antiplatelet therapy (e.g., bleeding, neoplasm, need for urgent surgery, etc. within the prior 6 months);<br>4. Not presently taking both aspirin and clopidogrel, or any prior temporary discontinuation of aspirin or clopidogrel for ≥5 days;<br>5. Use of proton pump inhibitors or gastric mucosal protectants for more than 12 days, or for more than 4 continuous days in the 6 months prior to randomization;<br>6. Unwillingness or inability to undergo the 6-month AMCE examination or the remainder of the study procedures, including the 12-month AMCE exam. |
| **Statistical Methods** | |
| **Primary Endpoint Analysis** | The primary endpoint analysis will be conducted according to the intention-to-treat (ITT) principle. The main aim of this study is to determine whether the strategy of 6-month DAPT plus 6-month single antiplatelet therapy is superior to 12-month DAPT in terms of the primary endpoint (gastrointestinal injury) after contemporary DES implantation. The null hypothesis ($H_0$) for this analysis is that the incidence of primary endpoint in the experimental group is same as that of the control group, namely $P_0=P_1$. The alternative hypothesis ($H_1$) is that the incidences of primary endpoints in the two groups was not equal, namely $P_0 \neq P_1$, and the superiority test is conducted at the 2-sided significance level of 0.05. This analysis will be repeated in the Per-protocol (PP) population to support the main results. |

| | |
|---|---|
| **Sample Size Determination and Adjustment** | The cumulative incidence of the primary endpoint of gastric or small intestinal mucosal lesions within 12 months is estimated to be 47% in patients who received 12 months of DAPT and 30% in those treated with either aspirin or clopidogrel monotherapy beginning at 6 months after enrollment. With a 2:1 ratio in patients treated with either aspirin or clopidogrel monotherapy (the sum of aspirin monotherapy group and clopidogrel monotherapy group) after 6-month DAPT versus DAPT for 12 months, 384 evaluable patients (256 and 128 respectively) provide 90% power to detect a 17% absolute risk reduction (36% relative risk reduction) with a 2-sided type I error of 0.05. Assuming 20% loss of evaluable primary endpoint outcome assessments due to patient withdrawal, loss to follow-up between 6 and 12 months or suboptimal AMCE visualization of the GI tract at 12months, 480 patients are planned to be randomized. Assuming that an additional 10% of enrolled patients will not be randomized at 6 months because of adverse clinical events, non-compliance with antiplatelet therapy, lost to follow-up or withdrawal, 534 patients is required to be initially planned to be enrolled after baseline screening. Finally, assuming that 10% of patients who undergo a screening AMCE examination will be excluded due to unavailable valid image (or definite ulceration and active bleeding), therefore a total of 593 patients is initially planned to be consented and undergo the screening AMCE examination.<br>Among the first 200 patients enrolled, ~25% had gastrointestinal injury at baseline by screening AMCE (despite clinically absent bleeding or gastrointestinal complaints). Of those who passed the initial exam, only ~65% were eligible for randomization; 17% of patients were noncompliant with the 6-month repeat AMCE exam, and new gastrointestinal ulceration or bleeding was found on the 6-month AMCE examination in 18% of patients. The study sample size was adjusted accordingly so 1000 patients will be screened by AMCE at baseline, with 750 patients enrolled and followed to the 6-month randomization eligibility period to achieve the 480 patients randomized goal. |

**TABLE OF CONTENTS PAGE**

**TITLE PAGE** ........ **1**
**PROTOCOL SYNOPSIS** ........ **2**
**PROTOCOL SIGNATURE PAGE** ........ **10**
**1. INTRODUCTION** ........ **11**
**1.1 Study Hypothesis** ........ **12**
**1.2 Rationale of Study** ........ **12**
**1.3 Benefit/risk and ethical assessment** ........ **13**
**2. STUDY OBJECTIVES** ........ **13**
**2.1 Primary Objective** ........ **13**
**2.2 Secondary Objective** ........ **13**
**2.3 Exploratory Objective** ........ **13**
**3. STUDY DESIGN AND FLOW CHART** ........ **14**
**4. SUBJECT SELECTION CRITERIA** ........ **14**
**4.1 Inclusion criteria** ........ **14**
**4.2 Exclusion criteria** ........ **15**
**5. STUDY CONDUCT** ........ **16**
**5.1 Restrictions during the study** ........ **16**
**5.2 Screening before enrollment** ........ **17**
**5.3 Subject randomization and initiation of investigational product** ........ **17**
**5.4 Procedures for handling subjects incorrectly enrolled or randomized on investigational product** ........ **18**
**5.5 Blinding and procedures for unblinding the study** ........ **18**
5.5.1 Methods for ensuring blinding ........ 19
5.5.2 Methods for breaking the blinding in the study ........ 19
5.5.3 Methods for unblinding in the study ........ 19
**5.6 Treatments** ........ **19**
5.6.1 Identity of investigational product ........ 20
5.6.2 Doses and treatment regimens ........ 20
5.6.3 Rationale and procedures of AMCE ........ 21
5.6.4 Labeling ........ 22
**5.7 Concomitant and post-study treatment** ........ **22**
**5.8 Treatment compliance** ........ **22**
**5.9 Discontinuation of investigational product** ........ **22**
5.9.1 Temporary discontinuation from Study Medication ........ 22
5.9.2 Permanent discontinuation from Study Medication due to reasons below: ........ 23
**5.10 Withdrawal from study** ........ **23**
**6. COLLECTION OF STUDY VARIABLES** ........ **24**
**6.1 Recording of data** ........ **24**
**6.2 Data collection at enrollment and follow-up** ........ **24**
6.2.1 Enrollment procedures ........ 26
6.2.2 Face-to face follow-up procedure at 6 months ........ 26
6.2.3 Face-to face follow-up procedure at 12 months (for randomized subjects)

.......................................................................................................27
**6.3 Efficacy and safety variables ........................................................... 27**
**7. SAFETY ........................................................................................ 27**
**7.1 Definition of adverse events ............................................................ 27**
**7.2 Definitions of serious adverse events.................................................. 28**
**7.3 Recording of adverse events or serious adverse event................................ 28**
7.3.1 Time period for collection of adverse events.....................................28
7.3.2 Follow-up of unresolved adverse events...........................................28
7.3.3 Information to be collected for each AE/SAE ....................................29
7.3.4 Adverse Events based on signs and symptoms...................................29
7.3.5 Adverse Events based on examinations and tests ...............................30
7.3.6 Disease progression or pre-existing conditions ..................................30
7.3.7 Reporting of adverse events.........................................................30
**8. ETHICAL AND REGULATORY REQUIREMENTS........................................ 30**
**8.1 Ethical conduct of the study............................................................ 30**
**8.2 Ethics and regulatory review ........................................................... 31**
**8.3 Informed consent ........................................................................ 31**
**8.4 Changes to the protocol and informed consent form.................................. 32**
**8.5 Deviations from protocol................................................................ 32**
8.5.1 Compliance to protocol...............................................................32
8.5.2 Procedures for recording, reporting, and analysing protocol deviations
.......................................................................................................33
**8.6 Audits and inspections.................................................................. 33**
**9. STUDY MANAGEMENT ................................................................... 33**
**9.1 Training.................................................................................... 33**
9.1.1 Training of Monitors..................................................................33
9.1.2 Training of study site research personnel.........................................33
**9.2 Monitoring of the study................................................................. 34**
**9.3 Study timetable and end of study ...................................................... 35**
**10. DATA MANAGEMENT ................................................................... 35**
**11. EVALUATION AND CALCULATION OF VARIABLES................................ 35**
**11.1 Primary Endpoint ....................................................................... 35**
**11.2 Secondary Endpoint..................................................................... 36**
**12. STATISTICAL METHODS AND SAMPLE SIZE DETERMINATION.................. 36**
**12.1 Description of analysis sets............................................................ 37**
12.1.1 Efficacy analysis set.................................................................37
12.1.2 Safety analysis set....................................................................37
**12.2 Methods of statistical analyses......................................................... 38**
**12.3 Determination of sample size.......................................................... 37**
**12.4 Clinical event committee .............................................................. 39**
**12.5 Data and safety monitoring board .................................................... 40**
**13. IMPORTANT MEDICAL PROCEDURES TO BE FOLLOWED BY THE INVESTIGATOR ...................................................................................... 40**
**13.1 Overdose ................................................................................. 40**

**13.2 Pregnancy** ........ 41
**14. LIST OF REFERENCES** ........ 42
**15. APPENDIX 1- DEFINITION FOR STUDY ENDPOINTS** ........ 43
**15.1 Definition of erosion and ulcer** ........ 43
**15.2 Definition and classification of gastrointestinal (GI) bleeding[6]** ........ 43
**15.3 The classification of internal hemorrhoids according to the guidelines of diagnosis and treatment of hemorrhoids of the American Society of Colon and Rectal Surgeons** ........ 43
**15.4 Bleeding Academic Research Consortium definition of bleeding** ........ 44
**15.5 Major Adverse Cardiovascular and Cerebrovascular Events** ........ 45
15.5.1 Death ........ 45
15.5.2 Myocardial infarction ........ 45
15.5.3 Clinically-driven target vessel revascularization ........ 46
**15.6 Stent thrombosis[8]** ........ 47
**16. APPENDIX 2 - MAGNETICALLY CONTROLLED CAPSULE ENDOSCOPY SCORING SYSTEM** ........ 48
**16.1 The gastric mucosal injury is evaluated through the Lanza score[9]** ........ 48
**16.2 Five-point scoring system for small intestinal mucosal injury[10]** ........ 48
**17. APPENDIX 3 – GASTROINTESTINAL SYMPTOM SCORE** ........ 49
**18. APPENDIX 4 – TREATMENT PRINCIPLES OF GASTROINTESTINAL BLEEDING AND MUCOSAL INJURY** ........ 49
**18.1 Fecal Occult Blood (FOB) Tests** ........ 50
**18.2 The treatment procedure of clinically-driven or AMCE-positive gastrointestinal (GI) bleeding** ........ 51
**18.3 The treatment principle of clinically-driven or AMCE-positive gastrointestinal (GI) bleeding** ........ 51
**18.4 Judgment criteria and treatment rules of severe gastrointestinal mucosal lesions requiring PPI** ........ 53

# PROTOCOL SIGNATURE PAGE

I have read this clinical investigation plan and appendices and agree to adhere to the requirements. I will provide copies of this clinical investigation plan and all pertinent information to the trial personnel under my supervision. I will discuss this material with them and ensure they are fully informed regarding the device and the conduct of the trial.

I will conduct the trial in accordance with the clinical investigation plan, Good Clinical Practice guidelines, the Declaration of Helsinki, EN ISO 14155:2011 (Clinical Investigation of Medical Devices for Human Subjects - Good Clinical Practice), as well as local regulations. I also accept respective revisions to the clinical investigation plan approved by authorized personnel of the ARO and by regulatory authorities.

*Do not copy, distribute, or share this document with others without prior written authorization.*

Investigator name (print):

Investigator name (signature):

Date:

Institution Name (print):

# 1. INTRODUCTION

Antiplatelet therapy (APT) including aspirin and P2Y12 inhibitors has been the cornerstone for prevention of coronary heart disease (CAD), and current guidelines recommend long-term dual antiplatelet therapy (DAPT) with aspirin in combination with one kind of P2Y12 inhibitor after percutaneous coronary intervention (PCI). However, APT may have serious adverse consequences, the most common of which is gastrointestinal mucosal injury with ulceration and bleeding. The frequency of gastrointestinal complications increases with increasing duration of DAPT. Trials in patients treated with contemporary drug-eluting stents (DES) have demonstrated that shortened DAPT regimens reduce the risk of major bleeding[1] with similar ischemic risk[2]. However, whether there is no definite evidence by current studies exploring the difference of gastrointestinal mucosal injuries among different antiplatelet strategies, mainly due to the absence of sensitive, noninvasive and acceptant methods of detecting gastrointestinal injury.

Nowadays, gastroscopy is the most common detection method for gastrointestinal mucosal injury. Although with a good diagnostic accuracy, gastroscopy is an invasive inspection, and have several limitations below in aspect of screening gastrointestinal bleeding: 1) gastroscopy is invasive and is intolerable for patients, therefore only be used to identify the position and reason of bleeding in patients who have experienced major gastrointestinal bleeding, and is lack of early prediction and early warning effect on gastrointestinal injury and bleeding; 2) Because of the fear of major bleeding caused by the mechanical injury of conducting gastroscopy, a large proportion of gastroenterologists usually asked patients to stop antiplatelet therapy for several days before gastroscopy; 3) upper endoscopy can only detect lesions in the stomach and duodenum, as it does not visualize the remainder of the small intestine.

ANKON® magnetically controlled capsule endoscopy (AMCE) is a novel, noninvasive, actively and precisely controlled system which could visualize gastrointestinal injuries in the gamut of esophagus, stomach and small intestine (except colon). The advantages of AMCE includes: 1) Patient acceptance of AMCE is higher than standard endoscopy as the procedure involves only swallowing a small capsule endoscope with non-invasive, painless, convenient and repeatable image observation; 2) Discontinuation of antiplatelet drugs during AMCE is not necessary, as a result of avoiding the risk of stent thrombosis induced by antiplatelet discontinuation; 3) Not only the stomach and duodenum, but the whole small intestine digestive tract can be detected.

Because of the above advantages, this method can be used for the early evaluation of gastrointestinal mucosal lesions associated with bleeding, and can help detect early focal and concealed bleeding, thus playing as an early warning role a guidance for clinical practice. Previous studies have confirmed that the sensitivity and specificity of AMCE for the detection of focal lesions of the gastrointestinal tract are similar compared with standard endoscopy[3,4]. In this study, we plan to use AMCE as the

evaluation method for gastrointestinal mucosal injury and bleeding, and establish a scoring system for gastrointestinal mucosal injury caused by antiplatelet therapy through a randomized controlled design, in order to evaluate the risk of gastrointestinal injury and bleeding in CAD patients receiving different long-term antiplatelet therapy regimens after the implantation of contemporary DES, and provide clinical evidence for guidance of antiplatelet strategy after PCI.

## 1.1 Study Hypothesis

The principal hypothesis of this study is that following 6 months of DAPT, antiplatelet monotherapy with aspirin or clopidogrel between 6 and 12 months after DES implantation is superior to 12 months of DAPT for preventing gastrointestinal injury detected by AMCE.

## 1.2 Rationale of Study

1. Gastrointestinal mucosal injury (represented by erosion, ulcer, or bleeding) is a common complication of long-term antiplatelet therapy after PCI.
2. Trials in patients treated with contemporary DES have demonstrated that shortened DAPT regimens reduce the risk of major bleeding[1] with similar ischemic risk[2]. However, whether there is no definite evidence by current studies exploring the difference of gastrointestinal mucosal injuries among different antiplatelet strategies, mainly because gastroscopy, the most common detection method for gastrointestinal mucosal injury, have considerable limitations including invasive characteristic, requirement for discontinuation of APT and inability of detection of small intestine, so that can hardly be widely used for patients with PCI.
3. ANKON® magnetically controlled capsule endoscopy (AMCE) is a novel, noninvasive, actively and precisely controlled system which could visualize gastrointestinal injuries in the gamut of esophagus, stomach and small intestine (except colon). Patient acceptance of AMCE is higher than standard endoscopy as the procedure involves only swallowing a small capsule endoscope with non-invasive, painless, convenient and repeatable image observation. Discontinuation of antiplatelet drugs during AMCE is not necessary, which is substantially avoid the potential ischemic risk induced by discontinuation.

Therefore, our study first proposes that, 6-month DAPT plus 6-month antiplatelet monotherapy could significantly reduce the risk of gastrointestinal mucosal injury compared with 12-mongth DAPT after implantation of newer-generation DES, and first use AMCE as the method for detection of gastrointestinal mucosal injury among CAD patients with PCI, and establish a score system based on results of AMCE, to evaluate the risk of gastrointestinal injury and bleeding.

## 1.3 Benefit/risk and ethical assessment

The potential benefits that subjects might realize by participating in this randomized trial is a reduction in gastrointestinal mucosal injury for those who are randomly allocated to the experimental arm. Other benefits applicable to all subjects include the free antiplatelet medications after randomization, free inspections including AMCE examination for three times during the whole study period, fecal occult blood (FOB) and blood routine (BT) tests one time per two months, and close monitoring and surveillance for clinical events that will be performed by study personnel during the course of the trial.

The potential risks include an increased rate of thrombotic events for those who are randomized to the antiplatelet monotherapy arm and increased risk for bleeding in subjects randomized to the DAPT arm. However, based on current guidelines, increases of these potential risks are uncertain.

# 2. STUDY OBJECTIVES

## 2.1 Primary Objective

The primary objective of this study is to determine the risks of 12 months of DAPT vs 6 months of DAPT followed by 6 months of aspirin monotherapy or clopidogrel monotherapy on gastrointestinal mucosal injury after DES implantation.

## 2.2 Secondary Objective

The secondary objective is to evaluate the feasibility and safety of AMCE as a method for detecting gastrointestinal mucosal injury and bleeding in patients receiving APT.

## 2.3 Exploratory Objective

The exploratory objective is to establish a gastrointestinal mucosal injury scoring system that may identify patients at future risk for clinical gastrointestinal bleeding during long-term APT.

# 3. STUDY DESIGN AND FLOW CHART

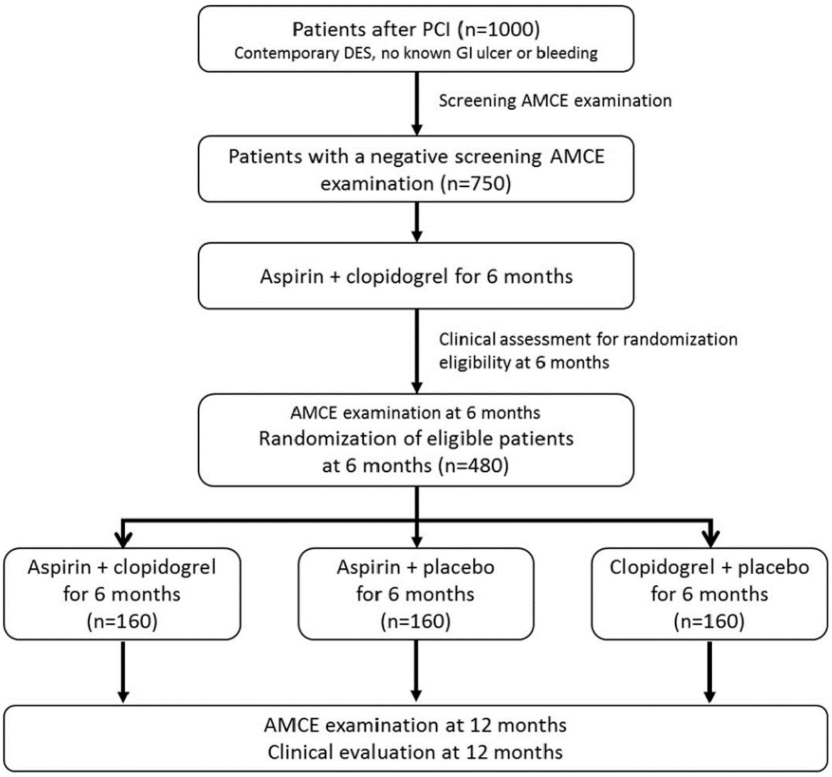


**Study flowchart.** PCI: percutaneous coronary intervention; DES, drug-eluting stent; GI: gastrointestinal; AMCE: ANKON magnetically controlled capsule endoscopy.

# 4. SUBJECT SELECTION CRITERIA

A subject is considered enrolled if all requisite inclusion and none of the exclusion criteria are met and upon provision of informed consent.

## 4.1 Inclusion criteria

1. Adult patients with age 18–80 years;
2. Presentation with stable angina, or non-ST-segment elevation acute coronary syndrome with GRACE score <140 at admission;
3. PCI with implantation of contemporary drug-eluting stent(s) during the present admission (contemporary DESs refer to drug-eluting stents with biodegradable polymer and (or) high biocompatibility and (or) thin cobalt-chromium or platinum-chromium struts. The current major DESs available in China market include: EXCEL (JW Medical System, Weihai, China), Tivoli (Essen Technology, Beijing, China), Endeavor Resolute (Medtronic Inc, Minnesota, USA),

FireHawk (MicroPort Medical (Group) Co, Ltd, Shanghai, China), Xience V (Abbott, Abbott Park, Illinois, USA), Xience Prime (Abbott Laboratories, Abbott Park, Illinois, USA), Promus Element and Synergy (Boston Scientific, Massachusetts, USA), Nano (Lepu Medical Technology CO., LTD, Beijing, China), etc.);

4. Complete revascularization (successful PCI treatment of all epicardial coronary lesions with diameter stenosis ≥70% or intermediate lesions with FFR <0.80);
5. Planned DAPT with aspirin and clopidogrel for at least 6 months;
6. Agreement to comply with all study procedures;
7. Written informed consent provided.

## 4.2 Exclusion criteria

1. Presentation with STEMI, or NSTE-ACS with GRACE score ≥140;
2. Left main disease (diameter stenosis >30%);
3. Any prior coronary stent implantation during the last year prior to the index procedure;
4. Implantation of first-generation drug-eluting stents or bioabsorbable scaffolds during the index procedure;
5. Implantation of >4 stents during the index procedure;
6. Any prior stent thrombosis;
7. Any active gastrointestinal bleeding or ulcers, or prior gastrointestinal bleeding or ulcers within the last 24 months;
8. Prior gastrointestinal tract or abdominal surgery other than simple procedures which would not change the gastrointestinal tract anatomy, such as polyp removal, cholecystectomy or appendectomy;
9. Contraindications to the AMCE test, including suspected or known gastrointestinal obstruction, stenosis, fistula, diverticula, etc.; presence of gastrointestinal obstruction symptoms such as pain or dysphagia; inoperative conditions or refusal to undergo abdominal surgery if required (because once the capsule could not pass the tract, a surgery may be needed);
10. Severe hemorrhoids (phase 3–4 according to guidelines of American Society of Colon and Rectal Surgery);
11. LVEF <0.40 on admission by echocardiography;

12. Renal dysfunction (eGFR <30 ml/min/1.73$m^2$);
13. Active hepatitis or ALT >3 times upper limits of normal at admission;
14. Severe hypertension (>180/110 mmHg), or hypertension without control;
15. Hemoglobin <100 g/L;
16. Platelet count <100 × $10^9$/L;
17. Unable to restrict use of PPI, gastric mucosa protectant or any other antacid agent by rule (details in “restrictions during the study”)
18. Required use of oral anticoagulation (warfarin or other factor II or factor X inhibitors);
19. Inability to take 12-month DAPT for any reason;
20. Mandatory use of >6-month DAPT (indicating those who are not suitable to receive aspirin or clopidogrel monotherapy);
21. Any comorbidity with estimated survival time <12 months (e.g., progressive cancer, chronic obstructive lung disease, etc.);
22. Any contraindication to MRI examination, including implantation of an MRI-incompatible pacemaker, defibrillator, or other ferromagnetic material, etc.;
23. Pregnant or plan to be pregnant;
24. Any condition that may interfere with any study procedures, such as dementia, immobility, alcohol use, etc.;
25. Planned surgery within 1 year;
26. Taking iron supplement;
27. Participating in any other clinical trial of an investigational drug or device that has not met its primary endpoint.

# 5. STUDY CONDUCT

## 5.1 Restrictions during the study

Enrolled subjects should remain on routine aspirin plus clopidogrel for the first 6 months after index PCI, and blinded study drug for the 6 months after randomization (the second 6 months after PCI).

Unless clinically indicated, PPIs or any other type of gastric mucosal protectants should not be taken by study participants during the course of the trial. Once these drugs are taken, it should not be administered continuously for more than 4 days or a total of more than 12 days before randomization (the first 6 months PCI); it should not be taken for more than 4 days continuously or in total of more than 10 days within 6 months after randomization. Otherwise, any condition that is not in accordance with restrictions above will be considered as a violation of the study protocol, and subjects with this condition will be suspended early from this study.

## 5.2 Screening before enrollment

After provided written consent, all patients (1000 patients) meeting the inclusion criteria and not meeting the exclusion criteria will receive an AMCE examination during 30-120 hours after PCI (gut purge is not allowed on the day of PCI) and a *Helicobacter Pylori* (HP) breath test, in order to identify the baseline level of gastrointestinal mucosal lesions and HP infection. Patients undergoing successful screening AMCE examination and with no ulceration or bleeding will be enrolled (at least 750 patients) and treated with open-label aspirin (100 mg/d) plus clopidogrel (75 mg/d) for 6 months. Additionally, blood routine test and fecal FOB test will be performed every 2 months after enrollment.

## 5.3 Subject randomization and initiation of investigational product

A second AMCE examination will be performed to evaluate eligibility for randomization in all enrolled patients at 6 months after PCI. Subjects with any one of items below should not be randomized:

1. Withdrawal of informed consent;
2. Lost to follow-up at 6 months;
3. Any event in the prior 6 months which in the opinion of the investigator results in the patient not being suitable for randomization of antiplatelet agent regimen either because of a) necessity to continue dual antiplatelet therapy (e.g., major adverse cardiovascular or cerebrovascular event within the prior 6 months or need for repeat stenting), or b) inability to continue dual antiplatelet therapy (e.g., bleeding, neoplasm, need for urgent surgery, etc. within the prior 6 months);
4. Not presently taking both aspirin and clopidogrel, or any prior temporary discontinuation of aspirin or clopidogrel for ≥5 days;
5. Proton pump inhibitors or any other kind of gastric mucosal protectant agents may not be used after study enrollment unless a clear clinical indication has developed (e.g., new gastrointestinal bleeding or ulcer disease). After

randomization, gastric mucosa protectants may not be taken for more than 4 days continuously or in total for ≥10 days without a clear clinical indication.

6. Unwillingness or inability to undergo the 6-month AMCE examination or the remainder of the study procedures, including the 12-month AMCE exam.

Patients successfully completing the 6-month follow-up AMCE exam and with no ulceration or bleeding (approximately 480 patients) are then randomly assigned in a 1:1:1 ratio to receive three antiplatelet strategies below: Group A, the standard DAPT (160 patients, aspirin plus clopidogrel); Group B, aspirin monotherapy (160 patients, aspirin plus clopidogrel-placebo); and Group C, clopidogrel monotherapy (160 patients, clopidogrel plus aspirin-placebo) for an additional 6 months in a double blinded manner. A third AMCE examination will be done at 12 months after enrollment.

After randomization, patients should also undergo blood routine test and FOB test every 2 months. Moreover, the first 102 enrolled patients (34 per group) from the site of PI (General Hospital of Northern Theatre Command) will undergo platelet function testing, including adenosine diphosphate-induced platelet aggregation by light transmission aggregometry and VerifyNow aspirin and P2Y12 testing assessment.

Patients who are ineligible for randomization will not receive any more study drugs, and subsequent antiplatelet therapy will be administered at their attending physicians' discretion in accordance with the local standard of care. All enrolled patients, irrespective of eligibility for randomization, should be followed up by telephone until 12 months.

## 5.4 Procedures for handling subjects incorrectly enrolled or randomized on investigational product

Subjects who are incorrectly enrolled will be immediately withdrawn from the study. The subject and the treating physician will be notified. If the subject has already been randomized, the study drug will be discontinued and further treatment will be per standard of care.

## 5.5 Blinding and procedures for unblinding the study

This study has a double-blind design with aspirin, clopidogrel and their corresponding placebos. Neither the subjects, study site research personnel, or treating physicians involved in the treatment or clinical evaluation of the subjects will be aware of treatments received. There will be an independent data safety monitoring board (DSMB) to monitor the data on a periodic basis.

## 5.5.1 Methods for ensuring blinding

This study has a double-blind design with aspirin, clopidogrel, and matching placebos. Shenyang Yu Kang Pharmaceutical Technology Co., Ltd, a statistical organization independent of the study, used SAS software to generate random codes for the corresponding groups (primary blind codes), and the study drug regimen corresponding to the group (secondary blind codes), and import the two levels of blind codes (including primary and secondary blind codes) via the Taimei Medical Randomization and Drug Management System (eBalance). Randomization is stratified according to site and will be performed in fixed block. The active tablets and the respective placebo tablets will be identical in size, color, smell, and taste. The patients, site personnel, sponsor personnel, persons performing the assessments, and data analysts will remain blinded to the identity of the treatment from the time of randomization until completing the statistical analysis. There will be an independent data safety monitoring board (DSMB) to monitor the data on a periodic basis.

### 5.5.2 Methods for breaking the blinding in the study

In the event of a medical emergency, in which knowledge of the investigational drug is critical to the subject's medical management, the blind for that subject may be broken by the treating physician. Before breaking the blinding, the consent of the research leader of the participating research site should be obtained and the relevant researchers and monitors should be informed. At the same time, the events leading to break the blinding and the blind code information should be recorded in detail. After breaking the blinding, the subject will not receive any study drug, and the treated physician can take corresponding bail-out measures according to local standard of care. The study drugs cannot be resumed even if the condition of subject is stable, while researchers still need to conduct clinical follow-up for 12 months for the subject with broken blinding, and AMCE examination is still required. Researchers should withdraw the study drugs from the patients whose blinding was broken.

### 5.5.3 Methods for unblinding in the study

Unblinding will be conducted after the whole study procedure. Because of the randomized, double-blinded, placebo-controlled study design, a two-level unblinding (first and second unblinding) is prespecified. The whole procedure of unblinding is organized by Data and Safety Monitoring Boards (DSMB), and collectively witnessed by representatives of research sites, contract research organization (CRO) and statistical organization. The unblinding should not be

performed until completing the statistical analysis.

The first unblinding: Only the definite group (such as group A, group B, and group C) of each subject is unblinded, but the study drug corresponding to each group is not clear yet. At this time, the information of study drug will be filed by statisticians for statistical analysis.

Second unblinding: After the statistical analysis is finished, a statistical report will be generated. The primary investigator will perform the second unblinding when publishing statistical results, and simultaneously announce the study drug of each group, then combine the blind codes to generate the final statistical results.

## 5.6 Treatments

### 5.6.1 Identity of investigational product

In this protocol, the investigational product is aspirin 100 mg tablets and matching aspirin-placebo tablets, and clopidogrel 75mg tablets and matching clopidogrel-placebo tablets.

**Table 1 Identity of Investigational Product**

| Study drug | The form and dosage of drug | Manufacturer |
|---|---|---|
| Bayaspirin (aspirin) | 100mg tablet | Bayer |
| Aspirin-placebo | tablet | Guangzhou Boji Medical Biotechnological co., Ltd. |
| Plavix (clopidogrel) | 75mg tablet | Sanofi-Aventis |
| Clopidogrel-placebo | tablet | Guangzhou Boji Medical Biotechnological co., Ltd. |

### 5.6.2 Doses and treatment regimens

After enrollment, patients will routinely administer study drugs (including open-label aspirin at a dose of 100mg swallowed with 100ml warm water 30 minutes daily before breakfast, plus open-label clopidogrel at a dose of 75mg daily) until the face-to-face study visit at 6 months. At the face-to-face study visit, randomized participants will be allocated study drugs (including: Group A, blinded-label aspirin at a dose of 100mg daily, plus blinded-label clopidogrel at dose of 75mg daily;

Group B, blinded-label aspirin at a dose of 100mg daily, blinded-label clopidogrel-placebo at dose of 75mg daily; Group C, blinded-label aspirin-placebo at a dose of 100mg daily, plus blinded-label clopidogrel at dose of 75mg daily) and should be used regularly until the end of study.

### 5.6.3 Rationale and procedures of AMCE

ANKON® magnetically controlled capsule endoscopy system (AMCE), which is provided by ANKON Medical Technologies (Shanghai, China) and ANKON Photoelectric Technology Co., Ltd (Wuhan, China, ANKON Enterprise), is composed of a magnetic navigation control system, a portable data recorder and a capsule position detector. The capsule endoscope (known as an endoscopic robot) has a length of 27 mm and a diameter of 11 mm and contains a permanent magnet. It provides a 140° viewing angle, a 30 mm depth of field and operates for at least 10 hours after ingestion. The activated capsule is swallowed into the digestive tract and continually records the condition of digestive tract mucosa. The dimensions of any visualized lesions are measured by the ANKON ESNavi software. After reaching the gastric cavity, the endoscopic capsule is navigated by the external magnetic control system to visualize all aspects of the stomach (the cardia, fundus, angulus, antrum, and pylorus). The controller allows movements of 2 mm and changes in viewing angle of 3°. AMCE is the first actively and accurately capsule gastroscope robot that could actively and precisely provide the whole gastric examination. Multicenter, large-scale randomized studies have demonstrated that the accuracy and specificity of AMCE system was high consistent with standard gastroscopy[3,4].

Preparation before AMCE examination: Patients fasted on food, colored liquid or syrup from 8 pm the day before AMCE examination. Gut purge shall be done 6-8 hours begore examination. On the following morning the subject is administered 10 ml of simethicone (Menarini Group, Florence, Italy) as a defoaming agent to clean the stomach cavity 40 minutes before the examination, and drinks water (500-1000 ml) until feeling stomach fullness. During examination, if the gastric cavity is not filled with sufficient liquid to enable navigation of the capsule, the subject will drink additional water.

After the whole stomach is examined, subjects continue to wear the portable recorder for visualization of the duodenum and small intestine. After examination of the whole stomach, subjects could leave the hospital but should continue to wear the portable recorder for examination of the small intestine. that Subjects should return to the hospital as soon as possible once the instrument suggests that the whole examination is finished, and are subsequently followed up for up to 2 weeks to determine if the capsule is ultimately excreted or adverse events occur. Subjects should record the time when the capsule is discharged with defecation. The capsule can be abandoned after elimination without recycling. Magnetic resonance imaging is prohibited before the confirmation of capsule excretion. If the capsule is not found to be excreted within 2 weeks after examination, the subject returns to the hospital

for detection of the capsule by a position detector or abdominal x-ray to confirm whether the capsule is still in the body. If it is, endoscopy may be performed to remove the capsule.

#### 5.6.4 Labeling

Each aspirin, clopidogrel or placebo bottle will be labeled in black ink. On this label the protocol number, blinded batch number, container number, blinded drug name, tablet quantity, storage conditions, directions for use and route of administration will be indicated.

At the study site, the investigational product should be stored in a secure area according to local regulations. It is the responsibility of the investigator to ensure that the investigational product is only dispensed to study subjects. The investigational product must be dispensed only from official study sites by authorized personnel according to local regulations.

The storage conditions for the subjects to follow will be included in the labeling on the bottles.

## 5.7 Concomitant and post-study treatment

There are no protocol specific concomitant treatments. After study completion, the physician will determine the ongoing medical treatment for each subject. These medications are open-label, and are available on their local sites (no more available for free).

## 5.8 Treatment compliance

Study drug compliance will be assessed using manual pill count at the 12-month in-person follow-up visit. Reconciliation between the quantity of shipped study drugs versus the allocation of study drugs via EDC and versus distribution of study drug to subject will be monitored and reconciled by investigators and trial monitors.

## 5.9 Discontinuation of study medication

### 5.9.1 Temporary discontinuation from study medication

In the event such as surgery or other invasive procedures that a subject is temporarily discontinued from study drugs or need a replacement of the blinded-label study drugs to open-label antiplatelet drugs <7 days according to local standard of care, subjects should be considered, where possible, for eligibility to resume blinded-label study drug under the judgement of treating physicians.

Details on the date, duration and cause of each temporary discontinuation of study drugs should be recorded in full in the appropriate section of the EDC.

### 5.9.2 Permanent discontinuation from study medication due to reasons below:

- Patient decision. The patient is at any time free to discontinue treatment, without prejudice to further treatment.
- Investigator's decision:

  1) Incorrectly enrolled patient in whom the inclusion/exclusion criteria violation would put the patient at undue risk.

  2) Investigators judge that due to adverse events related to any kind of study drug, continuous administration of this study drug would put the patient at undue risk.

Details on the date, duration and cause of each permanent discontinuation of study drugs should be fully recorded into the appropriate section of the EDC. When any of the study drugs are permanently discontinued, further antiplatelet therapy should be given to the subject according to attending physician's discretion. Any remaining study drugs should be returned to the local research site at the next scheduled follow-up visit. Although these subjects are no longer receiving any study drug, they should also be followed up until 12 months. Patients who permanently discontinue study drugs after randomization are still required to undergo AMCE examination, and investigators are required to withdraw study drugs from patients after discontinuation. Patients who permanently discontinue study drugs before randomization only needed to be followed up by telephone at12 months.

## 5.10 Withdrawal from study

Each enrolled subject shall remain in the trial until completion of the required follow-up period. However, a subject's participation in any clinical trial is voluntary and the subject has the right to withdraw at any time without penalty or loss of benefit. Conceivable reasons for discontinuation may include, but not be limited to, the following:

- Subject voluntary withdrawal
- Subject withdrawal by physician as clinically indicated
- Subject lost-to follow-up

The reason for subject discontinuation must be documented on the CRF and source documents. The principal investigators must also report all subject discontinuations to their Ethical Committee as defined by their sites' procedure. All data from

evaluations and treatments performed prior to the withdrawal should be documented on the CRFs. Source documents that pre-date the withdrawal should be submitted as required by the protocol. No data that post-dates the withdrawal will be collected.

Once a subject has withdrawn from the trial, no further follow-up contact will be performed. However, vital status may be obtained from public records. Medical therapy after stopping the study will be as prescribed by the subject's physician.

# 6. COLLECTION OF STUDY VARIABLES

## 6.1 Recording of data

The Investigator is responsible for maintaining complete and accurate documentation of the trial including but not limited to medical records, trial progress records, laboratory results, case report forms, signed informed consent forms, investigational product accountability records, correspondence with the EC or trial monitors, adverse event reports, and information regarding subject discontinuations.

The Investigator is required to maintain information in the subject's medical records which documents and corroborates data entered in the case report forms. As a minimum the subject record should contain:

- Medical history/physical exam documenting that subject meets inclusion/exclusion criteria
- Documentation of subject's consent and subject ID number in the trial
- Dated and signed notes from each subject visit
- Adverse events reported and their resolution or lack thereof including supporting documents such as hospital records, discharge summaries, catheterization reports, ECGs, etc.
- Record of protocol required medications during the trial
- Record of the subject's condition upon completion of or withdrawal from the trial

## 6.2 Data collection at enrollment and follow-up

Data collection commences after the subject has provided informed consent. Data collection including subject demographic information, laboratory tests, and procedural data, and AMCE examination data as well as follow-up visits or telephone contacts will be conducted by an Investigator or site coordinator who has been trained on the

protocol and Case Report Forms (CRF).

Data required for analysis will be obtained as outlined in Table 2.

**Table 2 schedule of data collection**

| | **Screening for Enrollment** | **Standard DAPT and randomization** | **After randomization** |
|---|---|---|---|
| **Study procedures** | **After PCI** | **Until 6 months after PCI** | **Until 12 months after PCI** |
| Follow-Up | Visit 1 | Visit 2 | Visit 3 |
| Window Period | 0-7 days | ±7 days | ±14 days |
| Eligibility Criteria | X | | |
| Patient Informed Consent | X | | |
| Withdrawal | X | X | X |
| Medical History/ Demographics | X | | |
| Previous Medical History | X | | |
| Vital Signs | X | X | X |
| Height and Weight | X | | |
| Physical Examination | X | | |
| Cardiac Markers (including TnT or TNI/CK/CK-MB) | X | | |
| Blood Routine Test[1] (RBC/WBC/HGB/PLT) | X | X | X |
| Coagulation Function Test (PT/APTT/FIB) | X | | |
| Fecal Occult Blood Test[1] | X | X | X |
| Platelet Aggregation Test[2] (light transmission aggregometry and VerifyNow) | X | X | X |
| HP Breath Test | X | | |
| 12-Leads ECG | X | | |
| AMCE Examination | X | X | X |
| Drug Distribution/Recycling | X | X | X |
| Concomitant Medications | X | X | X |
| Adverse Events | X | X | X |

1: one time per two months; 2: 34 patients per group in the principal research site

6-month visit: Each enrolled subject will be followed with an in-clinic face-to-face visit at 6 months after enrollment. Screening should be conducted before the 6-month face-to-face visit, subjects who are not eligible for randomization will not be required to complete the 6-month face-to-face visit.

12-month visit: Randomized subjects will return for the final in-clinic additional in-clinic face-to-face visits at 12 months after enrollment. Vital status of subjects who

did not meet eligibility for randomization can be obtained from medical records or public records at 12 months after enrollment.

### 6.2.1 Enrollment procedures

For patients who meet all inclusion criteria and do not meet any exclusion criteria, the investigator or designee will:

- Obtain written informed consent
- Obtain a complete demographic data, medical history, physical inspection information (including blood pressure, pulse, height, weight and ECG), and lab values including hemoglobin and creatinine
- Conduct the initial AMCE examination to determine baseline characteristics of gastrointestinal mucosa
- Review concomitant medications taken within the last 30 days
- Instruct the subject to notify the investigator of any occurrence of adverse events
- Schedule next clinic visit at 6 months, and instruct patients to administer DAPT (aspirin plus clopidogrel) during the first 6 months after PCI

### 6.2.2 Face-to face follow-up procedure at 6 months

- Assess for adverse events may include, but not be limited to MI, stroke, revascularization, bleeding and ST
- Review concomitant medications with subject
- Counsel subject about importance of study drug compliance
- Assess for randomization eligibility – If applicable:
    - Randomize subjects via the Taimei Medical Randomization and Drug Management System (eBalance), and obtain container/kit number
    - Conduct the second AMCE examination to observe gastrointestinal mucosal lesions
    - Dispense study medications for next 6 months
    - Schedule next clinic visit at 12 months (for randomized subjects)
- Instruct the subject and/or caregiver to notify the investigator of any occurrence of adverse events

- Instruct the subject to return study drugs at next time of in-clinic face-to-face visit

### 6.2.3 Face-to face follow-up procedure at 12 months (for randomized subjects)

- Assess for adverse events may include, but not be limited to MI, stroke, revascularization, bleeding and ST
- Conduct the third AMCE examination to observe gastrointestinal mucosal lesions
- Review concomitant medications with subject
- Recycle and count all of remaining study drugs

## 6.3 Efficacy and safety variables

The study site research personnel will collect data from the subjects during the follow-up contact for identification of the efficacy and safety variables, defined as the first occurrence of gastrointestinal mucosal injury (including erosion, ulceration and bleeding) detected by AMCE examination (efficacy variable), MACCE (including cardiac death, target lesion MI, ischemic stroke, and clinically-driven target lesion revascularization (safety variables), and GI related symptoms or signs such as abdominal distension, nausea, vomiting, or capsule retention (safety variables).

# 7. SAFETY

The Principal Investigator at each participating study site is responsible for ensuring that all staff involved in the study is familiar with the content of this section.

## 7.1 Definition of adverse events

An adverse event is the development of an undesirable medical condition or the deterioration of a pre-existing medical condition following or during exposure to a pharmaceutical product, whether or not considered causally related to the product. An undesirable medical condition can be symptoms (e.g., nausea, chest pain), signs (e.g., tachycardia, enlarged liver) or the abnormal results of an investigation (e.g., laboratory findings, ECG). In clinical studies, an AE can include an undesirable medical condition occurring at any time, including run-in or washout periods, even if no study treatment has been administered. The term AE is used to include both serious and non-serious AEs.

## 7.2 Definitions of serious adverse events

A serious adverse event is an AE occurring during any study phase (i.e., run-in, treatment, wash-out, follow-up), that fulfills one or more of the following criteria:

- Results in death
- Is immediately life-threatening
- Requires in-patient hospitalization or prolongation of existing hospitalization
- Results in persistent or significant disability/incapacity or substantial disruption of the ability to conduct normal life functions
- Results in a congenital abnormality or birth defect
- Is an important medical event that may jeopardize the subject or may require medical intervention to prevent one of the outcomes listed above.

The severity and causality of SAEs (their relationship to all study treatments and/or procedures) will be assessed by the investigator(s) and reported to the EC.

**Definition of suspected unexpected serious adverse reactions (SUSARs)**

A SUSAR is a Suspected Unexpected Serious Adverse Reaction. To qualify as a SUSAR, the event shall be:

- Serious adverse event
- Adverse reactions to study drugs
- Unexpected reaction – defined as the adverse reaction not regarded as a potential risk or an adverse drug reaction by study protocol, informed written consent or literature.

## 7.3 Recording of adverse events or serious adverse event

### 7.3.1 Time period for collection of adverse events

All AEs related to cardiovascular, cerebrovascular and gastrointestinal aspects and all SAEs should be collected from the time the subject signs the informed consent through study exit.

### 7.3.2 Follow-up of unresolved adverse events

For ALL SAEs the subject’s course must be monitored until the event has subsided or,

in a case of permanent impairment, until the event stabilizes and the overall clinical outcome has been ascertained.

### 7.3.3 Information to be collected for each AE/SAE

- Description of AE/SAE
- The dates when the AE/SAE started and stopped
- Whether the AE/SAE is serious or not
- Time of reporting SAE
- Investigator causality rating against the investigational product
- Action taken with regard to investigational product
- Whether the AE/SAE caused subject's withdrawal from study
- Outcome

**In addition, the following variables will be collected for SAEs:**

- Date the study site research personnel became aware of SAE
- Date when decision was made for meeting SAE criteria
- Criteria met leading to classification as SAE
- Date of hospitalization (if applicable)
- Date of discharge (if applicable)
- Date of death (if applicable)
- Probable cause of death (if applicable)
- Autopsy performed (if applicable)

It is important to distinguish between serious and severe AEs. Severity is a measure of intensity whereas seriousness is defined by the criteria in Section 7.2. An AE of severe intensity need not necessarily be considered serious. For example, nausea that persists for several hours may be considered severe nausea, but not an SAE. On the other hand, a stroke that results in only a limited degree of disability may be considered a mild stroke but would be an SAE.

### 7.3.4 Adverse Events based on signs and symptoms

When collecting AEs, the recording of diagnoses (when possible) is preferred to

recording a list of signs and symptoms. However, if a diagnosis is known and there are other signs or symptoms that are not generally part of the diagnosis, the diagnosis and each sign or symptom will be recorded separately.

### 7.3.5 Adverse Events based on examinations and tests

Deterioration as compared to baseline in laboratory values or vital signs should therefore only be reported as AEs if they fulfill any of the SAE criteria or are the reason for discontinuation of treatment with the investigational product. If deterioration in a laboratory value/vital sign is associated with clinical signs and symptoms, the sign or symptom will be reported as an AE and the associated laboratory result/vital sign will be considered as additional information. Wherever possible the reporting investigator uses the clinical, rather than the laboratory term (e.g., anemia versus low hemoglobin value). In the absence of clinical signs or symptoms, clinically relevant deteriorations in nonmandated parameters should be reported as AE(s). Any new or aggravated clinically relevant abnormal medical finding at a physical examination as compared with the baseline assessment will be reported as an AE.

### 7.3.6 Disease progression or pre-existing conditions

Disease progression can be considered as a worsening of a subject's condition attributable to the disease for which the investigational product is being studied. It may be an increase in the severity of the disease under study and/or increases in the symptoms of the disease. For example, persistent angina should be considered as disease progression and not an AE. Planned hospitalization for a pre-existing condition without serious deterioration in health, is not considered a serious adverse event.

### 7.3.7 Reporting of adverse events

AE should be reported to the EC of the participating research site for record within 2 working days after detection by the relevant personnel of the research site. SAE should be immediately reported to EC of this site, the sponsor, and the national and provincial Food and Drug Administration within 24 hours after detection by the relevant personnel of the research site.

# 8. ETHICAL AND REGULATORY REQUIREMENTS

## 8.1 Ethical conduct of the study

The trial will be conducted in compliance with the protocol, Good Clinical Practice

guidelines, and World Medical Association Declaration of Helsinki: Ethical Principles for Medical Research Involving Human Subjects as well as local regulations, and applicable regional regulatory requirements.

The clinical investigation shall not begin until the required approvals/favorable opinions from the respective regulatory authority and ethics committee have been obtained. Any additional requirements imposed by the respective regulatory authority and/or ethics committee will also be followed, where specified.

## 8.2 Ethics and regulatory review

The principal investigator in each study site shall obtain the approval of the protocol, informed consent and other trial related documents from the EC before participating in the study.

In accordance with the investigational site EC requirements, the Investigator will:

3. obtain written EC approval at predetermined time points to continue the trial;
4. submit any amendments to the protocol as well as associated informed consent form changes and obtain written EC approval obtained prior to implementation.

## 8.3 Informed consent

All subjects must provide written informed consent in accordance with the site's EC, using an EC-approved informed consent form. All subjects are to be fully informed and trial conduct must be in accordance to the World Medical Association Declaration of Helsinki: Ethical Principles for Medical Research Involving Human Subjects.

Protocol-specific procedures or alterations of patient care must not be performed until the prospective subject has provided a signed informed consent. The informed consent will be in the prospective subject's native language and will contain non-technical language to describe the investigational procedures. The informed consent form should also include a clause that ensures important new information will be provided to the subject throughout the clinical investigation.

After a review of the prospective subject's medical records to determine general eligibility, the investigator or authorized designee who has been trained on the protocol, will approach the prospective subject to explain the purpose and scope of the clinical trial, prospective risks, and benefits of participation. The prospective subject must be given the opportunity to ask questions about the trial and must be given sufficient time to decide to participate in the trial or not. Additional information requested by the prospective subject should be provided. Any coercion or undue improper influence on the prospective subject is to be avoided.

If the prospective subject agrees to participate, the informed consent form must be signed and personally dated by the prospective subject. The investigator or an authorized member of the research team who has witnessed the prospective subject's signature must also sign and date the informed consent, prior to enrollment of the prospective subject. A copy of the completed informed consent form must be provided to the subject. Local EC regulations regarding obtaining informed consent must be followed.

The subject's medical record should have a notation regarding the signing of the informed consent. The subject is to be made aware that their participation in the trial is voluntary, their legal rights will not be waived, and that they may withdraw from the trial at any time, without giving specific reason for doing so. The subject must also be informed that withdrawal from the trial will not affect their future treatment. The investigator is responsible for the achievement of written consent from the prospective subject before they are included in the trial. All subjects must provide informed consent in accordance with the local EC requirements, using an EC-approved informed consent form.

## 8.4 Changes to the protocol and informed consent form

If the protocol or informed consent form (ICF) needs an amendment, the principal research site is required to submit such amendment to the Regulatory Agencies and/or other regulating body in each participating country for approval. Approved protocol or ICF amendments will be provided to the investigators by the principal research site prior to implementing the amendment.

For administrative changes, the principal investigator is responsible for notifying the EC.

## 8.5 Deviations from protocol

### 8.5.1 Compliance to protocol

No investigative procedures other than those defined in this clinical investigational plan will be undertaken on the enrolled subjects without the written agreement of the EC and the principal research site. It is the Investigator's responsibility to ensure that there are no deviations from the clinical investigational plan and full compliance with all established procedures of the EC is maintained. The Investigator will not deviate from the clinical investigational plan for any reason except in cases of medical emergencies, when the deviation is necessary to protect the life or physical well-being of the subject.

### 8.5.2 Procedures for recording, reporting, and analysing protocol deviations

A deviation is an instance(s) of failure to follow, intentionally or unintentionally, the requirements of the protocol. All deviations must be reported to the principal research site. The occurrence of clinical investigational plan deviations will be monitored by the designee of participating research sites and trial monitors. It is the Investigators' responsibility to inform their EC of clinical investigational plan deviations in accordance with their specific EC reporting policies and procedures. In the event that an investigative site does not comply with the Investigator Agreement or clinical investigational plan, the principal research site will notify the investigator of the site's non-compliance.

## 8.6 Audits and inspections

In the event that an investigator is contacted by a Regulatory Agency in relation to this trial, the investigator will notify the site immediately. The investigator and study site research personnel must be available to respond to reasonable requests and inspection queries made during the inspection process. The investigator must provide the principal research site with copies of all correspondence that may affect the review of this trial. The principal research site will provide any needed assistance in response to regulatory inspections. For revision about management and safety of subject, it is the principal investigator's responsibility to obtain the approved protocol or ICF amendments from EC.

The EC's approval of the study protocol or ICF amendment above shall be written documented before implementation.

# 9. STUDY MANAGEMENT

## 9.1 Training

### 9.1.1 Training of Monitors

The monitors or designee will be trained to the protocol, randomization instructions, electronic case report forms, and study drug usage. The principal research site is responsible for the training.

### 9.1.2 Training of study site research personnel

Participating investigators and study site research personnel will be trained during site initiation visits collectively conducted by the representatives designed by The General

Hospital of Northern Theatre Command, the Department of Gastroenterology of Changhai Hospital and the ANKON Medical Technologies (Shanghai, China). All training must be documented and must include or reference the revision of materials used for training, who was trained, the trainer and date of training. Original training records should be maintained at the site in the Regulatory Binder and copies should be dispensed to the principal research site.

Site initiation/training involves a didactic session whereby the protocol, including screening procedures, clinical follow-up procedures, and study drug procedures are reviewed in detail along with investigator responsibilities.

## 9.2 Monitoring of the study

The trial monitors will monitor the trial over its duration according to the prespecified monitoring plan. The trial monitor will contact each site at appropriate intervals to review investigational data for accuracy and completeness and ensure compliance with the clinical investigation plan. The trial monitor may request all documents and required records that are maintained by the Investigator/Site, including medical records (office, clinic, or hospital) for the subjects in this trial. Source documentation must be available to substantiate proper informed consent procedures, adherence to protocol procedures, adequate reporting and follow-up of adverse events, accuracy of data collected on case report forms, and study drug information. The Investigator and/or study site research personnel will be available for monitoring contact. If a site visit is required, it is expected that the Investigator/Site will provide the trial monitor with a suitable working environment for review of study-related documents.

**Source data**

The investigator is responsible for maintaining complete and accurate documentation of the trial including but not limited to medical records, trial progress records, laboratory results, case report forms, signed informed consent forms, study drug accountability records, correspondence with the EC, the trial monitors, and the principal research site, as well as adverse event reports and information regarding subject discontinuations.

The investigator is required to maintain information in the subject’s medical records which documents and corroborates data entered in the case report forms. The investigator and the associated institution will permit direct access to source data and documents for study-related monitoring, audits, EC review, and regulatory inspections.

Subjects providing informed consent agree to allow the monitor or designee access and copying rights to pertinent information in their medical records concerning their participation in this trial. The investigator will obtain, as part of the informed consent, permission for trial monitors or regulatory authorities to review, in confidence, any

records identifying the subjects in this trial. This information may be shared with regulatory agencies; however, the monitor undertakes not to otherwise release the patient's personal and private information.

### 9.3 Study timetable and end of study

The trial is estimated to commence May 2017. The last subject follow-up at 12 months post-procedure is expected to occur in May 2019. The total expected duration of the trial is 24 months.

# 10. DATA MANAGEMENT

A computerized data entry and management system will be developed by Taimei Medical Randomization and Drug Management System. A closed and password protected data entry system has been designed to ensure that only the responsible data entry person and the Data Management site supervisor can enter and/or edit data and this can be done only by using the programs and/or utilities available on the menu system. An audit trail will be created by date/time and user stamping. Range checks, review screens, and various error trapping routines are built into the system as quality control procedures. All possible relevant information on the forms is pre-coded. The statistical analysis of data on this study will be completed by an independent statistical organization, Shenyang Yu Kang Pharmaceutical Technology Co., Ltd.

# 11. EVALUATION AND CALCULATION OF VARIABLES

## 11.1 Primary Endpoint

The primary endpoint of this study is the gastric and small intestinal mucosal injury (a composite of erosion, ulceration or bleeding) occurring within 12 months after enrollment.

Situations below can be regarded as valid primary endpoint results, including:

- Successful completion of the AMCE examination at 6 months after randomization (i.e., 12 months after enrollment), regardless of the results of examination;
- AMCE or gastroscopy examination driven by the occurrence of gastrointestinal bleeding at any time during the whole study period observes

new-onset gastric or small intestinal mucosal lesions.

## 11.2 Secondary Endpoint

1. The incidence and severity of gastric and intestinal mucosal lesions during the first 6 months after study enrollment (prior to randomization);
2. The incidence and severity of gastric and intestinal mucosal lesions after randomization (i.e., between 6 months and 12 months after study enrollment);
3. The incidence of clinically evident gastrointestinal hemorrhage attributed to the upper GI tract (or of unknown origin) during 6 months after study enrollment (prior to randomization);
4. The incidence of clinically evident gastrointestinal hemorrhage attributed to the upper GI tract (or of unknown origin) after randomization (i.e., between 6 months and 12 months after study enrollment);
5. The incidence of clinically evident gastrointestinal hemorrhage attributed to the upper GI tract (or of unknown origin) during 12 months after study enrollment;
6. Gastrointestinal symptoms (pain, nausea/vomiting, dysphagia, other) during the 12 months after enrollment;
7. All bleeding (BARC types 1–5) during the 12 months after enrollment;
8. The incidence of target lesion failure (TLF; cardiac death, target-vessel MI, or clinically-driven target lesion revascularization), during the 12 months after enrollment;
9. The incidence of net adverse clinical events (NACE, defined as TLF or BARC type 2–5 bleeding) during the 12 months after enrollment;
10. The incidence of stent thrombosis (ARC definite, probable, or definite/probable) during the 12 months after enrollment.

# 12. STATISTICAL METHODS AND SAMPLE SIZE DETERMINATION

The statistical analyses will be performed using SAS version 9.3.

## 12.1 Description of analysis sets

The Enrolled Population consists of all subjects who signed informed consent.

The full-analysis-set (FAS) population will consist of all subjects who have been randomized (i.e., when the subject number and allocated treatment are recorded in the EDC database) according to the intention-to-treat (ITT) principle. Subjects will be analyzed in the treatment group assigned by the EDC. The primary endpoint of gastric or intestinal mucosal injury will be analyzed in a modified intention to treat (mITT) population, (i.e., patients with valid primary endpoint results).

The per-protocol (PP) population will consist of all randomized subjects without any major deviations from the protocol. The following deviations will lead to exclusion from the PP population:

- Subjects not receiving the assigned treatment as allocated by the EDC or no treatment at all.
- Non-compliance to study drug. Non-compliance is defined as taking less than 80% of dispensed tablets based on manual pill bottle count at each study visit.
- Patients with overdue AMCE or gastroscopy examination at visit 3 (180 days ± 2 weeks after randomization).

### 12.1.1 Efficacy analysis set

All efficacy analyses will be performed on the FAS and PP population. The primary efficacy analysis will be performed on the FAS population. This analysis will be repeated in the PP population to support the primary results.

The primary efficacy endpoint gastric or small intestinal mucosal injury occurring within 12 months after enrollment, defined as a composite of erosion, ulceration or bleeding.

### 12.1.2 Safety analysis set

Safety analyses of study medications will be performed on the FAS population. The safety endpoint is MACCE defined as a composite of cardiac death, target lesion MI, ischemic stroke, clinical-driven TLR or stroke within 12 months after enrollment.

Safety analyses of AMCE examination will be performed in patients undergoing AMCE examination (including GI cleansing). The safety endpoints are digestive symptoms (abdominal distension, nausea, or vomiting, etc.) and retention of capsule endoscopy within 30 days.

## 12.2 Methods of statistical analyses

Continuous variables will be summarised as the number of observations, number of missing values, mean, standard deviation, median, quartiles, and range.

Categorical variables will be summarised as the number of observations, number of missing values, frequencies, and percentages.

Baseline clinical, demographic, laboratory and procedural characteristics will be summarized by randomized treatment group.

Demographic and baseline characteristics will be summarized by randomized treatment group and for all randomized subjects combined (i.e., FAS population). Baseline characteristics that will define subgroups of interest for the efficacy and safety analyses are:

- Age group (< 65, ≥ 65 years)
- Gender (Male, Female)
- Diabetes mellitus (yes, no)
- Chronic kidney disease (yes, no)
- CAD presentation (Stable, Unstable)
- HP infection status at enrollment (positive, negative)
- Antiplatelet treatment status at enrollment (>6 months, ≤6 month)

**Censoring:** Subjects not experiencing any endpoint will be censored at time of death, last contact date (for subjects who withdraw consent or are lost to follow-up) or 180-day ± 14 days after randomization, whichever comes first.

**Primary Efficacy Analysis:** The primary endpoint analysis will be conducted according to the intention-to-treat (ITT) principle. The main aim of this study is to determine whether the strategy of 6-month DAPT plus 6-month single antiplatelet therapy is superior to 12-month DAPT in terms of the primary endpoint (gastrointestinal injury) after contemporary DES implantation. The null hypothesis ($H_0$) for this analysis is that the incidence of primary endpoint in the experimental group is same as that of the control group, namely $P_0$=$P_1$. The alternative hypothesis ($H_1$) is that the incidences of primary endpoints in the two groups was not equal, namely $P_0 \neq P_1$, and the superiority test is conducted at the 2-sided significance level of 0.05. This analysis will be repeated in the Per-protocol (PP) population to support the main results.

The primary and secondary endpoints will be also analyzed in the following clinically

relevant pre-specified subgroups (Part 12.2). Formal interaction testing will be performed using the subgroup × treatment allocation as an additional term in the logistic models.

## 12.3 Determination of sample size

The cumulative incidence of the primary endpoint of gastric or small intestinal mucosal lesions within 12 months is estimated to be 47% in patients who received 12 months of DAPT and 30% in those treated with either aspirin or clopidogrel monotherapy beginning at 6 months after enrollment. With a 2:1 ratio in patients treated with either aspirin or clopidogrel monotherapy (the sum of aspirin monotherapy group and clopidogrel monotherapy group) after 6-month DAPT versus DAPT for 12 months, 384 evaluable patients (256 and 128 respectively) provide 90% power to detect a 17% absolute risk reduction (36% relative risk reduction) with a 2-sided type I error of 0.05. Assuming 20% loss of evaluable primary endpoint outcome assessments due to patient withdrawal, loss to follow-up between 6 and 12 months or suboptimal AMCE visualization of the GI tract at 12months, 480 patients are planned to be randomized. Assuming that an additional 10% of enrolled patients will not be randomized at 6 months because of adverse clinical events, non-compliance with antiplatelet therapy, lost to follow-up or withdrawal, 534 patients is required to be initially planned to be enrolled after baseline screening. Finally, assuming that 10% of patients who undergo a screening AMCE examination will be excluded due to unavailable valid image (or definite ulceration and active bleeding), therefore a total of 593 patients is initially planned to be consented and undergo the screening AMCE examination.

Among the first 200 patients enrolled, ~25% had gastrointestinal injury at baseline by screening AMCE (despite clinically absent bleeding or gastrointestinal complaints). Of those who passed the initial exam, only ~65% were eligible for randomization; 17% of patients were noncompliant with the 6-month repeat AMCE exam, and new gastrointestinal ulceration or bleeding was found on the 6-month AMCE examination in 18% of patients. The study sample size was adjusted accordingly so 1000 patients will be screened by AMCE at baseline, with 750 patients enrolled and followed to the 6-month randomization eligibility period to achieve the 480 patients randomized goal.

## 12.4 Clinical event committee

Clinical Events Committee (CEC) will be established, which is composed of a group of independent cardiologists and gastroenterologists not engaged in this study. It is CEC's responsibility to adjudicate all reported clinical events and categorize these events by definitions. If necessary, CEC will request source data from research sites. CEC members are unaware of which study drugs subjects are assigned. For event definitions see in Appendix 1, include:

- Gastrointestinal mucosal lesions (including erosion, ulceration and gastrointestinal bleeding)
- Gastrointestinal symptoms
- Death
- Recurrent MI
- Ischemia-driven target vessel revascularization
- Stent thrombosis
- Bleeding
- Other.

### 12.5 Data and safety monitoring board

The study will be conducted under the auspices of an independent Data and Safety Monitoring Board (DSMB). DSMB members will not have primary affiliation with the study sponsor, the EDC supplier or the principal investigator of the trial. Members of the Board will be determined prior to study enrollment.

All adverse events will be reported to the DSMB, and if necessary DSMB will ask for more information. DSMB will review data and determine reporting and stopping rules as specified in the DSMB charter. The DSMB members will review undisclosed temporary data, including adjudicated and non-adjudicated TLF, Bleeding, and other Serious Adverse Events and their incidence, in order to identify potential safety issues three months after random. On account of the result of the review, DSMB will provide a report about the safety of the study with the premise of not breaking the blind. Based on the safety data, the DSMB may recommend modifications to the protocol, suspension or termination of the trial, and advise the Executive Committee. Executive Committee should distribute the copy of safety report to all the participating centers. All final decisions, regarding trial modifications, however, rest with the Executive Committee.

## 13. IMPORTANT MEDICAL PROCEDURES TO BE FOLLOWED BY THE INVESTIGATOR

### 13.1 Overdose

If an overdose on the study drug occurs in the course of the study, then investigators

or other site personnel inform the study director, the principal investigator and the study monitor in this research site within one day, i.e., immediately but no later than the end of the next business day of when he or she becomes aware of it.

## 13.2 Pregnancy

Due to the study enrollment criteria and proposed population, women of child bearing potential (defined in Part 4.2) are excluded from participation in this study. In the unlikely event that pregnancy should occur during the course of the study, all outcomes of pregnancy should be reported to the study director, the principal investigator and the study monitor in this research site.

# 14. LIST OF REFERENCES

1. Zhang L, Li Y, Jing QM, et al. Dual antiplatelet therapy over 6 months increases the risk of bleeding after biodegradable polymer-coated sirolimus eluting stents implantation: insights from the CREATE study. J Interv Cardiol 2014;27:119-26.
2. Han Y, Xu B, Xu K, et al. Six Versus 12 Months of Dual Antiplatelet Therapy After Implantation of Biodegradable Polymer Sirolimus-Eluting Stent: Randomized Substudy of the I-LOVE-IT 2 Trial. Circ Cardiovasc Interv 2016;9:e003145.
3. Liao Z, Hou X, Lin-Hu EQ, et al. Accuracy of Magnetically Controlled Capsule Endoscopy, Compared With Conventional Gastroscopy, in Detection of Gastric Diseases. Clin Gastroenterol Hepatol 2016;14:1266-73 e1.
4. Zou WB, Hou XH, Xin L, et al. Magnetic-controlled capsule endoscopy vs. gastroscopy for gastric diseases: a two-center self-controlled comparative trial. Endoscopy 2015;47:525-8.
5. Malfertheiner P, Chan FK, McColl KE. Peptic ulcer disease. Lancet 2009;374:1449-61.
6. Kim BS, Li BT, Engel A, et al. Diagnosis of gastrointestinal bleeding: A practical guide for clinicians. World J Gastrointest Pathophysiol 2014;5:467-78.
7. Thygesen K, Alpert JS, Jaffe AS, et al. Third universal definition of myocardial infarction. J Am Coll Cardiol 2012;60:1581-98.
8. Cutlip DE, Windecker S, Mehran R, et al. Clinical end points in coronary stent trials: a case for standardized definitions. Circulation 2007;115:2344-51.
9. Lanza Fl Fau - Royer GL, Jr., Royer Gl Jr Fau - Nelson RS, Nelson Rs Fau - Chen TT, Chen Tt Fau - Seckman CE, Seckman Ce Fau - Rack MF, Rack MF. A comparative endoscopic evaluation of the damaging effects of nonsteroidal anti-inflammatory agents on the gastric and duodenal mucosa.
10. Scarpignato C, Dolak W, Lanas A, et al. Rifaximin Reduces the Number and Severity of Intestinal Lesions Associated With Use of Nonsteroidal Anti-Inflammatory Drugs in Humans. Gastroenterology 2017;152:980-2 e3.

# 15. APPENDIX 1- DEFINITION FOR STUDY ENDPOINTS

## 15.1 Definition of erosion and ulcer

Gastrointestinal erosion is defined as superficial mucosal breaks with a diameter of b5 mm. Gastrointestinal ulcer is defined as a mucosal break with a diameter ≥5 mm, typically covered with fibrin[5].

## 15.2 Definition and classification of gastrointestinal (GI) bleeding[6]

**Category 1:**

1) Hematemesis: refers to the patient's vomiting of blood due to acute bleeding in the upper gastrointestinal tract (esophagus, stomach, duodenum and jejunum after gastrojejunostomy, pancreas and biliary tract).

2) Hematochezia or melena: blood is discharged from the anus, and the color of the stool is bright red, dark red or tarry black, which is called hematochezia.

3) Positive fecal occult blood (FOB): a small amount of bleeding in the digestive tract, which cannot be confirmed by eyes or under a microscope. The erythrocytes are destroyed by digestion and there is no abnormal change in stool appearance.

**Category 2:**

1) Upper gastrointestinal bleeding: hemorrhage originating from the esophagus to the ligament of Treitz (located at the duodenojejunal flexure).

2) Lower gastrointestinal bleeding: bleeding that originates from a site distal to the ligament of Treitz.

## 15.3 The classification of internal hemorrhoids according to the guidelines of diagnosis and treatment of hemorrhoids of the American Society of Colon and Rectal Surgeons

I: Prominent hemorrhoidal vessels, no prolapse

II: Prolapse with Valsalva and spontaneous reduction

III: Prolapse with Valsalva requires manual reduction

IV: Chronically prolapsed manual reduction ineffective

## 15.4 Bleeding Academic Research Consortium definition of bleeding

### Table 3 Definition of BARC bleeding

| Type | Definition |
|---|---|
| 0 | No evidence of bleeding. |
| 1 | Bleeding that is not actionable and patient does not have unscheduled studies, hospitalization or treatment by a health care professional. |
| 2 | Any clinically overt sign of hemorrhage that is actionable but does not meet criteria for type 3, 4 or 5 bleeding. It must meet at least one of the following criteria:<br>1) requiring medical or percutaneous intervention guided by a health care profession, includes (but are not limited to) temporary/permanent cessation of a medication, coiling, compression, local injection;<br>2) leading to hospitalization or an increased level of care;<br>3) prompting evaluation defined as an unscheduled visit to a healthcare professional resulting in diagnostic testing (laboratory or imaging). |
| 3 | Clinical, laboratory and/or imaging evidence of bleeding with specific healthcare provider responses, as listed below: |
| 3a | 1) Any transfusion with overt bleeding;<br>2) Overt bleeding plus hemoglobin (Hb) drop ≥3 to <5g/dL * (provided Hb drop is related to bleeding). |
| 3b | 1) Overt bleeding plus Hb drop ≥5g/dL* (Hb drop is related to bleed);<br>2) Cardiac tamponade;<br>3) Bleeding requiring surgical intervention for control (excluding dental/nasal/skin/hemorrhoid);<br>4) Bleeding requiring intravenous vasoactive drugs. |
| 3c | 1) Intracranial hemorrhage (does not include microbleeds or hemorrhagic transformation; does include intraspinal). Subcategories: confirmed by autopsy, imaging or lumbar puncture;<br>2) Intraocular bleed compromising vision. |
| 4 | CABG – Related Bleeding<br>1) Perioperative intracranial bleeding within 48 hours;<br>2) Reoperation following closure of sternotomy for the purpose of controlling bleeding;<br>3) Transfusion of ≥5 units of whole blood or packed red blood cells within a 48-hour period;<br>4) Chest tube output ≥2L within a 24 hour period. |
| 5 | Fatal Bleeding. Bleeding directly causes death with no other explainable cause. Categorized further as either definite or probable. |
| 5a | Probable fatal bleeding is bleeding that is clinically suspicious as the cause of death, but the bleeding is not directly observed and there is no autopsy or confirmatory imaging. |
| 5b | Definite fatal bleeding is bleeding that is directly observed (either by clinical specimen – blood, emesis, stool, etc. – or by imaging) or confirmed on autopsy. |

## 15.5 Major Adverse Cardiovascular and Cerebrovascular Events

Major cardiovascular and cerebrovascular adverse events (MACCE) is death, recurrent MI, stroke and target vessel revascularization. The definitions of the individual components of MACE are given below.

### 15.5.1 Death

All-cause death comprises several subclassifications (Table 4). In general, all deaths are considered cardiac unless an alternate cause is unequivocally established, even among subjects with serious noncardiac comorbidities.

**Table 4 Classification of death**

| | |
|---|---|
| Cardiac death | Any death due to proximate cardiac cause (e.g., MI, low-output failure, fatal arrhythmia), unwitnessed death and death of unknown cause, and all procedure-related deaths, including those related to concomitant treatment, will be classified as cardiac death. |
| Vascular death | Death caused by noncoronary vascular causes, such as cerebrovascular disease, pulmonary embolism, ruptured aortic aneurysm, dissecting aneurysm, or other vascular diseases. |
| Nonvascular death | Any death not covered by the above definitions, such as death caused by infection, malignancy, sepsis, pulmonary causes, accident, suicide, or trauma. |

### 15.5.2 Myocardial infarction

According to the third universal definition of myocardial infarction[7], myocardial infarction is classified as follows:

- Type 1: Spontaneous myocardial infarction
- Type 2: Myocardial infarction secondary to an ischemic imbalance
- Type 3: Cardiac death infarction without available biomarker values due to myocardial infarction
- Type 4a: MI associated with PCI
- Type 4b: MI associated with stent thrombosis confirmed by angiography or at autopsy
- Type 5: MI associated with CABG.

Any of the following criterion meets the diagnosis of MI:

- Increased and/or decreased levels of cardiac biomarkers (preferred cardiac troponin) with at least one value above the 99th percentile upper reference limit (URL) plus at least one of the following:
  - Symptoms of ischemia;
  - New ST-T changes or new left bundle branch block (LBBB)
  - pathological Q waves
  - Imaging evidence of new loss of viable myocardium or new regional wall motion abnormality in a pattern consistent with an ischemic etiology;
  - Identification of a coronary thrombus by angiography or autopsy.
- Cardiac death with symptoms suggestive of myocardial ischemia and presumed new ischemic ECG changes or new LBBB, but death occurring before blood samples could be obtained, before cardiac biomarker could rise
- PCI-related MI is determined by the elevation of myocardial biomarkers:
  - The elevation of cTn values >5 x 99th percentile URL in patients with normal baseline values (99th percentile URL) or
  - The rise of cTn values >20% if the baseline values are elevated and are stable or falling.

In addition, meeting at least one of the following

- Symptoms suggestive of myocardial ischemia,
- New ischemic ECG changes or new LBBB,
- Angiographic loss of patency of a major coronary artery or a side branch or persistent slow or no-flow or embolization.
- Imaging demonstration of new loss of viable myocardium or new regional wall motion abnormality are required

### 15.5.3 Clinically-driven target vessel revascularization

Clinically-driven revascularization of target vessels includes the revascularization of the previously implanted vessels due to recurring or persistent ischemic symptoms. The revascularization is defined in accordance with the relationship with the target vessels[8].

## 15.6 Stent thrombosis[8]

Stent thrombosis is classified according to the level of certainty and timing following PCI (Table 5).

- Definite stent thrombosis: Angiographic or pathological confirmation of the presence of a thrombus that originates in the stent or in the segment 5 mm proximal or distal to the stent and presence of at least 1 of the following criteria within a 48-hour time window:
  - Acute onset of ischemic symptoms at rest
  - New ischemic ECG changes that suggest acute ischemia
  - Typical rise and fall in cardiac biomarkers (refer to definition of spontaneous MI)
- Probable stent thrombosis
  - Any unexplained death within the first 30 days
  - Irrespective of the time after the index procedure, any MI that is related to documented acute ischemia in the territory of the implanted stent without angiographic confirmation of stent thrombosis and in the absence of any other obvious cause
- Possible stent thrombosis
  - Clinical definition of possible stent thrombosis is considered to have occurred with any unexplained death from 30 days after intracoronary stenting until end of trial follow-up

**Table 5 The classification according to the timing of stent thrombosis**

| **Classification** | **Time of occurrence** |
|---|---|
| Acute stent thrombosis | 0 to 24 hours after stent implantation |
| Subacute stent thrombosis | >24 hours to 30 days after stent implantation |
| Late stent thrombosis | >30 days to 1 year after stent implantation |
| Very late stent thrombosis | >1 year after stent implantation |

# 16. APPENDIX 2 - MAGNETICALLY CONTROLLED CAPSULE ENDOSCOPY SCORING SYSTEM

## 16.1 The gastric mucosal injury is evaluated through the Lanza score[9]

### Table 6 Lanza score

| Endoscopic manifestation | score |
|---|---|
| No erosion | **0** |
| 1–2 erosions localized in the gastric antrum, body or bottom | **1** |
| 3–5 erosions localized in one area of the stomach | **2** |
| Erosions localized in 2 different areas of the stomach (total 6–9 lesions) | **3** |
| Gastric ulcer or ≥ 10 erosions | **4** |

Efficacy evaluation standard:
the proportion of mild gastric mucosal injury = cases with 1 point and 2 points / total cases × 100%
The proportion of severe gastric mucosal injury = cases with 3 points + 4 points / total cases × 100%

## 16.2 Five-point scoring system for small intestinal mucosal injury[10]

### Table 7 Five-point scoring system to assess intestinal mucosal injury

| Category | Score |
|---|---|
| Normal | **0** |
| Petechiae/red spot (demarcated, usually circular, area of crimson mucosa with preservation of villi) | **1** |
| Small number of erosions (1–4 erosions) | **2** |
| High number of erosion (>4 erosions) | **3** |
| Mucosal breaks (large erosion and/or ulcer) | **4** |

# 17. APPENDIX 3 – GASTROINTESTINAL SYMPTOM SCORE

Symptoms are categorized as four types: abdominal pain, bloating, acid reflux, and belching.

Each symptom includes a five-level scoring system (degree and frequency are scored separately; the maximum score = 4 + 4 = 8 points).

**Table 8 Five-point scoring system to assess gastrointestinal symptoms**

| Score | Symptom | Frequency |
|---|---|---|
| **0** | No symptom | |
| **1** | Mild: mild symptoms, need to pay attention to feel | once per week |
| **2** | Moderate: self-conscious obvious symptoms, but does not affect work and life | 2-3 times per week |
| **3** | Severe: self-conscious obvious symptoms, affect work and life | 4-5 times per week |
| **4** | Extremely severe: self-conscious very obvious symptoms, seriously affecting work and life | Almost daily or consistent |

# 18. APPENDIX 4 – TREATMENT PRINCIPLES OF GASTROINTESTINAL BLEEDING AND MUCOSAL INJURY

Regulation rules in Cardiology
DAPT should be discontinued immediately. After three days after stopping the bleeding by electronic gastroscopy:

3) If gastrointestinal injury is entirely controlled without reoccurrence of active bleeding, and hemoglobin is maintained at the level of ≥9 g/dL (90 g/L), clopidogrel monotherapy (75 mg, daily) should be firstly administered as soon as possible and aspirin (100 mg, daily) should be resumed at the 5$^{th}$ day after hemostasis. Times, types and doses of medications should be recorded in detail.
4) If active minor or occult bleeding still exists, or the level of hemoglobin fluctuates above 10 g/L in different days (monitor the blood routine test if necessary), only low molecular weight heparin (LMWH, hypo, daily) is used. Then at the 7$^{th}$ day after hemostasis, stop the LMWH and resume DAPT

(doses and frequencies as above).

## 18.1 Fecal Occult Blood (FOB) Tests

Precautions before testing:

1) Do not test during the period of menstrual bleeding, hemorrhoid bleeding or anal fissure;

2) To avoid affecting the judgment of FOB results, it is forbidden to take iron supplement, meat, liver, blood, and green vegetables within three days before the examination to avoid affecting the judgment of FOB results.

**Treatment of patients with positive results:**

**Before randomization:** For patients with positive FOB result, the test should be repeated for 2 consecutive days, and the complete blood count should be performed at the same time. If the FOB test is continuously positive with a decrease in hemoglobin (a drop of more than 10g/L compared to baseline screening), AMCE should be performed to confirm the lesion (for free), and even if no clear lesions are found, it should be considered that occult small intestinal bleeding exists. The patient will be treated according to the principle of gastrointestinal bleeding treatment, and is ineligible for study enrollment. If the continuously positive FOB result continues without hemoglobin reduction, the blood routine test should be monitored at the same time as each fecal occult blood test. If the hemoglobin drops more than 10g/L (compared with the first blood routine test after positive FOB result), AMCE should be performed to confirm the lesion (for free). Then the patient will be treated according to the principle of gastrointestinal bleeding treatment and is ineligible for study enrollment. If FOB test turns negative in a repeated test, no treatment will be given.

**After randomization:** For patients with positive FOB result, the test should be repeated for 2 consecutive days, and the complete blood count should be performed at the same time. If the FOB test is continuously positive with a decrease in hemoglobin (a drop of more than 10g/L compared to baseline screening), AMCE should be performed to confirm the lesion (for free), and even if no clear lesions are found, it should be considered that occult small intestinal bleeding exists. Breaking the blinding shall be performed immediately, and the patient will be treated according to the principle of gastrointestinal bleeding treatment. If the continuously positive FOB result continues without hemoglobin reduction, the blood routine test should be monitored at the same time as each fecal occult blood test. If the hemoglobin drops more than 10g/L (compared with the first blood routine test after positive FOB result), AMCE should be performed to confirm the lesion (for free), and even if no clear lesions are found, it should be considered that occult small intestinal bleeding exists.

Breaking the blinding shall be performed immediately, and the patient will be treated according to the principle of gastrointestinal bleeding treatment. The identification of time of the gastrointestinal bleeding event is the date of the first positive FOB result. If FOB test turns negative in a repeated test, no treatment will be given.

## 18.2 The treatment procedure of clinically-driven or AMCE-positive gastrointestinal (GI) bleeding

1) The patient with GI bleeding before randomization will be treated in accordance with the following (Appendix 1-3) rules. The patient will be no longer randomized and the study is terminated.

2) The patient with GI bleeding after randomization should be confirmed by gastroscopy, and endoscopic treatment is taken if necessary. If the lesion is not clear, the AMCE examination is performed to observe the small intestine lesions. If no clear bleeding is found, colonic bleeding is suspected, and further colonoscopy should be performed.

3) After obtaining the consent of principal of the participating research site, the treating physician could unblind for this patient (Part 5.4), conduct hemostatic treatment and adjust antiplatelet therapy according to the following rules (Appendix 1-3). Antiplatelet therapy could be at physician's discretion according to local standard care after curing the bleeding.

4) This patient will bot terminate study, but only clinical 12-month follow-up (without AMCE examination) is needed.

## 18.3 The treatment principle of clinically-driven or AMCE-positive gastrointestinal (GI) bleeding

### Gastroenterology treatment rules:

1) Patients with a definite diagnosis of GI bleeding should undergo endoscopic hemostasis as soon as possible with stable hemodynamics. The specific hemostasis methods can be implemented according to the experience of the endoscopy center of each hospital, such as hemostatic clips, APC, rinse with ice adrenal solution, heat probe, etc., plus PPI treatment. Medications: for gastric ulcer, PPI + gastric mucosal protective agent for 6 weeks, for duodenal ulcer, PPIs for 4 weeks. If patients with Helicobacter pylori (HP) infection requires eradication therapy, Quadruple therapy for 2 weeks could be performed.

2) Patients with GI bleeding and shock should be treated in accordance with routine treatment such as hemorrhagic shock monitoring, blood transfusion, fluid infusion, and hemostasis.

**Table 2 The specific procedures**

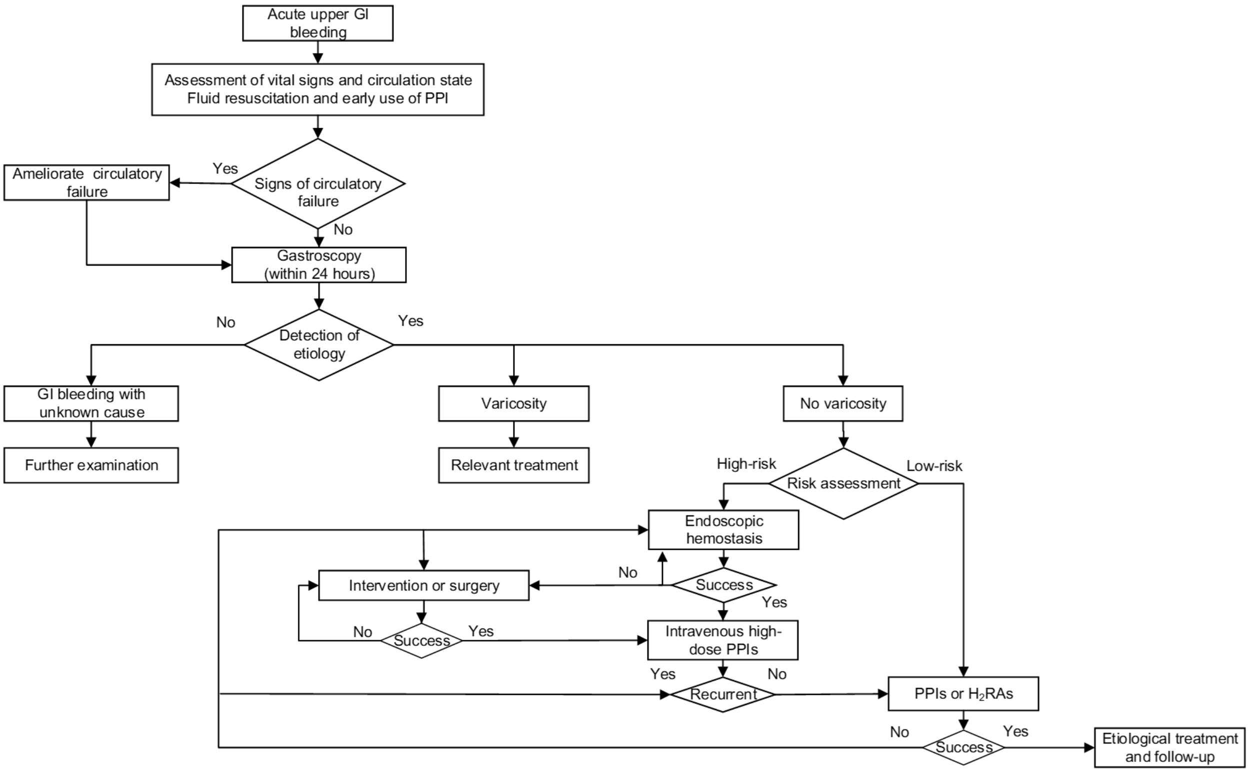


**Cardiology treatment rules:**

DAPT should be discontinued immediately. After three days after stopping the bleeding by electronic gastroscopy:

1) If gastrointestinal injury is entirely controlled without reoccurrence of active bleeding, and hemoglobin is maintained at the level of ≥9 g/dL (90 g/L), clopidogrel monotherapy (75 mg, daily) should be firstly administered as soon as possible and aspirin (100 mg, daily) should be resumed at the 5th day after hemostasis. Times, types and doses of medications should be recorded in detail.

2) If active minor or occult bleeding still exists, or the level of hemoglobin fluctuates above 10 g/L in different days (monitor the blood routine test if necessary), only low molecular weight heparin (LMWH, hypo, daily) is used. Then at the 7th day after hemostasis, stop the LMWH and resume DAPT (doses and frequencies as above).

## 18.4 Judgment criteria and treatment rules of severe gastrointestinal mucosal lesions requiring PPI

**Principles of PPI:** PPI and/or gastric mucosal protective agent should only be added to diagnosed ulcer lesions. No special treatment will be given if only an erosion exists.

**Treatment of peptic ulcer:**

Patients with diagnosed peptic ulcers should be treated with PPI. Gastric ulcer: PPI + gastric mucosal protective agent for 6 weeks, duodenal ulcer: PPI for 4 weeks.

For HP-positive patients, if requested by the patient, a quadruple therapy eradication treatment for 2 weeks could be given (PPI + bismuth + two kinds of antibiotics, PPI: omeprazole 20 mg bid; lansoprazole 30 mg, bid; Pantoprazole 40mg, bid; Rabeprazole 20mg, bid; Esomeprazole 20mg, bid; Bismuth: colloidal bismuth subcitrate 110mg 4 times per day; antibiotic: amoxicillin 1000mg, bid + clarithromycin 500mg, bid; amoxicillin 1000mg, bid + levofloxacin 500mg, qd; amoxicillin 1000mg, bid + furazolidone 100mg, bid; tetracycline 750mg, bid + furazolidone 100mg, bid; tetracycline 750mg, bid + metronidazole 400mg, bid or tid).

# Summary of OPT-PEACE Study Protocol Revision

| Section | Original description (V 1.3) | Revised description (V 1.4)<br><br>Revision date: 21-May-2018 | Justification |
|---|---|---|---|
| Title page | Comparison of mono-versus dual antiPlatelet Therapy during 6-12 months after new generation drug eluting stent implantation for Prevention of gastrointestinal injury Evaluated by Ankon magnetically controlled Capsule Endoscopy: a multicenter, randomized, double-blind, placebo-controlled study | Optimal antiplatelet therapy for prevention of gastrointestinal injury evaluated by ANKON magnetically controlled capsule endoscopy | Optimal antiplatelet therapy (OPT) is the prefix of series clinical trials sponsored by Shenyang Northern Theater Command. The full study name was changed accordingly. |
| Protocol synopsis | | Updated | |
| | Study centers: 26 sites in China | Study centers: 27 sites in China | The participating sites have been changed. |
| 3. study design and flowchart | | Study flowchart updated | |
| 4.2 exclusion criteria | 26. Participating in any other clinical trial of an investigational drug or device that has not met its primary endpoint. | **26. Taking iron supplement;**<br><br>27. Participating in any other clinical trial of an investigational drug or device that has not met | Due to the impact of iron supplement on the results of FOB test and the color of stools, taking iron supplement |

| | | | |
|---|---|---|---|
| | | its primary endpoint. | was additionally added into the exclusion criteria. |
| 5.2 Screening before enrollment | After provided written consent, all patients (593 patients) meeting the inclusion criteria and not meeting the exclusion criteria will receive an AMCE examination during 30-120 hours after PCI (gut purge is not allowed on the day of PCI) and a Helicobacter Pylori (HP) breath test, in order to identify the baseline level of gastrointestinal mucosal lesions and HP infection. Patients undergoing successful screening AMCE examination and with no ulceration or bleeding will be enrolled (at least 534 patients) and treated with open-label aspirin (100 mg/d) plus clopidogrel (75 mg/d) for 6 months. Additionally, blood routine test and fecal FOB test will be performed every 2 months after enrollment. | After provided written consent, all patients (**1000** patients) meeting the inclusion criteria and not meeting the exclusion criteria will receive an AMCE examination during 30-120 hours after PCI (gut purge is not allowed on the day of PCI) and a Helicobacter Pylori (HP) breath test, in order to identify the baseline level of gastrointestinal mucosal lesions and HP infection. Patients undergoing successful screening AMCE examination and with no ulceration or bleeding will be enrolled (at least **750** patients) and treated with open-label aspirin (100 mg/d) plus clopidogrel (75 mg/d) for 6 months. Additionally, blood routine test and fecal FOB test will be performed every 2 months after enrollment. | Description of screening and enrolment populations updated. |
| 12.3 Determination of sample size | Assuming that an additional 10% of enrolled patients will not be randomized at 6 months because of adverse clinical events, non-compliance with antiplatelet therapy, lost to follow-up or withdrawal, 534 patients is required | Assuming that an additional 10% of enrolled patients will not be randomized at 6 months because of adverse clinical events, non-compliance with antiplatelet therapy, lost to follow-up or withdrawal, 534 patients is | Sample size adjustment according to preliminary results. |

| | | | |
|---|---|---|---|
| | to be initially planned to be enrolled after baseline screening. Finally, assuming that 10% of patients who undergo a screening AMCE examination will be excluded due to unavailable valid image (or definite ulceration and active bleeding), therefore a total of 593 patients is planned to be consented and undergo the screening AMCE examination. | required to be initially planned to be enrolled after baseline screening. Finally, assuming that 10% of patients who undergo a screening AMCE examination will be excluded due to unavailable valid image (or definite ulceration and active bleeding), therefore a total of 593 patients is initially planned to be consented and undergo the screening AMCE examination.<br><br>**Among the first 200 patients enrolled, ~25% had gastrointestinal injury at baseline by screening AMCE (despite clinically absent bleeding or gastrointestinal complaints). Of those who passed the initial exam, only ~65% were eligible for randomization; 17% of patients were noncompliant with the 6-month repeat AMCE exam, and new gastrointestinal ulceration or bleeding was found on the 6-month AMCE examination in 18% of patients. The study sample size was adjusted accordingly so 1000 patients will be screened by AMCE at baseline, with 750 patients enrolled and followed to the 6-month randomization eligibility period to achieve the 480 patients randomized goal.** | |

# STATISTICAL ANALYSIS PLAN

## Optimal antiplatelet therapy for prevention of gastrointestinal injury evaluated by ANKON magnetically controlled capsule endoscopy

## (The OPT-PEACE Trial)

Version 1.1

30-May-2020

# CONTENTS

**ACRONYMS AND ABBREVIATIONS** ...................................................3

**1 INTRODUCTION** ...................................................5

*1.1 Preface* ...................................................5

**2 OBJECTIVES** ...................................................6

*2.1 Primary Objectives* ...................................................6
*2.2 Secondary Objectives* ...................................................6
*2.3 Exploratory Objectives* ...................................................6

**3 STUDY DESIGN** ...................................................6

*3.1 Overall study design and flow chart* ...................................................6
*3.2 Inclusion/Exclusion criteria* ...................................................8
*3.3 Subject randomization* ...................................................10
*3.4 Blinding and procedures for unblinding the study* ...................................................12
3.4.1 Methods for ensuring blinding ...................................................12
3.4.2 Methods for unblinding patients' treatment assignment ...................................................12
*3.5 Treatments* ...................................................13
3.5.1 Identity of Investigational Product ...................................................13
3.5.2 Doses and treatment regimens ...................................................13
*3.6 Study variables* ...................................................14
3.6.1 Study timetable and end of study ...................................................15

**4 PRIMARY AND SECONDARY VARIABLES** ...................................................16

*4.1 Primary variable* ...................................................16
*4.2 Secondary variables* ...................................................16
*4.3 Safety variables* ...................................................17

**5 SAMPLE SIZE** ...................................................18

**6 ANALYSIS SETS** ...................................................18

*6.1 Full analysis set (FAS)* ...................................................18
*6.2 Per protocol set (PPS)* ...................................................19
*6.3 Safety analysis set* ...................................................19

**7 SUBGROUPS** ...................................................20

**8 LOST TO FOLLOW-UP AND MISSING DATA** ...................................................21

**9 STATISTICAL METHODS** ...................................................22

*9.1 General principles* ...................................................22
*9.2 Analysis for the primary endpoint* ...................................................22
*9.3 Analysis for the secondary endpoints* ...................................................23
9.3.1 Analysis for categorical and ordinal endpoints ...................................................23
9.3.2 Analysis for time-to-event endpoints ...................................................24
*9.4 Safety analysis* ...................................................24
*9.5 Exploratory analysis* ...................................................25
*9.6 Subgroup analysis* ...................................................25

*9.7 Investigational medication compliance* .......................................................................26
*9.8 Sensitivity analyses* ..........................................................................................26
*9.9 pre-defined substudy* ..........................................................................................26
9.9.1 Platelet function......................................................................................................27
**10 SAMPLE SIZE RE-ESTIMATION** ................................................................................**27**
**11 TABLE LISTINGS**..........................................................................................................**27**
**12 REFERENCES** ..................................................................................................................**31**

# Acronyms and Abbreviations

| Acronym/Abbreviation | Term |
|---|---|
| AE | Adverse event |
| ALT | alanine aminotransferase |
| AMCE | ANKON® magnetically controlled capsule endoscopy |
| APT | Antiplatelet Therapy |
| ARC | Academic Research Consortium |
| BARC | Bleeding academic research consortium |
| CI | confidence interval |
| DAPT | Dual Antiplatelet Therapy |
| DES | Drug-eluting Stent |

| | |
|---|---|
| DSMB | data safety monitoring board |
| EDC | electronic data capture |
| eGFR | estimated Glomerular filtration rate |
| FAS | Full analysis set |
| GI | gastrointestinal |
| GRACE | Global Acute Coronary Event Registration |
| HP | Helicobacter pylori |
| HR | Hazard ratio |
| ITT | intent-to-treat |
| LVEF | Left ventricular ejection fraction |
| MACCE | major adverse cardiovascular and cerebrovascular event |
| MLS | The modified Lanza score |
| MRI | magnetic resonance imaging |
| NACE | net adverse clinical event |
| OPT-PEACE | Optimal antiplatelet therapy for prevention of gastrointestinal injury evaluated by ANKON magnetically controlled capsule endoscopy |
| OR | Odd ratio |
| PCI | Percutaneous Coronary Intervention |
| PPS | Per protocol set |
| SAE | serious adverse event |
| SS | safety set |
| STEMI | ST-segment elevation myocardial infarction |
| TLR | Target lesion revascularization |

# 1 Introduction

This document contains the Statistical Analysis Plan (SAP) for OPT-PEACE trial, protocol Version 1.4.

## 1.1 Preface

Dual antiplatelet therapy (DAPT) with aspirin and a P2Y12 inhibitor is the cornerstone for prevention of atherothrombosis after percutaneous coronary intervention (PCI) in patients with coronary artery disease. However, antiplatelet therapy (APT) may have serious adverse consequences, the most common of which is gastrointestinal mucosal injury with ulceration and bleeding. The frequency of gastrointestinal complications increases with increasing duration of DAPT. Trials in patients treated with contemporary drug-eluting stents (DES) have demonstrated that shortened DAPT regimens reduce the risk of major bleeding with (in most studies) similar ischemic risk. However, the optimal duration of DAPT remains controversial, and the true impact of prolonged DAPT on gastrointestinal injury is unknown. Although replacing DAPT with aspirin or clopidogrel monotherapy may prevent recurrent bleeding, in most prior studies major bleeding rates have been low and the absolute risk reductions of routine abbreviated DAPT regimens have been small.

Gastrointestinal injury (erosion, ulceration or subclinical bleeding) likely occurs with much greater incidence than overt bleeding, and may be a sensitive surrogate of antiplatelet agent safety. However, studies examining the risk of APT on gastrointestinal injury have not been performed due to the invasive nature of endoscopy. Moreover, gastroenterologists may decline to perform gastroscopy in patients on DAPT given the bleeding risk. Finally, upper endoscopy can only detect lesions in the stomach and duodenum, as it does not visualize the remainder of the small intestine. Thus, the extent to which a shortened DAPT strategy reduces primary gastrointestinal mucosal injury (with or without overt bleeding) and whether aspirin or clopidogrel monotherapy is safer are unknown.

ANKON® magnetically controlled capsule endoscopy (AMCE) is a minimally invasive, active controlled system capable of visualizing the stomach and entire small intestine. Patient acceptance of AMCE is higher than standard endoscopy as the

procedure involves only swallowing a small capsule endoscope. Discontinuation of antiplatelet drugs during AMCE is not necessary. Previous studies have confirmed that the sensitivity and specificity of AMCE for the detection of focal lesions of the gastrointestinal tract are similar compared with standard endoscopy.

# 2 Objectives

## 2.1 Primary Objectives

The primary study objective is to determine the risks of 12 months of DAPT vs a 6-month of DAPT followed by 6 months of aspirin monotherapy or clopidogrel monotherapy on gastrointestinal mucosal injury after DES implantation.

## 2.2 Secondary Objectives

The secondary objective is to evaluate the feasibility and safety of AMCE as a method for detecting gastrointestinal mucosal injury and bleeding in patients receiving APT.

## 2.3 Exploratory Objectives

The exploratory objective is to establish a gastrointestinal mucosal injury scoring system that may identify patients at future risk for clinical gastrointestinal bleeding during long-term APT.

For this purpose, 2 previously developed scoring systems will be used to assess the degree of mucosal injury observed by AMCE: (1) The modified Lanza score (MLS), and (2) a separate 5-point scoring system (See in protocol Appendix 2).

# 3 Study Design

## 3.1 Overall study design and flow chart

The OPT-PEACE is a multicenter, randomized, double-blind, placebo-controlled trial to evaluate the efficacy and safety of antiplatelet monotherapy with aspirin or clopidogrel between 6 and 12 months after DES implantation following 6 months of

DAPT detected by AMCE. All enrolled patients with a negative screening AMCE examination will be treated with open-label aspirin (100 mg/d) plus clopidogrel (75 mg/d) for 6 months. After 6 months, patients free from interval major adverse ischemic or clinically overt bleeding events and otherwise without any exclusion criteria (**section 3.2)** will undergo a second AMCE examination before randomization. Patients successfully completing this exam are then randomly assigned in a 1:1:1 ratio to receive aspirin plus clopidogrel, aspirin plus clopidogrel-placebo or clopidogrel plus aspirin-placebo for an additional 6 months in a double blinded manner.

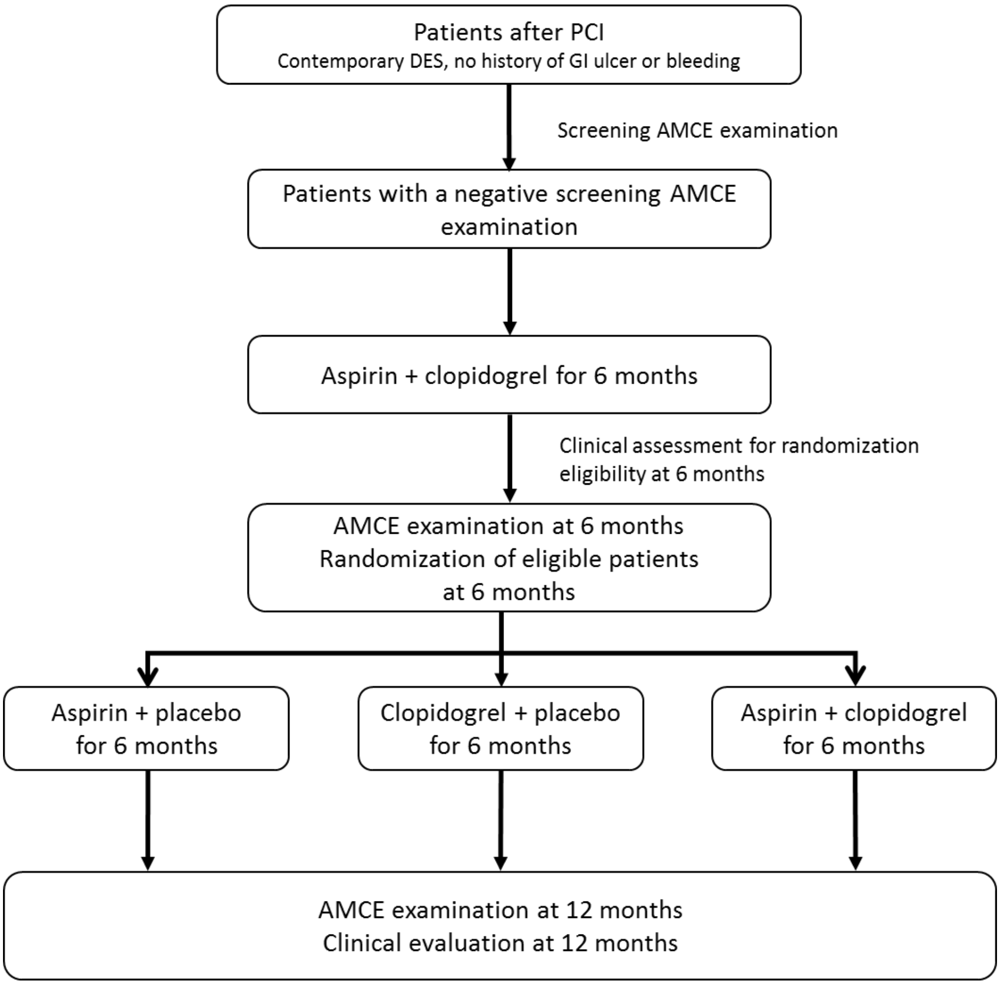


**Figure 1. Study flowchart of the OPT-PEACE trial**

## 3.2 Inclusion/Exclusion criteria

Patients with either stable coronary artery disease or low-risk acute coronary syndromes without ST-segment elevation (GRACE score<140) after complete coronary revascularization with at least 1 but not more than 4 contemporary DES will be eligible for the OPT-PEACE study.

Enrollment into the study will require meeting all inclusion AND none of the exclusion criteria. Patients meeting all entry criteria will provide informed written consent and then undergo a screening AMCE examination 30–120 hours after successful PCI. Only those without ulceration or bleeding by AMCE will be enrolled.

Inclusion criteria are listed below.

(1) Adult patients with age 18–80 years;

(2) Presentation with silent ischemia, stable angina, or non-ST-segment elevation acute coronary syndrome with GRACE score<140 at admission;

(3) PCI with implantation of contemporary DES(s) during the present admission.

*The contemporary DES refers to DES with thin cobalt-chromium or platinum-chromium struts, with a durable or biodegradable polymer eluting a rapamycin-analogue anti-proliferative agent. The current major DES available in China market include: EXCEL and EXCEL 2 (JW Medical System, Weihai, China), Tivoli(Essen Technology, Beijing, China), Endeavor Resolute (Medtronic Inc, Minnesota, USA), FireHawk (MicroPort Medical (Group) Co, Ltd, Shanghai, China), BuMA (SinoMedical, China), Xience V (Abbott, Abbott Park, Illinois, USA), Xience Prime (Abbott Laboratories, Abbott Park, Illinois, USA), Promus Element and Synergy (Boston Scientific, Massachusetts, USA)].*

(4) Complete revascularization (successful PCI treatment of all epicardial coronary lesions with diameter stenosis <70% or intermediate lesions with FFR≤0.80);

(5) Planned DAPT with aspirin and clopidogrel for at least 6 months;

(6) Agreement to comply with all study procedures;

(7) Written informed consent provided.

Meanwhile, patients should not enter the study if any of the following exclusion criteria are met:

(1) Presentation with STEMI;

(2) Left main disease (diameter stenosis>30%);

(3) Any prior coronary stent implantation during the last year prior to the index procedure;

(4) Implantation of first-generation drug-eluting stents or bioabsorbable scaffolds during the index procedure;

(5) Implantation of >4 stents during the index procedure;

(6) Any prior stent thrombosis;

(7) Any active gastrointestinal bleeding or ulcers, or prior gastrointestinal bleeding or ulcers within the last 24 months;

(8) Prior gastrointestinal tract or abdominal surgery other than simple procedures which would not change the gastrointestinal tract anatomy, such as polyp removal, cholecystectomy or appendectomy;

(9) Contraindications to the AMCE test, including suspected or known gastrointestinal obstruction, stenosis, fistula, diverticula, etc; presence of gastrointestinal obstruction symptoms such as pain or dysphagia; inoperative conditions or refusal to undergo abdominal surgery if required (ie, if the capsule will not pass and cannot be removed by endoscopy);

(10) Severe hemorrhoids (phase 3–4 according to guidelines of American Society of Colon and Rectal Surgery);

(11) LVEF<0.40 on admission by echocardiography;

(12) Renal dysfunction (eGFR<30 ml/min/1.73m$^2$);

(13) Active hepatitis or ALT >3 times upper limits of normal at admission;

(14) Uncontrolled severe hypertension (>180/110 mmHg);

(15) Hemoglobin<100 g/L;

(16) Platelet count<100 × 109/L;

(17) Planned use of a proton pump inhibitor, gastric mucosa protectant or any other antacid agent after study enrollment；

(18) Required use of oral anticoagulation (warfarin or other factor II or factor X inhibitors);

(19) Inability to take 12-month DAPT for any reason;

(20) Mandatory use of >6-month DAPT;

(21) Any comorbidity with estimated survival time<12 months (eg, progressive cancer, chronic obstructive lung disease, etc);

(22) Any contraindication to MRI examination, including implantation of an MRI-incompatible pacemaker, defibrillator, or other ferromagnetic material, etc;

(23) Pregnant or plan to be pregnant within 1 year;

(24) Any condition that may interfere with any study procedures, such as dementia, immobility, alcohol use, etc;

(25) Planned surgery within 1 year;

(26) Taking iron supplement;

(27) Participating in any other clinical trial of an investigational drug or device that has not met its primary endpoint.

## 3.3 Subject randomization

The eligibility for randomization for all enrolled patients will be evaluated at 6 months (±2 weeks), including medication adherence and the interval occurrence of adverse events. The patients with any of the following will not be randomized:

(1) Withdrawal of informed consent;

(2) Lost to follow-up at 6 months;

(3) Any event in the prior 6 months which in the opinion of the investigator results in the patient not being suitable for randomization of antiplatelet agent regimen either because of a) necessity to continue dual antiplatelet therapy (eg, major adverse cardiovascular or cerebrovascular event within the prior 6 months or need for repeat stenting), or b) inability to continue dual antiplatelet therapy (eg, bleeding, neoplasm, need for urgent surgery, etc within the prior 6 months);

(4) Not presently taking both aspirin and clopidogrel, or any prior temporary discontinuation of aspirin or clopidogrel for ≥5 days;

5) Use of proton pump inhibitors or gastric mucosal protectants for more than 12 days, or for more than 4 continuous days in the 6 months prior to randomization*;

(6) Unwillingness or inability to undergo the 6-month AMCE examination or the remainder of the study procedures, including the 12-month AMCE exam.

At this time patients free from interval major adverse ischemic or clinically overt bleeding events and otherwise without any above-mentioned exclusion criteria will undergo a second AMCE examination before randomization. Patients successfully completing 6-month follow-up AMCE exam and with no ulceration or bleeding are then randomly assigned in a 1:1:1 ratio to receive aspirin plus clopidogrel, aspirin plus clopidogrel-placebo or clopidogrel plus aspirin-placebo for an additional 6 months in a double blinded manner.

Randomization is stratified according to site and will be performed in fixed block of sizes 6 and block sizes will not be revealed to sites. The electronic data capture system (EDC) will be used to randomize subjects. The EDC will assign a treatment arm for patients and specify the appropriate medication number for the package of investigational treatment to be dispensed to the patient.

Enrolled patients who do not meet eligibility for randomization will not receive any more study drugs, and subsequent antiplatelet therapy will be administered at their attending physicians' discretion in accordance with the local standard of care. All

enrolled patients, irrespective of eligibility for randomization, should be followed up until 12 months.

## 3.4 Blinding and procedures for unblinding the study

### 3.4.1 Methods for ensuring blinding

This study has a double-blind design with aspirin, clopidogrel, and matching placebos. The active tablets and the respective placebo tablets will be identical in size, color, smell, and taste. The patients, site personnel, sponsor personnel, persons performing the assessments, and data analysts will remain blinded to the identity of the treatment from the time of randomization until completing the statistical analysis. There will be an independent data safety monitoring board (DSMB) to monitor the data on a periodic basis.

All AMCE images will be assessed by a core laboratory (Digestive Endoscopy Center, Department of Gastroenterology, Shanghai Changhai Hospital, Second Military Medical University, Shanghai, China) blinded to patient allocations.

### 3.4.2 Methods for unblinding patients’ treatment assignment

In emergency situations, in which knowledge of the investigational drug is critical to the subject’s medical management, the blind for that subject may be broken by the treating physician to provide optimal management. Before breaking the blinding, the consent of the research leader of the participating research site should be obtained and the relevant researchers and monitors should be informed. At the same time, the events leading to break the blinding and the blind code information should be recorded in detail. After breaking the blinding, the subject will not receive any study drug, and the treated physician can take corresponding bail-out measures according to local standard of care. The study drugs cannot be resumed even if the condition of subject is stable, while researchers still need to conduct clinical follow-up for 12 months for the subject with broken blinding, and AMCE examination is still required. Researchers should withdraw the study drugs from the patients whose blinding was broken.

# 3.5 Treatments

## 3.5.1 Identity of Investigational Product

The investigational product is enteric coated aspirin 100 mg tablets and matching aspirin-placebo tablets, and clopidogrel 75 mg and matching clopidogrel-placebo tablets.

**Table 1 Identity of Investigational Product**

| **Study drug** | **The form and dosage of drug** | **Manufacturer** |
|---|---|---|
| Bayaspirin (aspirin) | 100mg tablet | Bayer |
| Aspirin-placebo | tablet | Guangzhou Boji Medical Biotechnological co., Ltd. |
| Plavix (clopidogrel) | 75mg tablet | Sanofi-Aventis |
| Clopidogrel-placebo | tablet | Guangzhou Boji Medical Biotechnological co., Ltd. |

## 3.5.2 Doses and treatment regimens

After enrollment, patients will routinely administer study drugs (including open-label aspirin at a dose of 100mg swallowed with 100ml warm water 30 minutes daily before breakfast, plus open-label clopidogrel at a dose of 75mg daily) until the face-to-face study visit at 6 months. At the face-to-face study visit, randomized participants will be allocated study drugs (including: Group A, blinded-label aspirin at a dose of 100mg daily, plus blinded-label clopidogrel at dose of 75mg daily; Group B, blinded-label aspirin at a dose of 100mg daily, blinded-label clopidogrel-placebo at dose of 75mg daily; Group C, blinded-label aspirin-placebo at a dose of 100mg daily, plus blinded-label clopidogrel at dose of 75mg daily) and should be used regularly until the end of study.

# 3.6 Study variables

Data collection commences after the subject has provided informed consent. Data collection including subject demographic information, laboratory tests, and procedural data, AMCE examination data, as well as follow-up visits or telephone contacts will be conducted by an Investigator or site coordinator who has been trained on the protocol and Case Report Forms (CRF). Data required for analysis will be obtained as outlined in Table 2.

**Table 2 schedule of data collection**

| **Study procedures** | **Screening for Enrollment** | **Standard DAPT and randomization** | **After randomization** |
|---|---|---|---|
| | **After PCI** | **Until 6 months after PCI** | **Until 12 months after PCI** |
| Follow-Up | Visit 1 | Visit 2 | Visit 3 |
| Window Period | 0-7 days | ±7 days | ±14 days |
| Eligibility Criteria | X | | |
| Patient Informed Consent | X | | |
| Withdrawal | X | X | X |
| Medical History/ Demographics | X | | |
| Previous Medical History | X | | |
| Vital Signs | X | X | X |
| Height and Weight | X | | |
| Physical Examination | X | | |
| Cardiac Markers (including TnT or TNI/CK/CK-MB) | X | | |
| Blood Routine Test[1] (RBC/WBC/HGB/PLT) | X | X | X |

| | | | |
|---|---|---|---|
| Coagulation Function Test (PT/APTT/FIB) | X | | |
| Fecal Occult Blood Test[1] | X | X | X |
| Platelet Aggregation Test[2] (light transmission aggregometry and VerifyNow) | X | X | X |
| HP Breath Test | X | | |
| 12-Leads ECG | X | | |
| AMCE Examination | X | X | X |
| Drug Distribution/Recycling | X | X | X |
| Concomitant Medications | X | X | X |
| Adverse Events | X | X | X |

1: one time per two months; 2: 34 patients per group in the principal research site

6-month visit: Each enrolled subject will be followed with an in-clinic face-to-face visit at 6 months after enrollment. Screening should be conducted before the 6-month face-to-face visit, subjects who are not eligible for randomization will not be required to complete the 6-month face-to-face visit.

12-month visit: Randomized subjects will return for the final in-clinic additional in-clinic face-to-face visits at 12 months after enrollment. Vital status of subjects who did not meet eligibility for randomization can be obtained from medical records or public records at 12 months after enrollment.

### 3.6.1 Study timetable and end of study

Trial enrolment in OPT-PEACE began in July 2017 with the last patient enrolled on July 2019. The last patient follow-up visit will occur in Jun. 2020.

# 4 Primary and secondary variables

## 4.1 Primary variable

The primary endpoint is the incidence of gastric or intestinal mucosal injury occurring within 12 months after enrollment, defined as erosion, ulceration or bleeding detected by either planned AMCE or clinically-driven endoscopy. Specifically, gastrointestinal erosion is defined as superficial mucosal breaks with a diameter of <5 mm. Gastrointestinal ulcer is defined as a mucosal break with a diameter≥5 mm, typically covered with fibrin.

Situations below can be regarded as valid primary endpoint results, including:

- Successful completion of the AMCE examination at 6 months after randomization (i.e., 12 months after enrollment), regardless of the results of examination;
- Although the third AMCE examination at 6 months after randomization is not done, the second examination at 6 months after PCI reports new-onset gastric or small intestinal mucosal lesions;
- AMCE or gastroscopy examination driven by the occurrence of gastrointestinal bleeding at any time during the whole study period observes new-onset gastric or small intestinal mucosal lesions.

## 4.2 Secondary variables

Secondary endpoints listed below will be evaluated.

1. The incidence and severity of gastric and intestinal mucosal lesions (a composite of erosion, ulceration or bleeding) during the first 6 months after study enrollment (prior to randomization);

2. The incidence and severity of gastric and intestinal mucosal lesions (a composite of erosion, ulceration or bleeding) within 6 months after randomization (between 6 months and 12 months after study enrollment);

3. The incidence of clinically evident gastrointestinal hemorrhage attributed to the upper GI tract (or of unknown origin) during 6 months after study enrollment (prior to randomization);

4. The incidence of clinically evident gastrointestinal hemorrhage attributed to the upper GI tract (or of unknown origin) after randomization (ie, between 6 months and 12 months after study enrollment);

5. The incidence of clinically evident gastrointestinal hemorrhage attributed to the upper GI tract (or of unknown origin) during 12 months after study enrollment;

6. Gastrointestinal symptoms within 6 months after enrollment, within 6 months after randomization, and within 12 months after enrollment (details in Protocol Appendix 3);

7. All bleeding (BARC types 1–5) events within 6 months after enrollment, within 6 months after randomization, and within 12 months after enrollment;

8. The incidence of target lesion failure (TLF; cardiac death, target-vessel MI, or clinically-driven target lesion revascularization), during the 12 months after enrollment;

9. The incidence of net adverse clinical events (NACE, defined as TLF or BARC type 2–5 bleeding) within 6 months after enrollment, within 6 months after randomization, and within 12 months after enrollment;

10. The incidence of stent thrombosis (Academic Research Consortium defined definite/probable) within 6 months after enrollment, within 6 months after randomization, and within 12 months after enrollment.

## 4.3 Safety variables

Adverse events (AEs) of interest (i.e., abdominal distension, nausea, or vomiting after AMCE examination within 1 month) will be collected throughout the study period.

All serious adverse events (SAEs) will be recorded form the time of informed consent throughout the study.

Section 7 in the Protocol gives more detailed information on adverse event data collection.

# 5 Sample size

The cumulative incidence of the primary endpoint of gastric or intestinal mucosal lesions within 12 months is estimated to be 47% in patients who received 12 months of DAPT and 30% in those treated with either aspirin or clopidogrel monotherapy beginning at 6 months after enrollment. With a 2:1 ratio in patients treated with either aspirin or clopidogrel monotherapy after 6-month DAPT versus DAPT for 12 months, 384 evaluable patients (256 and 128 respectively) provide 90% power to detect a 17% absolute risk reduction (36% relative risk reduction) with a 2-sided type I error of 0.05. Assuming 20% loss of evaluable primary endpoint outcome assessments due to patient withdrawal, loss to follow-up between 6 and 12 months or suboptimal AMCE visualization of the GI tract at 12months, 480 patients are planned to be randomized. Assuming that an additional 10% of enrolled patients will not be randomized at 6 months because of adverse clinical events, noncompliance with antiplatelet therapy or lost to follow-up or withdrawal, 534 patients were initially planned to be enrolled after baseline screening. Finally, assuming that 10% of patients who undergo a screening AMCE examination will be excluded due to gastrointestinal ulcer or bleeding, approximately 593 patients were initially planned to be consented and undergo the screening AMCE examination.

# 6 Analysis sets

## 6.1 Full analysis set (FAS)

All patients who have been randomized to study treatment will be included irrespective of their protocol adherence and continued participation in the study. Patients will be analysed according to their randomized study drug assignment (not to which treatment they actually received) irrespective of whether the event occurred before or following discontinuation of study drug. Patients who withdraw consent to participate in the study (or are lost to follow-up) will be included up to the date of

their study termination except for vital status known through public records (for use in the analyses of deaths). All primary, secondary, and exploratory efficacy variables will be analyzed using the FAS. The primary endpoint of gastric or intestinal mucosal injury will be analyzed in a modified intention to treat (mITT) population, (i.e., patients with valid primary endpoint results). Unless otherwise stated, the FAS will be considered the primary analysis set for the primary and secondary variables and for the exploratory variables.

## 6.2 Per protocol set (PPS)

The PPS consists of all randomized subjects without any major deviations from the protocol. The following deviations in randomized subjects will lead to exclusion from the PP population. The primary analyses will be repeated in the PPS to support the primary results.

The following deviations will lead to exclusion from the PP population:

1. Subjects not receiving the assigned treatment as allocated by the EDC or no treatment at all;

2. Non-compliance to study drug. Non-compliance is defined as taking less than 80% of dispensed tablets based on manual pill bottle count at each study visit.

3. Patients with overdue AMCE or gastroscopy examination at visit 3 (180 days ± 2 weeks after randomization).

## 6.3 Safety analysis set

There are 2 safety sets (SSs) in OPT-PEACE trial. Safety and tolerability will be assessed from serious adverse events (SAEs) and adverse events (AEs). The assessment will include MACCE defined as a composite of cardiac death, target lesion MI, ischemic stroke, clinical-driven TLR or stroke in SS (investigational drug) and the incidence of AEs related to AMCE examination and capsule retention in SS (AMCE examination). All events will be independently adjudicated.

The first safety analysis set includes patients who received at least 1 dose of study drugs (aspirin, clopidogrel, and/or matching placebos) and who have data observed at any time after first randomized dose until the end of the study. The safety endpoint is MACCE defined as a composite of cardiac death, target lesion MI, ischemic stroke, clinical-driven TLR or stroke within 12 months after enrollment. Throughout the safety results sections, erroneously treated patients (patients randomized to one of the treatment groups but actually given the other treatment) will be accounted for in the actual treatment group. Patients with erroneous treatment would be analyzed according to that treatment only if they only received the erroneous treatment and none of the correct treatment.

The secondary safety analysis set includes patients who received AMCE exam at least once and observed up to 30 days for safety surveillance. The safety analysis set will be considered the primary analysis set for AMCE as a method for detecting gastrointestinal mucosal injury and bleeding. The safety endpoint is 1) adverse events, defined as symptoms or signs such as abdominal distension, nausea, or vomiting; 2) capsule retention (i.e., a capsule endoscope remaining in the digestive tract for a minimum of 2 weeks or a capsule endoscope that requires directed intervention or therapy to aid its passage).

All safety variables will be analyzed using the safety analysis set.

# 7 Subgroups

The primary and secondary endpoints will be also analyzed in the following clinically relevant pre-specified subgroups.

- Age (<65 years, ≥65 years)
- Sex (male, female)
- Diabetes mellitus (yes, no)
- Chronic kidney disease (eGFR<60, ≥60 mL/min/1.73m$^2$)
- Acute coronary syndrome (yes, no)

- Prior history of gastrointestinal bleeding (<24 months, ≥24 month)
- HP infection status (yes, no)*
- DAPT duration prior to randomization (>6 months, ≤6 months)

Another subgroup analysis will be performed in patients in whom follow-up was complete vs incomplete or otherwise affected by the COVID-19 pandemic (6-month visit after randomization date: before 2020/1/23, after 2020/1/23).

**A HP breath test will be performed after the screening AMCE exam to document HP infection status. HP eradication therapy is not mandatory but is allowed per physician discretion.*

# 8 Lost to follow-up and missing data

In keeping with an intent-to-treat philosophy, the primary analyses are performed on all randomized subjects. We anticipate that the amount of missing data will be minimal in the present study as we are specifically asking sites to ensure that the data elements are complete. No imputation will be carried out for missing baseline data.

If outcome data are missing, to assess the potential impact of missing data, a sensitivity analysis will be conducted which will include a complete case (including only subjects whose AMCE outcome is known at 6 months after randomization), a best-case (assume missing subjects in monotherapy group are event free at 6 months and missing subjects in DAPT have gastric or intestinal mucosal injury at 6 months), a worst-case (assume missing subjects in monotherapy group have gastric or intestinal mucosal injury at 6 months and missing subjects in DAPT group are event free at 6 months), and a tipping point analysis if the conclusion is changed in the worst-case analysis.

# 9 Statistical methods

## 9.1 General principles

Statistical analyses will be performed using SAS version 9.3. Unless otherwise stated, all hypothesis tests will be performed using two-sided tests at the 5% significance level. Data will be summarised overall and by treatment group. Continuous variables will be summarised as the number of observations, number of missing values, mean, standard deviation, median, quartiles, and range. Categorical variables will be summarised as the number of observations, number of missing values, frequencies, and percentages. Baseline clinical, demographic, laboratory and procedural characteristics will be summarized by randomized treatment group.

## 9.2 Analysis for the primary endpoint

The primary objective of OPT-PEACE trial is to determine the impact of 6-month DAPT plus 6-month single antiplatelet therapy versus 12-month DAPT in terms of the primary endpoint (gastrointestinal injury) after contemporary DES implantation. This analysis will be carried out in the FAS. The null hypothesis ($H_0$) for this analysis is that the incidence of primary endpoint in the experimental group is same as that of the control group, namely $P_0=P_1$. The alternative hypothesis ($H_1$) is that the incidences of primary endpoints in the two groups was not equal, namely $P_0 \neq P_1$, and the superiority test is conducted at the 2-sided significance level of 0.05. An odds ratio (OR) and two-sided 95% CI for the primary endpoint will be generated using the logistic regression model with treatment (monotherapy group vs. DAPT group) as fixed effect factors. This analysis will be repeated in the PPS to support the main results.

In addition, for the purpose of exploration, primary endpoint will also be compared across the 3 groups, and for all single group versus group comparisons (12-month DAPT vs 6-month DAPT plus 6-month aspirin monotherapy; 12-month DAPT vs 6-month DAPT plus 6-month clopidogrel monotherapy; and 6-month DAPT plus 6-month aspirin monotherapy versus 6-month DAPT plus 6-month clopidogrel monotherapy).

## 9.3 Analysis for the secondary endpoints

Analysis of the secondary endpoints will be based on the intention-to-treat principle by treatment group.

### 9.3.1 Analysis for categorical and ordinal endpoints

**Binary variables include:**

- The incidence and severity of gastric and intestinal mucosal lesions during the first 6 months after study enrollment (prior to randomization);
- The incidence and severity of gastric and intestinal mucosal lesions after randomization (ie, between 6 months and 12 months after study enrollment);
- The incidence of clinically evident gastrointestinal hemorrhage attributed to the upper GI tract (or of unknown origin) during 6 months after study enrollment (prior to randomization);
- The incidence of clinically evident gastrointestinal hemorrhage attributed to the upper GI tract (or of unknown origin) after randomization (ie, between 6 months and 12 months after study enrollment);
- The incidence of clinically evident gastrointestinal hemorrhage attributed to the upper GI tract (or of unknown origin) during 12 months after study enrolment.

Binary variables will be analyzed using logistic regression with treatment as fixed effect factors. Logistic regression model results will be reported as odds ratios for monotherapy group vs. DAPT group, with 95% CIs and p-values.

**Ordinal variables include:**

- Gastrointestinal symptoms (pain, nausea/vomiting, dysphagia, other) during the 12 months after enrolment (Protocol Appendix 3).

Ordinal outcomes will be compared between groups using proportional odds logistic regression models. Logistic regression model results will be reported as odds ratios for monotherapy group vs. DAPT group, with 95% CIs and p-values.

### 9.3.2 Analysis for time-to-event endpoints

Kaplan-Meier curves will be plotted by treatment group. Patients not experiencing a endpoint event during corresponding time interval will be censored at the time of death, last contact date (for subjects withdrawing consent or lost to follow-up) or 365 days, whichever comes first.

A hazard ratio (HR) and two-sided 95% CI for the primary endpoint will be generated using the Cox proportional hazards model. The null hypothesis (H0) for this analysis is that the HR = 1. The two-sided alternative hypothesis (HA) is that the HR ≠ 1. A test of superiority at the two-sided 0.05 level will be performed using a p-value from a log-rank test.

- All bleeding (BARC types 1–5) during the 12 months after enrollment;
- The incidence of target lesion failure (TLF); cardiac death, target-vessel MI, or clinically-driven target lesion revascularization), during the 12 months after enrollment;
- The incidence of net adverse clinical events (NACE, defined as TLF or BARC type 2–5 bleeding) during the 12 months after enrollment;
- The incidence of stent thrombosis (ARC definite, probable, or definite/probable) during the 12 months after enrollment.

In addition, all secondary endpoints will also be compared across the 3 groups, and for all single group versus group comparisons (12-month DAPT vs 6-month DAPT plus 6-month aspirin monotherapy; 12-month DAPT vs 6-month DAPT plus 6-month clopidogrel monotherapy; and 6-month DAPT plus 6-month aspirin monotherapy versus 6-month DAPT plus 6-month clopidogrel monotherapy).

All analysis will be repeated in the PPS to support the primary results.

## 9.4 Safety analysis

The safety evaluation will include MACCE and AEs, and SAEs related to AMCE exam. The analysis of MACCE will follow similar methodology as the analysis of the

time-to-event outcome (described in Section 9.3.2) based on the safety analysis set (investigational product). The analysis of AEs and SAEs related to AMCE examination will be summarised as for the safety analysis set (patients who received AMCE examination at least once) as a whole.

## 9.5 Exploratory analysis

The variables planned to be analyzed are provided in the exploratory objectives in section 2.3. Exploratory analyses will examine the predictive value of baseline clinical, demographic, laboratory, procedural characteristics, concomitant medication, and treatment allocation (if possible) as independent predictors of gastrointestinal mucosal injury at baseline, 6 months before randomization, and 12 months. Subsequently, establishing a gastrointestinal mucosal injury scoring system that may identify patients at future risk for clinical gastrointestinal bleeding during long-term antiplatelet therapy.

## 9.6 Subgroup analysis

The primary and secondary endpoints will be summarised in clinically relevant subgroups of the FAS as a whole and by treatment group. For each subgrouping variable, ORs and two-sided 95% CI will be calculated within each subgroup using a Logistic regression with treatment as fixed effect factors. Formal interaction testing on the ratio scale will be performed using the subgroup × treatment allocation as an additional term in the Logistic model.

The following subgrouping variables will be considered:

- Age (<65 years, ≥65 years)
- Sex (male, female)
- Diabetes mellitus (yes, no)
- Chronic kidney disease (eGFR<60, ≥60 mL/min/1.73m$^2$)
- Acute coronary syndrome (yes, no)

- Prior history of gastrointestinal bleeding (<24 months, ≥24 month)
- HP infection status (yes, no)
- DAPT duration prior to randomization (>6 months,≤6 months)

Another subgroup analysis will be performed in patients in whom follow-up was complete vs incomplete or otherwise affected by the COVID-19 pandemic (6-month visit after randomization date: before 2020/1/23, after 2020/1/23).

## 9.7 Investigational medication compliance

Compliance will be assessed by the investigator and/or study personnel at each visit using pill counts and information. Study drug accountability will be determined by the site monitor while performing routine site visits and at the completion of the study.

Study drug compliance will be assessed using manual pill count at the 12-month in-person follow-up visit. The percentage of study drug compliance for the overall treatment period will be derived for each patient based on pill counts as the number of pills taken (dispensed – returned), relative to the expected number of pills taken. Study drug compliance will be presented descriptively, including mean, median, quartiles and 5% and 95% percentiles.

*Non-compliance is defined as taking less than 80% of dispensed tablets based on manual pill bottle count. The non-compliance patients will exclude from the PP population.*

## 9.8 Sensitivity analyses

See SAP section 8.

## 9.9 Pre-defined substudy

The following sub-study is either embedded within the ongoing framework of OPT-PEACE. A general description of planned substudy is provided below.

### 9.9.1 Platelet function

A subset of patients will participate platelet function substudy. Platelet function testing, including adenosine diphosphate-induced platelet aggregation by light transmission aggregometry and VerifyNow aspirin and P2Y12 testing assessment, will be performed at baseline screening, randomization and at the end of the study in first 102 enrolled patients (34 per group) in the site of PI.

# 10 Sample size Re-estimation

Among the first 200 patients enrolled, ~25% had gastrointestinal injury at baseline by screening AMCE (despite clinically absent bleeding or gastrointestinal complaints). Of those who passed the initial exam, only ~65% were eligible for randomization; 17% of patients were noncompliant with the 6-month repeat AMCE exam, and new gastrointestinal ulceration or bleeding was found on the 6-month AMCE examination in 18% of patients. The study sample size was adjusted accordingly so 1000 patients will be screened by AMCE at baseline, with 750 patients enrolled and followed to the 6-month randomization eligibility period to achieve the 480 patients randomized goal.

# 11 Table Listings

**1. Enrolled Cohort**

1.1 Registration (Inclusion and Exclusion criteria)

1.1.1 Baseline demographics (age, sex, height, weight, et al.)

1.1.2 Baseline clinical and medical history (risk factors, pre-admission antiplatelet therapy, et al)

1.1.3 Baseline diagnoses

1.1.4 Baseline laboratory assessment

1.1.5 Baseline medications

1.1.6 Baseline procedure details

1.1.7 Discharge medications

1.1.8 Adverse events during baseline hospitalization (cardiovascular, gastric or intestinal, et al.)

1.1.9 Gastrointestinal mucosal injury scoring (MLS and a 5-point scoring system)

1.1.10 AMCE examination details

1.1.10.1 Binary frequency of gastrointestinal mucosal injury at baseline

1.1.10.2 Binary frequency of gastrointestinal mucosal injury at 6 months

1.1.10.3 Binary frequency of clinically evident gastrointestinal hemorrhage attributed to the upper gastrointestinal tract baseline

1.1.10.4 Binary frequency of clinically evident gastrointestinal hemorrhage attributed to the upper gastrointestinal tract at 6 months

1.1.11 Gastrointestinal symptoms assessment (pain, nausea/vomiting, dysphagia, et al)

1.1.11.1 Ordinal frequency of gastrointestinal symptoms assessment at baseline

1.1.11.2 Ordinal frequency of gastrointestinal symptoms assessment at 6 months

1.2.1 Site reported AEs/SAEs

1.2.2 Adjudicated AEs/SAEs

1.2.3 Mortality assessment

1.2.4 Medications adherence (DAPT, PPI, et al)

1.2.4 Subject disposition (lost or consent withdrawn)

1.2.5 Eligibility for randomization evaluation

1.2.5.1 Reasons for randomization ineligibility

**2. Randomized ITT Cohort**

2.1 Registration (Inclusion and Exclusion criteria)

2.1.1 Baseline demographics (age, sex, height, weight, et al.)

2.1.2 Baseline clinical and medical history (risk factors, pre-admission antiplatelet therapy, et al)

2.1.3 Baseline diagnoses

2.1.4 Baseline laboratory assessment

2.1.5 Baseline medications

2.1.6 Baseline procedure details

2.1.7 Discharge medications

2.1.8 Adverse events (cardiovascular, gastric or intestinal, et al.)

2.1.9 Gastrointestinal mucosal injury scoring (MLS and a 5-point scoring system at baseline, 6 months, and 12 months)

2.1.10 AMCE examination details (baseline, 6 months, and 12 months)

2.1.10.1 Binary frequency of gastrointestinal mucosal injury at 12 months

2.1.10.2 Binary frequency of clinically evident gastrointestinal hemorrhage attributed to the upper gastrointestinal tract at 12 months

2.1.11 Gastrointestinal symptoms assessment (pain, nausea/vomiting, dysphagia, et al)

2.1.11.1 Ordinal frequency of gastrointestinal symptoms assessment at baseline

2.1.11.2 Ordinal frequency of gastrointestinal symptoms assessment at 6 months

2.1.11.3 Ordinal frequency of gastrointestinal symptoms assessment at 12 months

2.2.1 Site reported AEs/SAEs

2.2.1.1 12 months binary frequency and KM estimates of TLF

2.2.1.2 12 months binary frequency and KM estimates of all bleeding

2.2.1.3 12 months binary frequency and KM estimates of NACE

2.2.1.4 12 months binary frequency and KM estimates of stent thrombosis

2.2.2 Adjudicated AEs/SAEs

2.2.2.1 12 months binary frequency and KM estimates of TLF

2.2.2.2 12 months binary frequency and KM estimates of all bleeding

2.2.2.3 12 months binary frequency and KM estimates of NACE

2.2.2.4 12 months binary frequency and KM estimates of stent thrombosis

2.2.3 Medications adherence (DAPT, PPI, et al)

2.2.4 Subject disposition (lost or consent withdrawn)

# 12 References

1. Han Y, Xu B, Xu K, et al.Six Versus 12 Months of Dual Antiplatelet Therapy After Implantation of Biodegradable Polymer Sirolimus-Eluting Stent: Randomized Substudy of the I-LOVE-IT 2 Trial. Circ Cardiovasc Interv. 2016 Feb;9(2):e003145.

2. Zhang L1, Li Y, Jing QM, et al. Dual antiplatelet therapy over 6 months increases the risk of bleeding after biodegradable polymer-coated sirolimus eluting stents implantation: insights from the CREATE study. J Interv Cardiol. 2014 Apr;27(2):119-26.

3. Zou WB, Hou XH, Xin L et al.Magnetic-controlled capsule endoscopy vs. gastroscopy for gastric diseases: a two-center self-controlled comparative trial.Endoscopy. 2015 Jun;47(6):525-8.

4. Liao Z, Hou X, Lin-Hu EQ et al.Accuracy of Magnetically Controlled Capsule Endoscopy, Compared With Conventional Gastroscopy, in Detection of Gastric Diseases.Clin Gastroenterol Hepatol. 2016 Sep;14(9):1266-1273.e1.

5. Peter Malfertheiner, Francis K L Chan, Kenneth E L McColl. Peptic ulcer disease. Lancet 2009; 374: 1449–61.

6. Kim BSM, Li BT, Engel A, Samra JS, Clarke S, Norton ID, Li AE. Diagnosis of gastrointestinal bleeding: A practical guide for clinicians. World J Gastrointest Pathophysiol 2014; 5(4): 467-478.

7. Thygesen K1, Alpert JS, Jaffe AS et al. Third universal definition of myocardial infarction. Eur Heart J. 2012 Oct;33(20):2551-67.

8. Cutlip DE, Windecker S, Mehran R, Boam A, Cohen DJ, van Es GA, et al. Clinical end points in coronary stent trials: a case for standardized definitions. Circulation. 2007 May 1;115(17):2344-51. PubMed PMID: 17470709.

9. Lanza FL, Royer Jr GL, Nelson RS, Chen TT, Seckman CE, Rack MF. A comparative endoscopic evaluation of the damaging effects of nonsteroidal anti-

inflammatory agents on the gastric and duodenal mucosa. Am J Gastroenterol. 1981;75(1):17–21.

10. Carmelo Scarpignato, Werner Dolak, Angel Lanas, et al. Reduces Number and Severity of Intestinal Lesions Associated With useof Non-steroidal Anti-inflammatory Drugs in Humans. Gastroenterology.2016 Dec 19. pii: S0016-5085(16)35504-4.
