# Supplementary material for: Progression of Gastrointestinal Injury During Antiplatelet Therapy After Percutaneous Coronary Intervention: A Secondary Analysis of the OPT-PEACE Randomized Clinical Trial
Source: JAMA Netw Open. 2023 Nov 17;6(11):e2343219. doi: 10.1001/jamanetworkopen.2023.43219 (PMC10656648; doi:10.1001/jamanetworkopen.2023.43219)
Supplement: Supplement 2. — eTable 1. Antiplatelet Medication Use at Baseline and During 12-Month Follow-Up of the Modified Intention-to-Treat Population eTable 2. Incidence of Gastric Injury and Small-Intestinal Injury at Baseline of the Modified Intention-to-Treat Population eTable 3. Progression of Gastric Injury and Small-Intestinal Injury From Randomization at 6 Months Through 12 Months of the Modified Intention-to-Treat Population eTable 4. Interaction Test Between Subgroups and Intervention Groups eTable 5. Progression of Gastric Injury and Small-Intestinal Injury From Randomization at 6 Through 12 Months of the Intention-to-Treat Population eFigure 1. Magnetically Controlled Capsule Endoscopy System eFigure 2. Progression of Gastric Injury From Randomization at 6 Through 12 Months eFigure 3. Progression of Small-Intestinal Injury From Randomization at 6 Through 12 Months [file jamanetwopen-e2343219-s002.pdf]

Supplementary Online Content

He C, Li Y, Jiang X, et al. Progression of gastrointestinal injury during antiplatelet therapy after percutaneous coronary intervention: a secondary analysis of the OPT-PEACE randomized clinical trial. *JAMA Netw Open*. 2023;6(11):e2343219. doi:10.1001/jamanetworkopen.2023.43219

eTable 1. Antiplatelet Medication Use at Baseline and During 12-Month Follow-Up of the Modified Intention-to-Treat Population

eTable 2. Incidence of Gastric Injury and Small-Intestinal Injury at Baseline of the Modified Intention-to-Treat Population

eTable 3. Progression of Gastric Injury and Small-Intestinal Injury From Randomization at 6 Months Through 12 Months of the Modified Intention-to-Treat Population

eTable 4. Interaction Test Between Subgroups and Intervention Groups

eTable 5. Progression of Gastric Injury and Small-Intestinal Injury From Randomization at 6 Through 12 Months of the Intention-to-Treat Population

eFigure 1. Magnetically Controlled Capsule Endoscopy System

eFigure 2. Progression of Gastric Injury From Randomization at 6 Through 12 Months

eFigure 3. Progression of Small-Intestinal Injury From Randomization at 6 Through 12 Months

This supplementary material has been provided by the authors to give readers additional information about their work.

© 2023 He C, et al. *JAMA Network Open.*

eTable 1. Antiplatelet Medication Use at Baseline and During 12-Month Follow-Up of the Modified Intention-to-Treat Population[a]

	Aspirin alone (n=132)	Clopidogrel alone (n=132)	DAPT (n=130)
**Before admission**			
No antiplatelet agent, No. (%)	58 (44.0)	52 (39.4)	45 (34.6)
Aspirin, No. (%)	72 (54.5)	80 (60.6)	85 (65.4)
P2Y12 receptor inhibitor, No. (%)	36 (27.3)	54 (40.9)	45 (34.6)
Clopidogrel	33 (25.0)	51 (38.6)	43 (33.1)
Ticagrelor	3 (2.3)	3 (2.3)	2 (1.5)
Dual antiplatelet therapy, No. (%)	31 (23.5)	51 (38.6)	43 (33.1)
**In hospital**			
Aspirin, No. (%)	132 (100.0)	132 (100.0)	130 (100.0)
P2Y12 receptor inhibitor, No. (%)	132 (100.0)	132 (100.0)	130 (100.0)
Clopidogrel	123 (93.2)	126 (95.5)	124 (95.4)
Ticagrelor	27 (20.5)	34 (25.8)	38 (29.2)
Dual antiplatelet therapy, No. (%)	132 (100.0)	132 (100.0)	130 (100.0)
**At discharge**			
Aspirin, No. (%)	132 (100.0)	132 (100.0)	130 (100.0)
P2Y12 receptor inhibitor, No. (%)	132 (100.0)	132 (100.0)	130 (100.0)
Clopidogrel	128 (97.0)	130 (98.5)	128 (98.5)
Ticagrelor	4 (3.0)	2 (1.5)	2 (1.5)
Dual antiplatelet therapy, No. (%)	132 (100.0)	132 (100.0)	130 (100.0)
**At 6 months**			
Aspirin, No. (%)	132 (100.0)	132 (100.0)	130 (100.0)
P2Y12 receptor inhibitor, No. (%)	132 (100.0)	132 (100.0)	130 (100.0)

© 2023 He C, et al. *JAMA Network Open.*

Dual antiplatelet therapy, No. (%)	132 (100.0)	132 (100.0)	130 (100.0)
**At 12 months**			
Aspirin, No. (%)	132 (100.0)	0 (0.00)	130 (100.0)
P2Y12 receptor inhibitor, No. (%)	0 (0.00)	132 (100.0)	130 (100.0)
Clopidogrel	0 (0.00)	132 (100.0)	130 (100.0)
Ticagrelor	0 (0.0)	0 (0.0)	0 (0.0)
Dual antiplatelet therapy, No. (%)	132 (100.0)	132 (100.0)	130 (100.0)
**Study medication nonadherence, No. (%)**			
Interruption	7 (5.3)	4 (3.0)	5 (3.8)
Disruption	1 (0.8)	1 (0.8)	0 (0.0)
**Gastrointestinal prophylaxis during follow-up, No. (%)**			
Proton-pump inhibitor	8 (6.1)	8 (6.1)	4 (3.1)
Others	1 (0.8)	3 (2.3)	2 (1.5)

Abbreviations: PCI = percutaneous coronary intervention, DAPT = dual antiplatelet therapy, MCE=magnetically controlled capsule endoscopy.

[a]The modified intention-to-treat population included all patients completed three MCEs and obtained three comprehensive MCE results of the stomach and the whole small intestine.

© 2023 He C, et al. *JAMA Network Open*.

eTable 2. Incidence of Gastric Injury and Small-Intestinal Injury at Baseline of the Modified Intention-to-Treat Population[a]

	**Aspirin alone (n=132)**	**Clopidogrel alone (n=132)**	**DAPT (n=130)**
Gastric injury, No. (%)			
Erosion	103 (78.0)	103 (78.0)	106 (81.5)
Ulcer	0 (0.0)	0 (0.0)	0 (0.0)
Bleeding	0 (0.0)	0 (0.0)	0 (0.0)
Small intestine injury, No. (%)			
Red Spots	75 (56.8)	66 (50.0)	67 (51.5)
Erosion	36 (27.3)	28 (21.2)	36 (27.7)
Ulcer	0 (0.0)	0 (0.0)	0 (0.0)
Bleeding	0 (0.0)	0 (0.0)	0 (0.0)

Abbreviations: DAPT = dual antiplatelet therapy.

[a]The modified intention-to-treat population included all patients completed three MCEs and obtained three comprehensive MCE results of the stomach and the whole small intestine.

© 2023 He C, et al. *JAMA Network Open.*

eTable 3. Progression of Gastric Injury and Small-Intestinal Injury From Randomization at 6 Months Through 12 Months of the Modified Intention-to-Treat Population[a]

	Aspirin vs Clopidogrel RR (98.75% CI)	Aspirin vs DAPT RR (98.75% CI)	Clopidogrel vs DAPT RR (98.75% CI)
Gastric injury			
Progression rate	0.77 (0.53-1.10)	0.70 (0.49-0.99)	0.91 (0.67-1.24)
Newly developed	0.86 (0.52-1.42)	0.82 (0.49-1.35)	0.95 (0.61-1.49)
Previous injury progression	0.73 (0.45-1.18)	0.64 (0.40-1.00)	0.88 (0.59-1.31)
Small intestine injury			
Progression rate	0.78 (0.55-1.12)	0.71 (0.50-0.99)	0.90 (0.67-1.21)
Newly developed	1.21 (0.67-2.18)	1.06 (0.63-1.79)	0.88 (0.49-1.60)
Previous injury progression	0.62 (0.39-0.98)	0.55 (0.35-0.86)	0.89 (0.63-1.24)

Abbreviations: DAPT = dual antiplatelet therapy, CI = confidence interval; RR = relative risk.

[a]The modified intention-to-treat population included all patients completed three MCEs and obtained three comprehensive MCE results of the stomach and the whole small intestine.

© 2023 He C, et al. *JAMA Network Open*.

eTable 4. Interaction Test Between Subgroups and Intervention Groups

	Freedom	Estimate	Standard Error	Wald χ^2	P[interaction]
Gastric injury					
Subgroup * Clopidogrel	1	-0.07	0.27	0.07	0.79
Subgroup * DAPT	1	-0.21	0.29	0.54	0.46
Small-intestinal injury					
Subgroup * Clopidogrel	1	-0.01	0.32	0.002	0.96
Subgroup * DAPT	1	-0.12	0.33	0.13	0.72

Abbreviations: DAPT = dual antiplatelet therapy.

*The Aspirin group was regarded as reference.

© 2023 He C, et al. *JAMA Network Open*.

eTable 5. Progression of Gastric Injury and Small-Intestinal Injury From Randomization at 6 Through 12 Months of the Intention-to-Treat Population[a]

	Aspirin vs Clopidogrel		Aspirin vs DAPT		Clopidogrel vs DAPT	
	**OR (98.75%CI)**	**P value**	**OR (98.75%CI)**	**P value**	**OR (98.75%CI)**	**P value**
Gastric injury						
All progression	0.66 (0.36-1.21)	0.09	0.73 (0.53-0.99)	0.01	0.80 (0.42-1.54)	0.39
Newly developed	0.70 (0.24-2.07)	0.42	0.76 (0.41-1.41	0.26	0.82 (0.24-2.82)	0.69
Previous injury progression	0.62 (0.30-1.29)	0.10	0.69 (0.49-0.99)	0.01	0.77 (0.37-1.60)	0.38
Small intestine injury						
All progression	0.69 (0.37-1.28)	0.13	0.73(0.53-0.99)	0.009	0.76(0.4-1.48)	0.30
Newly developed	1.39 (0.47-4.11)	0.45	1.02(0.61-1.69)	0.92	0.75(0.25-2.21)	0.50
Previous injury progression	0.48 (0.23-0.99)	0.01	0.61(0.41-0.92)	0.002	0.79(0.36-1.75)	0.45

Abbreviations: DAPT = dual antiplatelet therapy.

* The P values and odd ratios were estimated by multiple imputation, and marginal estimates are reported.

© 2023 He C, et al. *JAMA Network Open.*

eFigure 1. Magnetically Controlled Capsule Endoscopy System

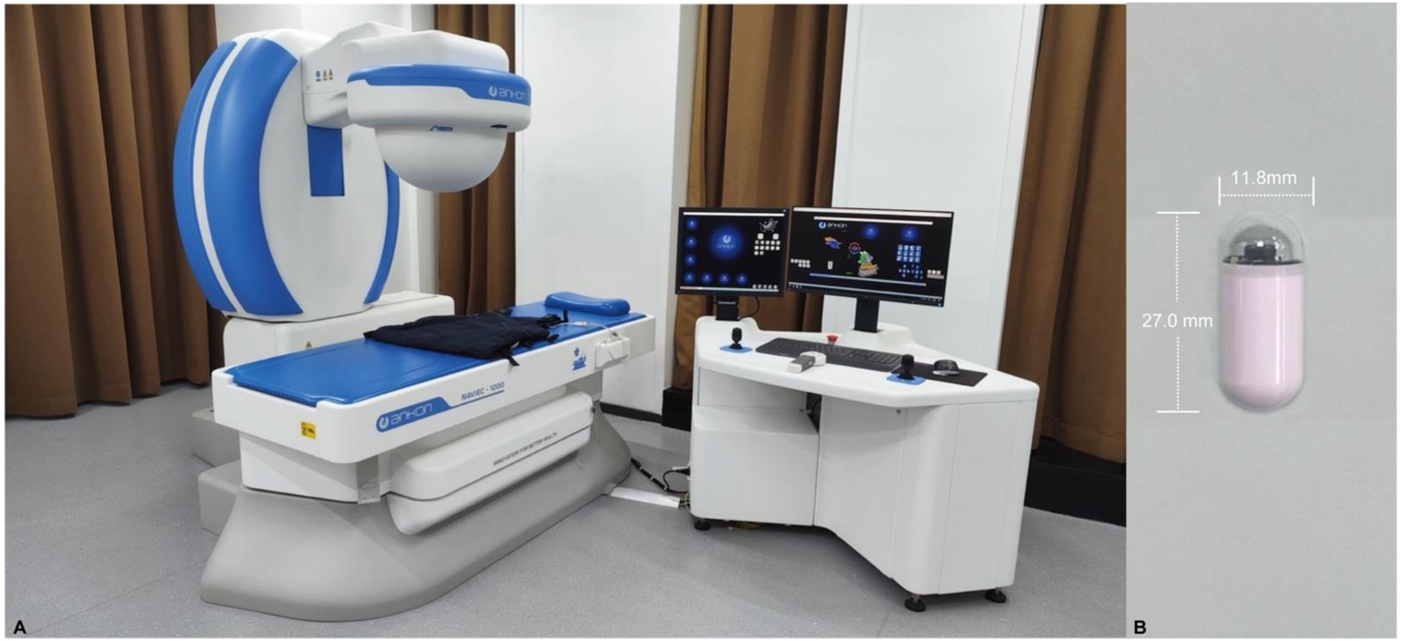

A) A navigation system, a capsule locator, an ESNavi software and a portable data recorder. **B**) A capsule endoscopy.

© 2023 He C, et al. *JAMA Network Open*.

eFigure 2. Progression of Gastric Injury From Randomization at 6 Through 12 Months

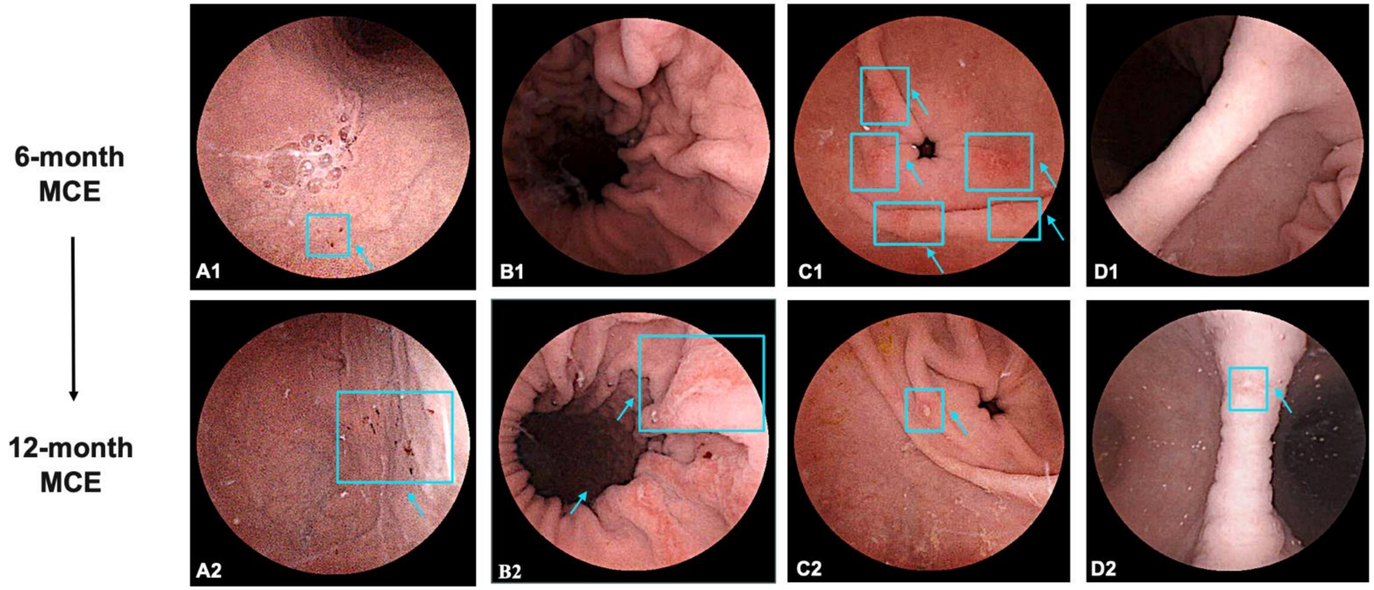

Imaging findings from four patients (A, B, C and D) are shown. **A1-A2**) Progression of erosions in the gastric fundus. **B1-B2**) Progression of ulcers in the gastric body. **C1-C2**) Progression from erosion to ulcer in the gastric antrum. **D1-D2**) Progression of ulcers in gastric angulus.

© 2023 He C, et al. *JAMA Network Open.*

eFigure 3. Progression of Small-Intestinal Injury From Randomization at 6 Through 12 Months

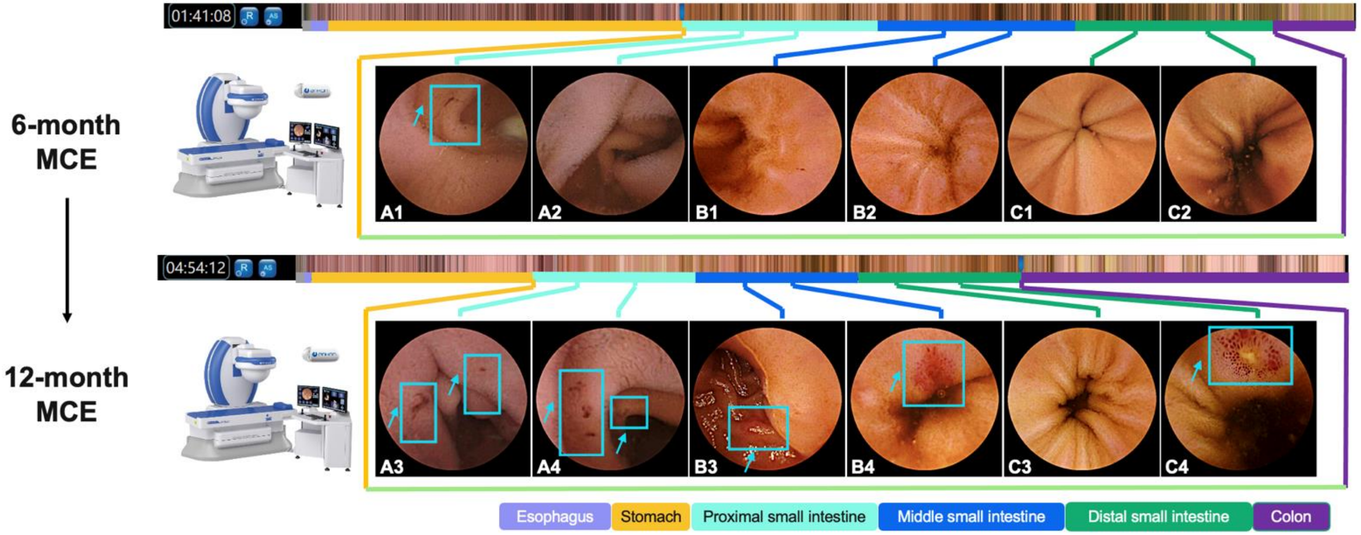

Imaging findings from three patients (A, B and C) are shown. **A1-A4**) Progression of red spots in the proximal small intestine. **B1-B4**) Progression of erosions in the middle small intestine. **C1-C4**) Progression of ulcers in the distal small intestine.

© 2023 He C, et al. *JAMA Network Open.*
