# Supplementary material for: Progression of Gastrointestinal Injury During Antiplatelet Therapy After Percutaneous Coronary Intervention: A Secondary Analysis of the OPT-PEACE Randomized Clinical Trial
Source: JAMA Netw Open. 2023 Nov 17;6(11):e2343219. doi: 10.1001/jamanetworkopen.2023.43219 (PMC10656648; doi:10.1001/jamanetworkopen.2023.43219)
Supplement: Supplement 3. — Data Sharing Statement [file jamanetwopen-e2343219-s003.pdf]

Data Sharing Statement

He. Progression of Gastrointestinal Injury During Antiplatelet Therapy After Percutaneous Coronary Intervention. *JAMA Netw Open*. Published November 17, 2023. doi:10.1001/jamanetworkopen.2023.43219

Data

Data available: No. Deidentified individual participant data that underlie the reported results will be made available on request from the corresponding authors. The study protocol is included as a data supplement available with the online version of this article.
